# *Trypanosoma brucei* Parasites Occupy and Functionally Adapt to the Adipose Tissue in Mice

**DOI:** 10.1016/j.chom.2016.05.002

**Published:** 2016-06-08

**Authors:** Sandra Trindade, Filipa Rijo-Ferreira, Tânia Carvalho, Daniel Pinto-Neves, Fabien Guegan, Francisco Aresta-Branco, Fabio Bento, Simon A. Young, Andreia Pinto, Jan Van Den Abbeele, Ruy M. Ribeiro, Sérgio Dias, Terry K. Smith, Luisa M. Figueiredo

**Affiliations:** 1 Instituto de Medicina Molecular, Faculdade de Medicina, Universidade de Lisboa, 1990-375 Lisboa, Portugal; 2 Department of Neuroscience, University of Texas Southwestern Medical Center, Dallas, TX 75390-9111, USA; 3 Graduate Program in Areas of Basic and Applied Biology, Instituto de Ciências Biomédicas Abel Salazar, Universidade do Porto, 4099-002 Porto, Portugal; 4 Theoretical Division, Los Alamos National Laboratory, Los Alamos, NM 87545, USA; 5 Department of Biomedical Sciences, Unit of Veterinary Protozoology, Institute of Tropical Medicine Antwerp, B-2000 Antwerp, Belgium; 6 Department of Physiology, Laboratory of Zoophysiology, University of Ghent, B-9000 Ghent, Belgium; 7 Biomedical Sciences Research Complex, University of St Andrews, North Haugh, St Andrews, Fife KY16 9ST, UK; 8 Guest Professor, Faculdade de Medicina, Universidade de Lisboa, 1990-375 Lisboa, Portugal

**Keywords:** African trypanosomes, fat, mouse infection, fatty acid β-oxidation, metabolism, transcriptome

## Abstract

*Trypanosoma brucei* is an extracellular parasite that causes sleeping sickness. In mammalian hosts, trypanosomes are thought to exist in two major niches: early in infection, they populate the blood; later, they breach the blood-brain barrier. Working with a well-established mouse model, we discovered that adipose tissue constitutes a third major reservoir for *T. brucei*. Parasites from adipose tissue, here termed adipose tissue forms (ATFs), can replicate and were capable of infecting a naive animal. ATFs were transcriptionally distinct from bloodstream forms, and the genes upregulated included putative fatty acid β-oxidation enzymes. Consistent with this, ATFs were able to utilize exogenous myristate and form β-oxidation intermediates, suggesting that ATF parasites can use fatty acids as an external carbon source. These findings identify the adipose tissue as a niche for *T. brucei* during its mammalian life cycle and could potentially explain the weight loss associated with sleeping sickness.

## Highlights

• *T. brucei* parasites accumulate in the adipose tissue early after mouse infection
• Adipose tissue forms (ATFs) can replicate and are capable of infecting naive mice
• ATFs are transcriptionally distinct and upregulate genes for fatty acid metabolism
• ATFs can actively uptake exogenous myristate and form β-oxidation intermediates

## Graphical Abstract

**Figure:**
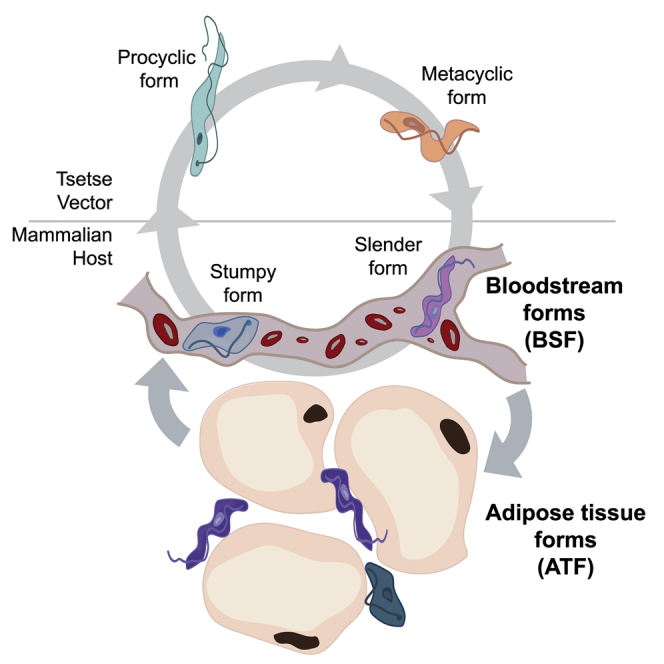

## Teaser

*Trypanosoma brucei* is found in the bloodstream and interstitial compartment of several organs in the mammalian host. Trindade et al. uncover the adipose tissue as a major extravascular parasite niche. Extensive remodeling of parasite gene expression in this lipid-rich environment includes upregulation of fatty acid β-oxidation enzymes, suggestive of a functional adaptation.

## Introduction

Human African trypanosomiasis (HAT), also known as sleeping sickness, is a neglected tropical disease that is almost always fatal if left untreated. This disease is caused by *Trypanosoma brucei*, a unicellular parasite that lives in the blood, lymphatic system, and interstitial spaces of organs (reviewed in Kennedy, 2013). Disease pathology often correlates with sites of accumulation of the infectious agent within its host, including the brain, which is associated with characteristic neuropsychiatric symptoms and sleep disorder. Weight loss is another typical clinical feature of sleeping sickness pathology (Kennedy, 2013), but is essentially unstudied.

*T. brucei* is transmitted through the bite of a tsetse and quickly adapts to the mammalian host to become what is known as a “slender” bloodstream form (BSF). As parasitemia increases, slender forms are capable of sensing population density, and this triggers differentiation to the stumpy form, which is pre-adapted to life in the transmitting tsetse vector and, once there, further differentiates into procyclic form (PCF). Several studies have shown 10%–30% of genes being differentially expressed between BSFs and PCFs (reviewed in Siegel et al., 2011), including genes involved in metabolism, organelle activity, cell-cycle regulation, and endocytic activity. Recent proteomic studies also revealed around 33% of proteins that are developmentally regulated (Butter et al., 2013).

A major difference between BSFs and PCFs is their energy production, with the former utilizing glucose via glycolysis within the glycosome and the latter utilizing proline and, to a lesser extent, other amino acids as their carbon source, via the Krebs cycle in mitochondrion (reviewed in Szöör et al., 2014). To date, no fatty acid β-oxidation has been observed as a carbon source in any life cycle stage of this parasite. This has been a puzzling observation, as the genes required for productive β-oxidation, including the carnitine-acyltransferases (for mitochondrial import of fatty acids), are present in the genome.

Here, we describe an additional form of *T. brucei* in mammalian hosts: we demonstrate that *T. brucei* accumulates in adipose tissue, consistent with recent studies showing accumulation of parasites in the lower abdomen (Claes et al., 2009, McLatchie et al., 2013). Adipose tissue resident *T. brucei* have a different metabolic profile from either slender or stumpy forms in the blood, and this profile is consistent with their utilization of fatty acids (myristate) as a carbon source. These experiments describe an additional form of *T. brucei* life cycle and possibly explain weight loss (wasting), one of the characteristic pathological features of sleeping sickness.

## Results

### *T. brucei* Parasites Are Heterogeneously Distributed in Mice

**Figure 1:**
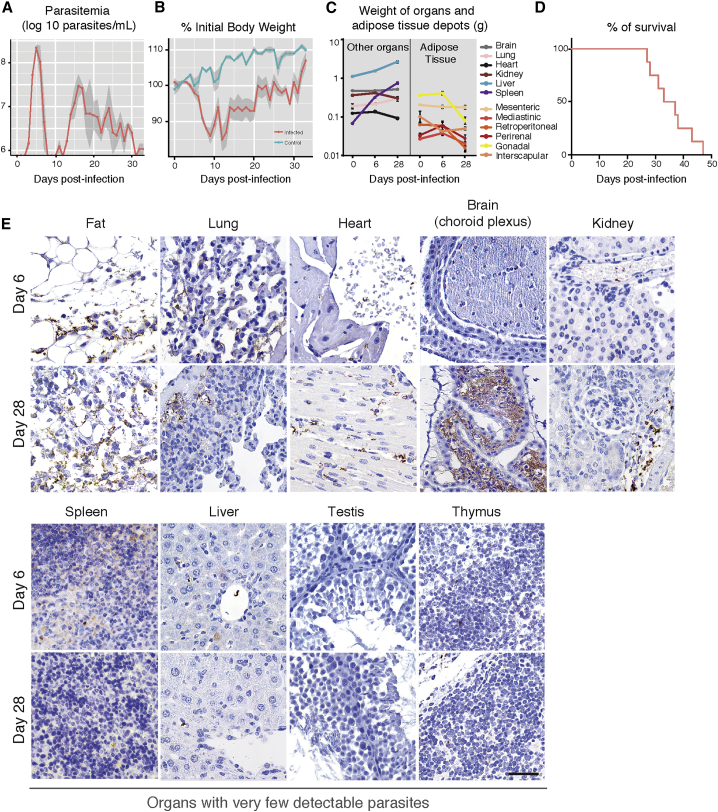
Tissue Distribution of *T. brucei* during a Mouse Infection Is Heterogeneous (A) Mean parasitemia profile of 20 mice infected with *T. brucei* AnTat1.1^E^. Parasitemia was assessed from tail blood using a hemocytometer (limit of detection is around 4 × 10^5^ parasites/mL). Light gray shaded area represents SEM. (B) Variation of body weight during infection. Daily body weight measurement of control and infected mice (n = 15 per group). Light gray shaded area represents SEM. (C) Variation of organ weight during infection (n = 4 per group). (D) Survival curve of *T. brucei* infected mice (n = 8). (E) Representative brightfield micrographs of *T. brucei* distribution in several organs/tissues at days 6 and 28 post-infection, assessed by immunohistochemistry with a non-purified rabbit anti-VSG antibody (parasites appear in brown). n = 5 per time point. Scale bar, 50 μm. See also Figures S1 and S2.

The well-established mouse model (C57BL/6J mice with a pleomorphic clone AnTat1.1^E^) was used to confirm weight loss during infection as observed in humans with sleeping sickness. Parasitemia followed a previously described pattern: the first peak of parasitemia occurred 5–6 days post-infection, at around 2 × 10^8^ parasites/mL, and after approximately 4 days of undetectable parasitemia, parasites could be detected again with a fluctuating parasitemia of 10^6^–10^7^ parasites/mL (Figure 1A). After the first peak of parasitemia, all infected animals showed reduced food intake and a 10%–15% decrease in body weight. Eventually, all mice recovered normal food intake, although their body weight remained 5% lower than that of non-infected animals (Figures 1B and S1A, available online). The weight of most organs from mice sacrificed on days 6 and 28 post-infection showed minimal changes relative to day 0, except fat depots, which decreased on average 43% ± 12%. Spleen size and weight increased dramatically as previously reported (Figure 1C). Infected mice died 35 ± 2.5 days post-infection (Figure 1D).

To assess the parasite load in different organs, we used immunohistochemistry at different days of infection (Figures 1E and S1B). Parasites were consistently detected in the fat 6 days post-infection and at later time points, while in other organs they were seen sporadically and at very low densities. As infection progressed, we observed an increase in parasite load in most organs, with fat, heart, brain, lung, and kidney being the most visibly infiltrated. Parasites were always found extracellularly within the interstitium of these organs. In the brain, our data corroborate the extensively reported evidence for the localization of parasites being restricted to the choroid plexuses and meninges (Kennedy, 2013) (Figure 1E).

Histologically, thymus, lymph nodes, bone marrow, skin (of the head and neck), salivary glands, spleen, gastrointestinal mucosa, testis, and liver displayed few or no parasites (Figure 1E; data not shown). Although parasites in the stroma of the testis were absent, the epidymal fat body and stroma of the epididymis (a small paired organ in the posterior end of the testis) contained a significant number of parasites, many of which appeared as debris, but which could explain the bioluminescence detected by Claes et al. (2009).

### Early in Infection, *T. brucei* Accumulate in Adipose Tissue

**Figure 2:**
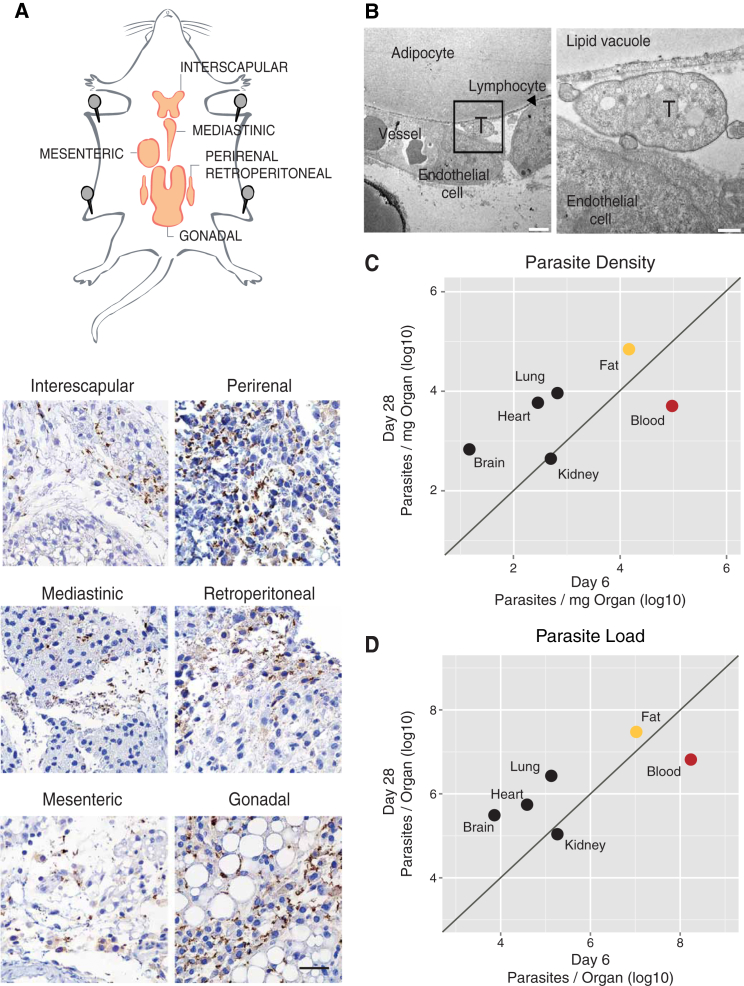
Fat Depots Are a Major Parasite Reservoir (A) Schematic representation of mice fat depots and anti-VSG immunohistochemistry images of six different fat depots, collected 28 days post-infection. Scale bar, 50 μm. (B) Transmission electron micrograph of a gonadal fat depot 6 days post-infection. Trypanosome (T) and lymphocyte in the interstitial space, adjacent to an adipocyte and next to a small capillary. Scale bars, 2 and 0.5 μm in the left and right panels, respectively. (C) Parasite density in multiple organs/tissues (6 and 28 days post-infection) was measured by qRT-PCR of gDNA (quantification of *T. brucei* 18s rDNA relative to the tissue/organ weight). Blood density was assumed 1.05 g/mL. Fat value is the average of quantification of the six depots indicated in (A). Each point represents the geometric mean of the parasite density on days 6 (n = 3–9) and 28 post-infection (n = 3–6). (D) Parasite load in multiple organs/tissues estimated by multiplying parasite density with organ weight at the corresponding day of infection. Each point represents the geometric mean of the parasite density on days 6 (n = 3–9) and 28 post-infection (n = 3–6). See also Figures S1–S3.

Immunohistochemical staining showed parasites in the stroma of several fat depots: gonadal, mediastinic, mesenteric, retroperitoneal, perirenal, and interscapular (Figure 2A). Transmission electron microscopy (TEM) confirmed that these parasites were indeed extravascular, as numerous trypanosomes were observed in the interstitial space, either between adjacent adipocytes or between the adipocytes and the capillaries (Figure 2B).

To quantify parasite density, we used as a proxy Trypanosome genomic DNA (gDNA), which was quantified at 6 and 28 days post-infection in the organs/tissues where parasites had been detected by histology, i.e., fat, lung, heart, kidney, brain, and blood (Figures 2C and S2A). The blood had the highest parasite density on day 6. Among solid organs/tissues, for the same day of infection, parasite density was relatively low, except for fat, which had on average 60-fold more parasites than lung, heart, kidney, and brain and 7-fold less than blood. On day 28 of infection, parasite density remained equally high in fat (10^4^–10^5^ parasites/mg), while it increased, on average, 20-fold in the brain, heart, and lung (Figure 2C). The overall high parasite density was detected in all fat depots characterized in this study, with no significant differences between white and brown fat depots (Figure S2B; Supplemental Experimental Procedures). The blood was the only site where we observed a decrease in parasite density during infection, which is consistent with parasitemia dynamics (Figures 1A and 2C). As a consequence, on day 28, fat was the compartment with the highest parasite density (linear mixed-effects model [LME], p < 0.0001).

Overall, the density of parasites per milligram of organ/tissue correlated well with the density calculated as a ratio of parasite gDNA versus mouse gDNA in each tissue (Figure S2C). We also observed essentially the same pattern of parasite density and the same preferential accumulation in the fat when we quantified parasite RNA (qRT-PCR) instead of DNA (Figure S2D), suggesting that gDNA quantification reflects accurately the number of live parasites. Immunohistochemistry also showed accumulation of parasites in fat regardless of parasite strain (EATRO1125 AnTat1.1^E^, Lister 427), infection route (intraperitoneal, intravascular), mouse strains (C57BL/6J, BALB/c), animal gender (male, female), or rodent species (mice, rat) (Figure S2E).

Fat represents around 14% of the body weight of a healthy mouse (Jackson Mouse Phenome Database); thus, it is potentially a very large reservoir of parasites. The number of parasites in the organs/tissues (parasite load) was determined by multiplying parasite density by the weight of the organ at the corresponding time of infection. For fat, we used the weight of the six depots characterized in this work, which comprises around 25% of the total body fat. We observed that while 6 days post-infection, the blood contained the majority of the parasites (around 10^8^ parasites), on day 28 the six depots of fat contained overall more parasites than the blood, brain, and all other tested organs combined (LME, p < 0.0001) (Figure 2D). A similar preference for accumulation in fat was observed when the mouse infection was initiated by a tsetse bite, which deposits metacyclic forms in the skin of the mouse (Figure S3) (LME, p < 0.0001). Overall, these data revealed that fat represents a major reservoir of parasites, regardless of whether the infection was initiated by BSFs or metacyclic forms.

### Adipose Tissue Contains Replicative and Infective Parasites

**Figure 3:**
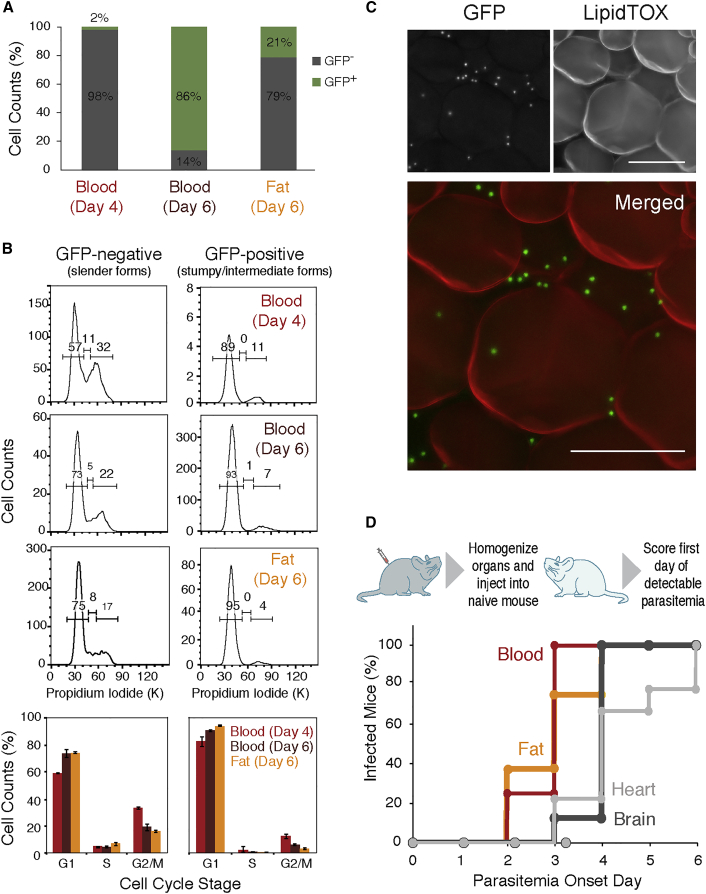
Fat Harbors Replicative Forms that Can Establish a New Infection (A) Frequency of GFP expression measured by flow cytometry in parasites isolated from blood and fat, 4 and 6 days post-infection with a *GFP::PAD1*_*utr*_*T. brucei* reporter cell line (n = 2–3). (B) Cell-cycle analysis assayed by flow cytometry of propidium iodide-stained parasites (n = 2–3). The values represented are the means of the percentage of the cell population in each cell-cycle stage and their SEM. (C) Fluorescence microscopy of gonadal adipose tissue from a mouse infected for 6 days with *GFP::PAD1*_*utr*_ reporter cell line. Lipid droplets were stained with LipidTOX (red), and nuclei of GFP-expressing parasites (stumpy and/or intermediate forms) are green. Scale bar, 50 μm. (D) Onset of parasitemia curves in mice that were injected intraperitoneally with infected organs/tissues lysates from a donor mouse. Lysates from blood, heart, brain, and gonadal fat depot were prepared from mice sacrificed between 21 and 28 days post-infection to ensure presence of a larger number of parasites (n = 9). See also Figure S4.

In the blood, parasites can be either replicative slender, G1-arrested stumpy forms or intermediate forms that are not fully differentiated (reviewed in MacGregor et al., 2012). To investigate whether the parasites from fat (referred to hereafter as adipose tissue forms [ATFs]) are replicative or not, we infected mice with a *GFP::PAD1*_*utr*_ reporter cell-line, in which a *GFP* gene is followed by a *PAD1* 3′ UTR that confers maximum expression in stumpy forms (J. Sunter, A. Schwede, and M. Carrington, personal communication; MacGregor et al., 2012). Four and six days post-infection, blood and fat were collected, and parasites isolated and purified. As described by MacGregor et al. (2011), on day 4 we observed that most parasites in the blood were GFP negative (98% ± 0.3%), while on day 6 most parasites expressed high levels of GFP (86% ± 2.6%). Interestingly, from day 5, we noted the presence of parasites expressing lower levels of GFP, which likely correspond to differentiating intermediate forms (data not shown). As the yield of isolation of ATF parasites was very low on days 4 and 5, we analyzed these parasites only on day 6. The majority of the ATF parasites were GFP negative (79% ± 4.6%), while 21% ± 4.6% expressed GFP, indicating that on day 6 fat contains fewer stumpy/intermediate forms than blood (Figure 3A).

To confirm if GFP-negative ATF parasites are replicative and GFP-positive parasites are cell-cycle arrested, we stained the parasite nuclear DNA with propidium iodide and quantified it by flow cytometry. In all samples (blood day 4, blood day 6, and fat day 6), we observed that GFP-negative parasites displayed a cell-cycle profile characteristic of replicative cells (around 60%–70% of cells in G1, 5% in S-phase and 20%–30% in G2/M), while GFP-positive cells were cell-cycle arrested in G1/G0 (90%–95%) (Figure 3B). Similar data were obtained by performing cell-cycle analysis with DyeCycle Violet (Figures S4A and S4B), further confirming the presence of slender and stumpy/intermediate forms in fat.

To validate the presence of stumpy/intermediate forms in fat, we used fluorescence microscopy on an intact gonadal depot infected with *GFP::PAD1*_*utr*_-expressing parasites (Figure 3C). LipidTOX stains the lipids in the large lipid droplet of adipocytes. Among the adipocytes, we could clearly observe many green foci, which represent the parasite nuclei where GFP accumulates, thus confirming the presence of GFP-positive parasites (stumpy and/or intermediate forms) in close proximity to adipocytes. The presence of replicating parasites in intact tissue was confirmed by immunohistochemistry with an anti-H2A antibody. Dividing nuclei were clearly visible in close proximity to adipocytes (Figure S4C), further confirming the fluorescence-activated cell sorting (FACS) cell-cycle data.

To test whether ATF parasites are capable of establishing a new infection, an infected donor mouse was sacrificed and perfused, and several organs were collected, homogenized, and injected intraperitoneally into recipient naive mice. Parasitemia was assessed daily thereafter and scored on the first day it became detectable (Figure 3D). Mice that received blood or a fat homogenate showed parasitemia earlier (around 3 days post-transplantation) than animals injected with heart and brain homogenates (around 4 days post-transplantation), consistent with the observed parasite load in these organs (Figures 2D and 3D). Transplant of intact gonadal fat depot also led to successful infection of the recipient naive mice (data not shown), suggesting that parasites can exit from an intact tissue. These results showed that parasites from fat, heart, and brain are capable of reinvading the bloodstream and establishing a new infection.

### Morphology of Adipose Tissue Forms

**Figure 4:**
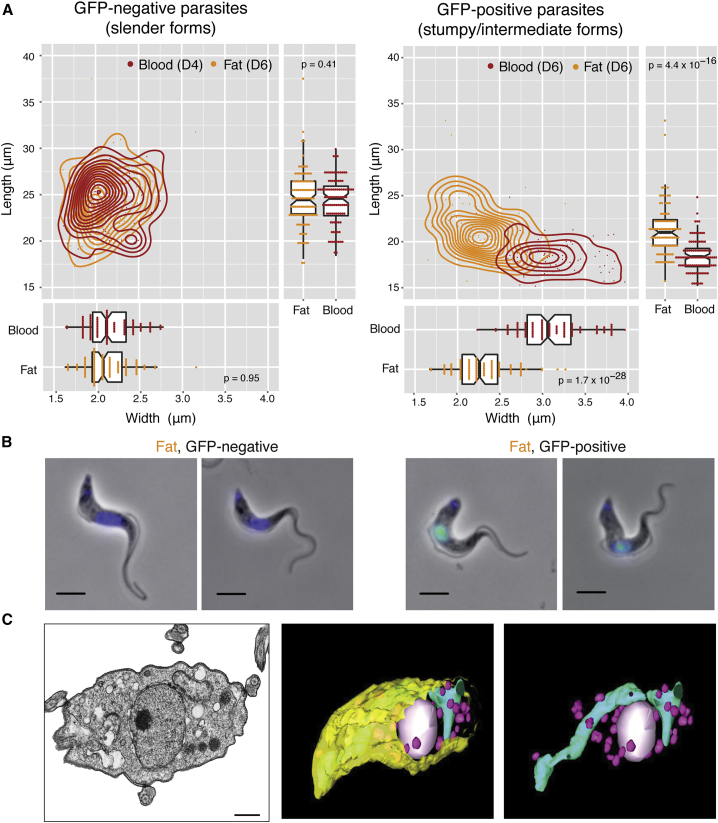
Fat Is Populated by Slender, Intermediate, and Stumpy Forms (A) Morphological features (length and width) of fixed parasites isolated from fat and blood of mice infected with *GFP::PAD1*_*utr*_ reporter. Fat gonadal tissue was collected on day 6 post-infection. The blood “controls” were obtained as follows: GFP-negative parasites were collected on day 4 post-infection (mostly slender forms), and GFP-positive parasites were collected on day 6 post-infection (mostly stumpy forms). Morphometric measurements were scored from phase contrast microscopy images, analyzed via HTIAoT, and confirmed by manual measurement. GFP negative, slender form; GFP positive, stumpy and intermediate forms. n = 100 per group, from three independent mouse experiments. Statistical significance was assessed using a Wilcoxon rank-sum test. (B) Representative images of parasites isolated from fat. Replicating parasites (such as the second from the left) were excluded from morphometric analysis. DNA was stained with DAPI (blue). GFP protein (green) is localized in the nucleus of intermediate and stumpy forms. Scale bar, 4 μm. (C) Transmission electron micrograph and 3D tomography images of a parasite isolated from gonadal adipose tissue. Mitochondrion is represented in cyan, glycosomes in pink, nucleus in white, and plasma membrane in yellow. Scale bar, 500 nm. See also Figure S5 and Movie S1.

Although *T. brucei* is always extracellular, its morphology changes during the life cycle, which may reflect a specific adaptation to the host niche (Wheeler et al., 2013, Bargul et al., 2016). ATF parasites, like BSFs, have an undulating appearance, with a flagellum attached to the cell body and with kinetoplast DNA positioned between nucleus and flagellar pocket (Figure 4B).

To characterize in more detail the morphology of ATF parasites, we compared the length and width of *GFP::PAD1*_*utr*_ parasites isolated from adipose tissue (day 6 post-infection) and blood (days 4 and 6 post-infection) (Figures 4A and 4B). Automatic measurements of phase contrast microscopy images were generated via HTIAoT (Wheeler et al., 2012) and confirmed with manual measurements (Figure S5A). We observed that slender forms (GFP negative) from blood and adipose tissue were very similar both in length (blood, 24.39 ± 2.50 μm; fat, 24.57 ± 2.99 μm) and width (blood, 2.15 ± 0.26 μm; fat, 2.12 ± 0.26 μm). This average length is consistent with previous reports (Tyler, 1998, Tyler et al., 2001, Bargul et al., 2016). In blood day 6 post-infection, GFP-positive parasites were, as expected, shorter (18.43 ± 1.81 μm) and wider (3.11 ± 0.38 μm) than slender counterparts of day 4, corresponding to the morphology of stumpy forms (Tyler et al., 2001). Interestingly, in adipose tissue we found not only stumpy forms, but also GFP-positive parasites that morphologically were in between slender and stumpy forms (length, 21.32 ± 2.73 μm; width, 2.29 ± 0.31 μm) (Tyler, 1998). These probably correspond to the previously described blood intermediate forms, which, as the name suggests, are not fully differentiated into stumpy forms, but could already express GFP::PAD1 (MacGregor et al., 2011, MacGregor et al., 2012). These results indicate that adipose tissue is populated by parasites whose morphology has been previously found in the blood. The only significant difference is their relative distribution: on day 6 of infection, while blood is mostly populated by stumpy forms, adipose tissue appears to be “delayed,” as we detected both intermediate and stumpy forms.

At the ultrastructure level, ATF parasites contain all major structures described in other stages of life cycle (Gull, 1999), including an electron-dense coat, nucleus, mitochondrion, endoplasmic reticulum, Golgi apparatus, glycosomes, dense granules and numerous vesicles compatible with endosomes, an internal subpellicular corset of microtubules underneath plasma membrane, and a flagellum attached to the cell body (Figures 4C and S5B). Using serial 3D tomography, we observed that the single mitochondrion of ATF parasites occupies a small volume of parasite body and is not highly branched (Figure 4C; Movie S1). This organization was confirmed by Mitotracker Green staining, which showed no major differences between the mitochondrion of parasites in blood and adipose tissue (Figure S5C; Movie S1).

### Transcriptome of ATF Parasites Reveals Differences in Several Key Regulatory Processes

During its life cycle, *T. brucei* adapts to its environment by changing gene expression (reviewed in Siegel et al., 2011). To test whether parasites within fat also adapted to the new environment, total RNA was extracted from infected gonadal fat depot (n = 3) on day 6, along with parasites from blood (n = 2) on day 4 (maximizing slender and minimizing stumpy/intermediate forms), and was subjected to RNA sequencing (RNA-seq) analysis. As expected, sequence reads from blood samples corresponded mainly to parasite transcripts, while sequence reads from fat corresponded mainly to host transcripts. Nevertheless, the 1%–9% of the sequence reads from *T. brucei* provided enough statistical power to detect changes in the transcriptomes of ATF parasites (Table S1). Two previously published RNA-seq datasets of BSF parasites grown in culture were also included in this analysis (Pena et al., 2014).

**Figure 5:**
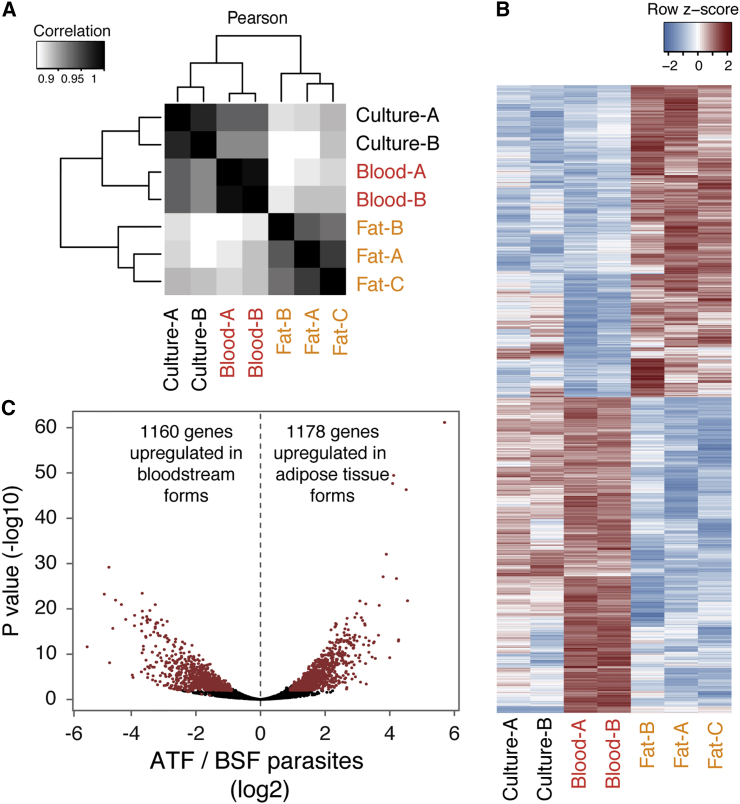
ATF Parasites Are Transcriptionally Different from BSFs (A) Hierarchically clustered heat map of Pearson correlations of transcript levels (log_2_ transformed RPKM) from independent RNA-seq datasets: Lister427 parasites grown in culture (Pena et al., 2014) (n = 2), parasites isolated from blood of AnTat1.1-infected mice on day 4 post-infection (n = 2), and parasites isolated from gonadal fat on day 6 post-infection (n = 3). (B) Heat map view of relative transcript levels for differentially expressed genes from culture and in vivo in parasites isolated from the two tissues (adjusted p < 0.01 in at least two of three methods). (C) Volcano plot displaying in red the differentially expressed genes represented in (B). Displayed p values and fold changes are from DESeq2. See also Tables S1 and S2.

Unbiased clustering of gene expression profiles revealed that ATF parasites clustered separately from parasites isolated from blood or culture (Figure 5A), suggesting significant changes in their overall transcriptome. Changes were identified using three methods of differential expression analysis, and only those genes identified by at least two methods with an adjusted p value <0.01 were considered. These analyses showed that 2,328 genes (around 20% of transcriptome) were differentially expressed between BSF and ATF parasites: 1,160 were upregulated in ATF parasites and 1,178 were upregulated in BSFs (Figures 5B and 5C; Table S2).

Significant changes were found in genes involved in gene expression regulation, cell cycle, and cell signaling (Table S3). RNA-binding proteins play an important role in gene expression and differentiation throughout the *T. brucei* life cycle. For example, RNA-binding protein 42 (RBP42; TriTrypDB: Tb927.6.4440, http://tritrypdb.org/tritrypdb/) binds many mRNAs involved in cellular energy metabolism (Das et al., 2012). Upregulation of RBP42 in ATF parasites could be involved in the metabolic rewiring when parasites enter the fat (Table S3).

ATF parasite transcriptome also showed dramatic changes in gene expression of various post- and co-translational modifying enzymes that might have considerable influence on diverse cellular processes (Table S3). A small number of genes potentially acting in the cell cycle and cytokinesis was identified with significant differential expression, including the cytoskeleton-associated AIR9 protein and spastin, which were upregulated (Table S3), suggesting differences in cell-cycle regulation in these parasites. Consistent with a minor stumpy form population in fat, we did not find enrichment of stumpy-specific genes in the transcriptome of ATF parasites. Extracellular signaling mechanisms also seem to be affected in ATF parasites, including upregulation of TOR3, which can relate the supply of external nutrients to internal energy levels to regulate cellular growth (de Jesus et al., 2010).

Interestingly, although by TEM an electron-dense coat can be observed around the parasite (Figure 2B), we found that the transcript levels of the active variant surface glycoprotein (VSG) (VSG AnTat1.1, CAA25971.1) are 3-fold downregulated in adipose tissue, suggesting VSG downregulation or VSG switching within the tissue. As the VSGnome of AnTat1.1E clone is currently unknown, we could not test whether silent VSGs were upregulated as a compensatory mechanism. Genes encoding for other surface molecules, such as the haptoglobin receptor and most procyclins, were not differentially expressed.

### Transcriptome of ATF Parasites Reveals Metabolic Adaptations

One of the most evident changes in ATF transcriptome was the upregulation of many metabolic pathways, including glycolysis, pentose phosphate, purine salvage, sterol and lipid metabolism, and, surprisingly, β-oxidation. Thirteen of the 14 enzymatic steps of glycolysis were upregulated relative to BSFs (Table S3). This may either be a response to the lower glucose concentration in fat interstitial fluid relative to bloodstream, or an upregulated gluconeogenesis, which relies mostly on the same enzymes.

In ATF parasites, genes involved in three out of the five biosynthetic steps in the pentose-phosphate pathway were upregulated, including the rate-limiting glucose-6-phosphate dehydrogenase (TriTrypDB: Tb927.10.2490). This observation, taken together with the fact that numerous enzymes (16 in total) involved in purine salvage pathway were also upregulated, suggests that ATF parasites may increase purine production. Interestingly, the purine phosphatases (TriTrypDB: Tb927.8.3800 and Tb927.7.1930) and cAMP phosphodiesterase PDEA (TriTrypDB: Tb927.10.13000) are up- and downregulated, respectively, suggesting that the increased purine production may be directed toward cAMP signaling (Table S3).

ATF parasites showed significant upregulation of the alanine and aspartate aminotransferases and the glutamate shunt, which feed products into the tricarboxylic acid (TCA) cycle. Additionally, this cycle also appeared to be more active, given the upregulation in three key steps, allowing it to process succinate, fumarate, and 2-oxoglutarate, resembling the TCA cycle of the *T. brucei* insect form (reviewed in Szöör et al., 2014). These changes suggest that the F0/F1 ATP synthase complex is functional and that the associated electron transport chain is operating in ATF parasites in a manner similar to that in PCF parasites.

**Figure 6:**
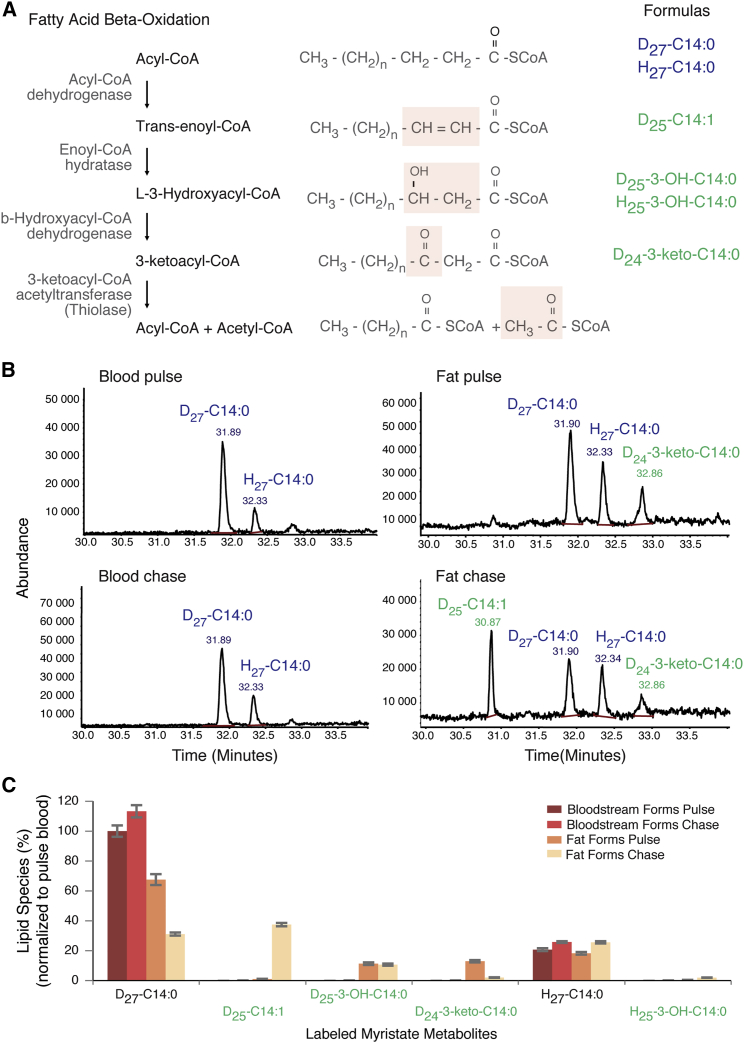
Fatty Acid β-Oxidation Is Active in ATF Parasites (A) Schematic of fatty acid β-oxidation pathway. Four enzymatic modifications are indicated by shaded box on the fatty acid structures where biotransformation takes place. Formulas in blue and green indicate the myristate and β-oxidation metabolites from the non-labeled and labeled myristate, respectively, identified in this work. (B) Fatty acid methyl ester (FAME) analysis by GC-MS of D_27_-C14:0-labeled BSF (left) or ATF (right) parasites for 1 hr (upper) and chased for a further 1 hr (lower). GC-MS trace shows 30–34 min (n = 3). (C) Uptake of D_27_-C14:0 and β-oxidation metabolites after normalization to the added internal standard C17:0. 100% equates to the amount of D_27_-C14:0 taken up by bloodstream form in the 1 hr labeling (pulse) (n = 3). The values represented are the means and the respective SEM. See also Figures S6 and S7.

Significant changes in gene expression of lipid and sterol metabolic pathways were also observed in ATF parasites. However, one of the most striking observations in the RNA-seq data was the potential presence of an active fatty acid β-oxidation, which produces energy from fatty acid catabolism. This was unexpected, as β-oxidation activity has never been detected in any *T. brucei* life cycle stage to date. ATF parasites showed upregulation of the putative genes responsible for the second and fourth steps of the β-oxidation cycle (enoyl-CoA hydratase and 3-ketoacyl-CoA thiolase, respectively) (Figure 6A). Moreover, fatty acid transport across the mitochondrial membrane (facilitated by acyl-CoA synthases and carnitine-acyltransferases) was upregulated, while fatty acid elongases 2 and 4 were downregulated, suggesting that in ATF parasites, endocytosed fatty acids are not being elongated and anabolized into more complex molecules. Instead, they may enter the glycosomes and/or mitochondrion, where they are catabolized via a β-oxidation pathway to form acetyl-CoA (experimentally validated; see below and Figure 6), which feeds into the now-active TCA cycle.

### ATF Parasites Have Active Fatty Acid β-Oxidation

To investigate whether ATF parasites are capable of β-oxidation, labeled myristate was used in a pulse-chase experiment with living trypanosomes, and potential labeled β-oxidation intermediates were identified by gas chromatography-mass spectrometry (GC-MS). Myristate (C14:0) was chosen, as it is efficiently taken up and incorporated into lipids and glycosylphosphatidylinositol (GPI) anchors in the slender BSF parasites (reviewed in van Hellemond and Tielens, 2006). Isolated ATF parasites were labeled with deuterated myristate (D_27_-C14:0) for 1 hr and then chased with serum, following which labeled myristate metabolites were identified. As expected, BSFs showed accumulation of D_27_-C14:0 during the pulse and chase periods (Figures 6B and 6C). ATF parasites also showed D_27_-C14:0 accumulation during the pulse (Figure 6B, upper panel, and Figure 6C), but the amount of D_27_-C14:0 decreased significantly during the chase (Figure 6B, lower panel, and Figure 6C). Importantly, the decrease of D_27_-C14:0 in ATF parasites coincided with the detection of β-oxidation metabolites derived from the labeled myristate, including myristoleic acid (D_25_-C14:1), 3-hydroxy-myristate (D_25_-3-OH-C14:0), and 3-oxo-myristic acid (D_24_-3-keto-C14:0) (Figure 6B, right panels, and Figures 6C, S6, and S7). The latter two metabolites were also observed to some minor extent during the pulse (Figure 6B, upper panel, and Figure 6C), while D_25_-C14:1 was present in higher amount during the chase period. Minor amounts of unlabeled 3-hydroxy-myristrate (H_26_-3-OH-C14:0) were also observed in ATF parasites, but not BSFs (Figure 6C).

Collectively these data show that ATF parasites are able to actively take up exogenous myristate and form β-oxidation intermediates, demonstrating the existence of this pathway in trypanosomes and suggesting that ATF parasites could in part use fatty acid β-oxidation to satisfy their energy requirements.

## Discussion

A well-established feature of the unicellular, extracellular *T. brucei* parasite is its ability to invade the CNS. Here, we show that while blood is the major site of parasite accumulation on the first peak of parasitemia, fat contains the highest density and total number of parasites later in infection (around 100- to 800-fold more than the brain). Although the reason(s) why parasites accumulate in adipose tissue remain unknown, we clearly show it has dramatic consequences for the parasites. They functionally adapt to the tissue environment by rewiring gene expression, including the possibility of using lipids/fatty acids as a carbon source.

### Possible Advantages to Parasite Accumulation in the Adipose Tissue

Accumulation in the adipose tissue could be due to several non-mutually exclusive reasons that may have provided a selective advantage during evolution: parasites may be less efficiently eliminated by adipose tissue-specific immune response, parasites may grow at a faster rate, parasite differentiation may be delayed, and/or parasite entry in adipose tissue may be more efficient in adipose tissue than in other organs/tissues. Depending on the dynamics of parasite movement to/from adipose tissue, it is possible that fat acts as a source of parasites that can repopulate the blood. This reversible movement between blood and fat could have important implications for (1) the transmission dynamics, since stumpy formation is triggered by a quorum-sensing mechanism (MacGregor et al., 2011), and (2) antigenic variation, if fat, for example, would favor the appearance of new VSG variants that could later go to the blood (Mugnier et al., 2015).

An intriguing question is whether stumpy forms could be directly ingested by a tsetse fly from the subcutaneous fat. Although in our histological analysis we did not find a significant number of parasites in this fat depot, it is possible we missed a preferential skin location. Moreover, we performed this analysis in mice infected by intraperitoneal injection, which bypasses the skin as the first entry point of metacyclic forms. So it remains to be determined whether subcutaneous fat is important for accumulation of metacyclics and/or ATF parasites and how it impacts transmission.

Not all *Trypanosoma* species occupy the same niche in the host. *T. brucei* and *T. evansi* are mainly tissue-invading parasites, while *T. congolense* stays in smaller capillaries and venules of tissues and *T. vivax* remains mainly in circulation (Losos and Ikede, 1972). These differences have been associated with the different swimming properties of each *Trypanosoma* species (Bargul et al., 2016). Previous reports had indirectly suggested that *T. brucei* parasites could be present in adipose tissue (Fernandes et al., 1997, Giroud et al., 2009). Our study demonstrates that *T. brucei* parasites accumulate in high numbers in the fat of rodents. Although mouse is an accepted model to study *T. brucei* infection (Giroud et al., 2009), we cannot exclude the possibility that accumulation in adipose tissue is a result of the selection process that happens when *T. brucei* infects a non-natural host. In the future, it would be interesting to confirm whether fat preference is a common feature of this and other *Trypanosoma* species in their natural hosts and to compare their swimming properties in different tissues/organs.

It is intriguing to note that several pathogens infect adipose tissue. *T. cruzi*, the causative agent of Chagas disease in Latin America, invades adipocytes during acute infections in mice and humans (Ferreira et al., 2011). Also, *Plasmodium berghei*, a causative agent of rodent malaria, sequesters in lungs and fat (Franke-Fayard et al., 2005). *Mycobacterium tuberculosis* infects adipocytes, where it accumulates in intracytoplasmic lipid inclusions and survives in a “dormant” non-replicating state that is insensitive to anti-mycobacterial drugs (Neyrolles et al., 2006). HIV takes advantage of the fat as a viral reservoir during the chronic stage of infection, and persistence on this reservoir is an obstacle for treatment (Chun et al., 2015). It is possible that persistence of *T. brucei* in the fat may also account for some of the treatment failures in humans (Richardson et al., 2016).

### Functional Adaptation to Host Adipose Tissue

A major observation in this work is that 20% of the genes are differentially expressed between ATFs and BSFs, which is comparable with the differences between BSFs and PCFs (around 30%) and between slender and stumpy BSF forms (around 12%) (reviewed in Siegel et al., 2011). Parasites adapt to the fat environment by changing transcript levels of genes involved in metabolism, signaling, cell-cycle control, and RNA binding. Using biochemical assays, we confirmed that ATF can utilize fatty acids, i.e., myristate, and catabolize them via β-oxidation, which could lead to the production of ATP via the TCA cycle and oxidative phosphorylation. Therefore, it seems that parasites can sense and adapt to the adipose environment by rewiring their gene expression, including the ability to use lipid/fatty acid as a carbon source.

The major carbon source of BSF and PCF parasites is glucose and proline, respectively, both of which are readily available nutrients in the host (reviewed in Szöör et al., 2014). Fat is, in its essence, a lipid-rich environment. Therefore, it is possible that fatty acids or some other form of lipid are released from the host adipocytes and are endocytosed or actively transported via a receptor-mediated process by ATF parasites (Vassella et al., 2000). So far, only one receptor has been identified in *T. brucei* as necessary for the import of LDL particles (Coppens et al., 1987). Its transcript levels are not altered in ATF parasites, suggesting either that this protein can be upregulated post- or co-translationally or that lipid/fatty acid import is mediated by yet-uncharacterized transporters.

Consumption/utilization of host’s lipids during a *T. brucei* infection could contribute to the weight loss observed in patients with sleeping sickness, cattle with Nagana, and mice infected with *T. brucei* (Kennedy, 2013, Ranjithkumar et al., 2013). Interestingly, obese mice (*db*^−/−^ knockout mice) infected with *T. brucei* live 3-fold longer than their littermates, suggesting that having more adipose tissue partially protected mice from a *T. brucei* infection (Amole et al., 1985). Because obesity is associated with persistent low-grade chronic inflammation in adipose tissue (Ouchi et al., 2011), it is possible that in obese mice, parasites get more efficiently eliminated (or controlled), thus prolonging the survival of the host.

Most of what is known today about the mechanisms of virulence, persistence, and transmission of *T. brucei* results from studies performed in BSF parasites. The identification of adipose tissue as an additional major reservoir of functionally differentiated *T. brucei* brings a unique perspective to our understanding of this parasite and raises several questions. What is the relative contribution of BSF and ATF parasites for pathogenicity and host metabolic alterations? Could fat act as a source of parasites expressing novel VSGs? What are the implications of such a large reservoir of ATF parasites in terms of transmission? What are the dynamics of parasite entry and exit from fat? Given that the brain is a lipid-rich organ, which is also invaded by *T. brucei*, it is obvious to ask whether these parasites also adapt their gene expression and how this impacts brain-associated pathology. Do ATF parasites induce changes in the host metabolism, providing an advantage to the parasitic infection? Is the immune response of the adipose tissue more permissive to *T. brucei* parasites? Are anti-trypanosome drugs equally efficient at eliminating ATFs and BSFs? In sum, our findings have important consequences for the understanding of parasite biology, disease, and drug treatment efficacy.

## Experimental Procedures

Detailed experimental procedures can be found in Supplemental Experimental Procedures.

### Animal Experiments

Animal experiments were performed according to EU regulations and approved by the Animal Ethics Committee of Instituto de Medicina Molecular (AEC_2011_006_LF_TBrucei_IMM). Tsetse fly infections were performed in compliance with the regulations for biosafety and animal ethics (VPU2014_1) and under approval from the environmental administration of the Flemish government. Unless otherwise indicated, all infections were performed in wild-type male C57BL/6J mice, 6–10 weeks old (Charles River, France), by intraperitoneal injection of 2,000 *T. brucei* AnTat 1.1^E^ 90-13 parasites. For parasite counts, blood samples were taken daily from the tail vein. Organs/tissues of infected mice were collected at days 6 and 28 post-infection unless otherwise stated. Animals were sacrificed by CO_2_ narcosis, blood collected by heart puncture, and mice immediately perfused. Collected organs were snap frozen in liquid nitrogen or fixed in 10% neutral-buffered formalin. In transplants, homogenates as well as 600 μL of blood were transplanted into age- and sex-matched naive mice.

### Histology and Electron and Fluorescence Microscopy

Formalin-fixed organs were immunostained with a non-purified rabbit serum anti-*T. brucei* VSG13 antigen (which crossreacts with many VSGs) and a non-purified rabbit serum anti-*T. brucei* H2A. For TEM, ultra-thin sections (70 nm) were screened in a Hitachi H-7650 microscope. 3D reconstruction of isolated trypanosome was done using the IMOD software package version 4.7.3 for alignment and modeling (Kremer et al., 1996).

For fluorescence analysis, the gonadal depot was stained with LipidTox, fixed in 10% neutral-buffered formalin and embedded in Fluoromount-G. Fluorescence images were taken using a 40× objective in a Zeiss Cell Observer wide-field microscope. For morphometry analysis, isolated parasites were fixed with paraformaldehyde, DAPI stained, and embedded in vectashield. Images were taken using a 63× oil objective with optional optovar magnification (1.6×) in the same wide-field microscope. Parasite measurements were taken essentially as described in Wheeler et al. (2012).

The mitochondrion of isolated parasites was labeled using MitoTracker Green (Invitrogen/Molecular Probes, M-7514) according to the manufacturer’s instructions. Fluorescence and DIC images were acquired using a confocal laser point-scanning microscope (Zeiss LSM 710).

### Parasite Quantification

*T. brucei* 18S rDNA genes were amplified from gDNA of a known mass of tissue and converted into parasite number using a standard curve. For RNA quantification, the ΔΔCt method was used by amplifying *TbZFP3* and mouse *Gapdh* genes from tissue total RNA.

### Parasite Isolation from Tissues

Bloodstream parasites were purified over a DEAE column (Taylor et al., 1974), while ATF parasites were isolated from gonadal fat depot by incubating the depot in MEM or HMI11 at 37°C and 150 rpm agitation for up to 40 min.

### Flow Cytometry

Cell-cycle analysis was performed using propidium-iodide (PI) or Vybrant DyeCycle violet (DCV) in fixed or live cells, respectively. PI staining was done according to Aresta-Branco et al. (2016). For DCV staining, cell suspensions were washed, and 0.5 μL DCV was added per each million isolated parasites and incubated for 10 min at 37°C. PI, DCV, and GFP intensities were measured with BD LSRFortessa cell analyzer.

### RNA-Seq

RNA and cDNA library of both blood and gonadal fat depot from days 4 and 6 of infection, respectively, were prepared as described (Pena et al., 2014), and samples sequenced in an Illumina HiSeq2000 platform. Reads were processed and mapped to the *T. brucei* TREU927 genome. Differential gene expression was analyzed, and genes were considered differentially expressed if they were detected by at least two of the three considered algorithms (p adjusted < 0.01).

### Myristate Metabolic Labeling

To evaluate myristate incorporation and metabolism, the fat isolation protocol was performed in lipid-free minimum essential medium (MEM). Parasites were placed in a vented tube with 1 mL MEM and starved for 30 min at 37°C. Starved parasites were then labeled with 0.4 mg of radiolabeled D_27_-C14:0 pre-coupled with defatted BSA for 1 hr. A total of 450 μL of the cell suspension was washed, snap frozen in liquid nitrogen, and lyophilized in glass vials (pulse sample). The remaining parasites were re-suspended in 500 μL MEM and 100 μL HMI11 for 1 hr at 37°C, and at the end processed as for pulse sample (chase sample). Metabolite extraction, identification, and quantification were conducted as described in Oyola et al. (2012), with the exception that fatty acids were released by acid hydrolysis (200 μL 6M HCl at 110°C for 16 hr).

### Statistical Analysis

Statistical analyses were performed by fitting LME models with mice as random effects unless otherwise indicated. At least three independent experiments were considered in each case and statistical significance was set to α = 0.05 level. Data were analyzed after logarithm transformation.

## Author Contributions

S.T., F.R.-F., T.K.S., and L.M.F. designed the experiments and wrote the paper; S.T., F.R.-F., T.C., F.G., F.A.-B., F.B., A.P., and T.K.S. conducted the experiments; D.P.-N. and S.A.Y. analyzed the RNA-seq data; J.V.D.A. designed and conducted experiments with tsetse flies; R.M.R. conducted the statistical analysis; and S.D. designed experiments.

## Accession Numbers

The accession number for Lister427 culture parasites is ArrayExpress: E-MTAB-1715. Sequence data generated as part of this study have been submitted to the ArrayExpress database (EMBL-EBI) under accession number ArrayExpress: E-MTAB-4061.

## Supplemental Information

Document S1. Supplemental Experimental Procedures, Figures S1–S7, and Table S1

Table S2. Genes Differentially Expressed between Parasites from Adipose Tissue and Blood Related to Figure 5.

Table S3. Classes of Transcripts Differentially Expressed between Parasites in Adipose Tissue and Blood Related to Figure 5.

Movie S1. 3D-Reconstruction Model of Part of an ATF Parasite Related to Figure 4. Model constructed from hand-drawn contours marking the boundaries of cellular components in a tomogram of a trypanosome isolated from mouse gonadal adipose. The video is projected using Image J, and various sub-cellular organelles are highlighted through subjectively attributed colors (plasma membrane in yellow, endocytic vesicles in dark blue, glycosomes in pink, nucleus in white, and mitochondrion in green).

Document S2. Article plus Supplemental Information
