# Supplementary material for: Trypanosoma brucei Parasites Occupy and Functionally Adapt to the Adipose Tissue in Mice
Source: Cell Host Microbe. 2016 Jun 8;19(6):837–48. doi: 10.1016/j.chom.2016.05.002 (PMC4906371; doi:10.1016/j.chom.2016.05.002)
Supplement: Document S1. Supplemental Experimental Procedures, Figures S1–S7, and Table S1 [file mmc1.pdf]

Cell Host & Microbe, Volume *19*

Supplemental Information

Trypanosoma brucei Parasites Occupy and Functionally Adapt to the Adipose Tissue in Mice

Sandra Trindade, Filipa Rijo-Ferreira, Tânia Carvalho, Daniel Pinto-Neves, Fabien Guegan, Francisco Aresta-Branco, Fabio Bento, Simon A. Young, Andreia Pinto, Jan Van Den Abbeele, Ruy M. Ribeiro, Sérgio Dias, Terry K. Smith, and Luisa M. Figueiredo

A

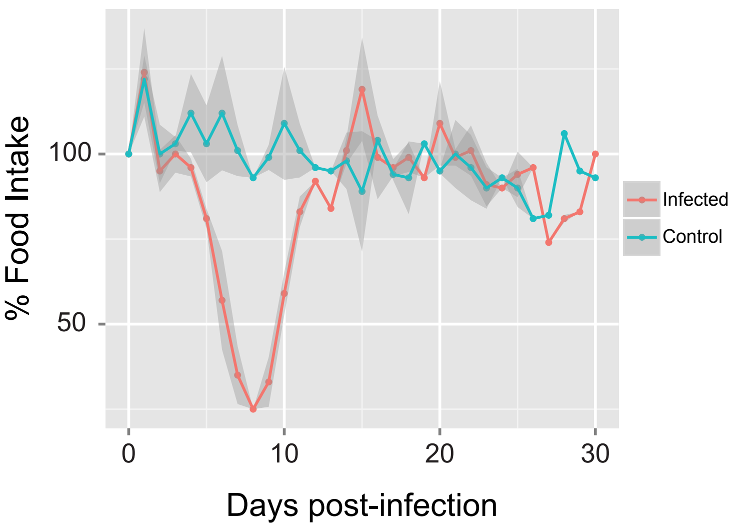

B

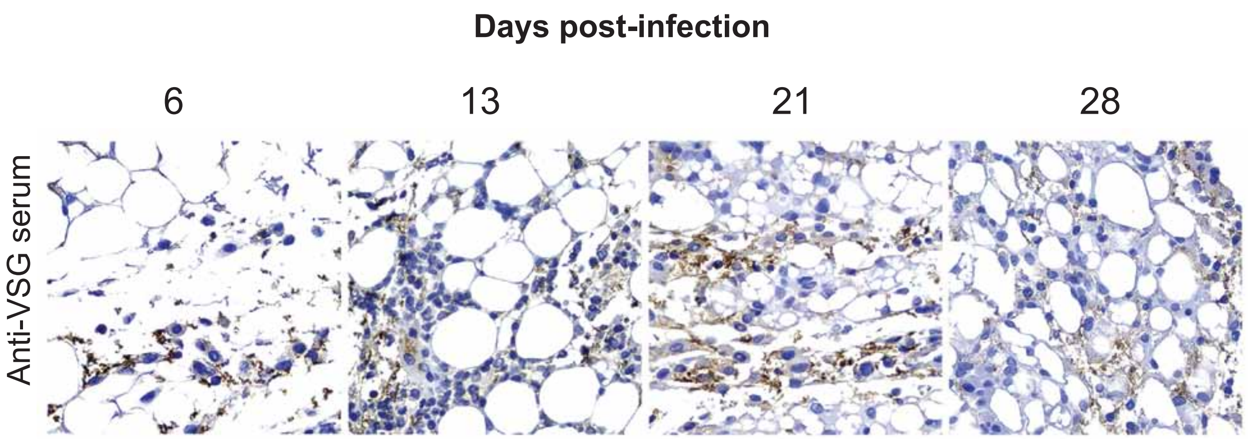

Figure S1. Related to Figure 1 and Figure 2. Clinical and histology details during *T. brucei* infection.

C57BL/6J mice were injected i.p. with 2000 AnTat1.1E parasites.
(A) Variation of food intake during infection. Animals (n = 15 per group) were group housed and food intake was measured daily by weighting the food and dividing by the number of mice per cage (n = 4 per condition). Light grey shaded area represents SEM.
(B) Representative brightfield micrographs of gonadal fat depots at different days post-infection, assessed by immunohistochemistry with anti-VSG antibody (parasites appear in brown). Original magnification, 400x.

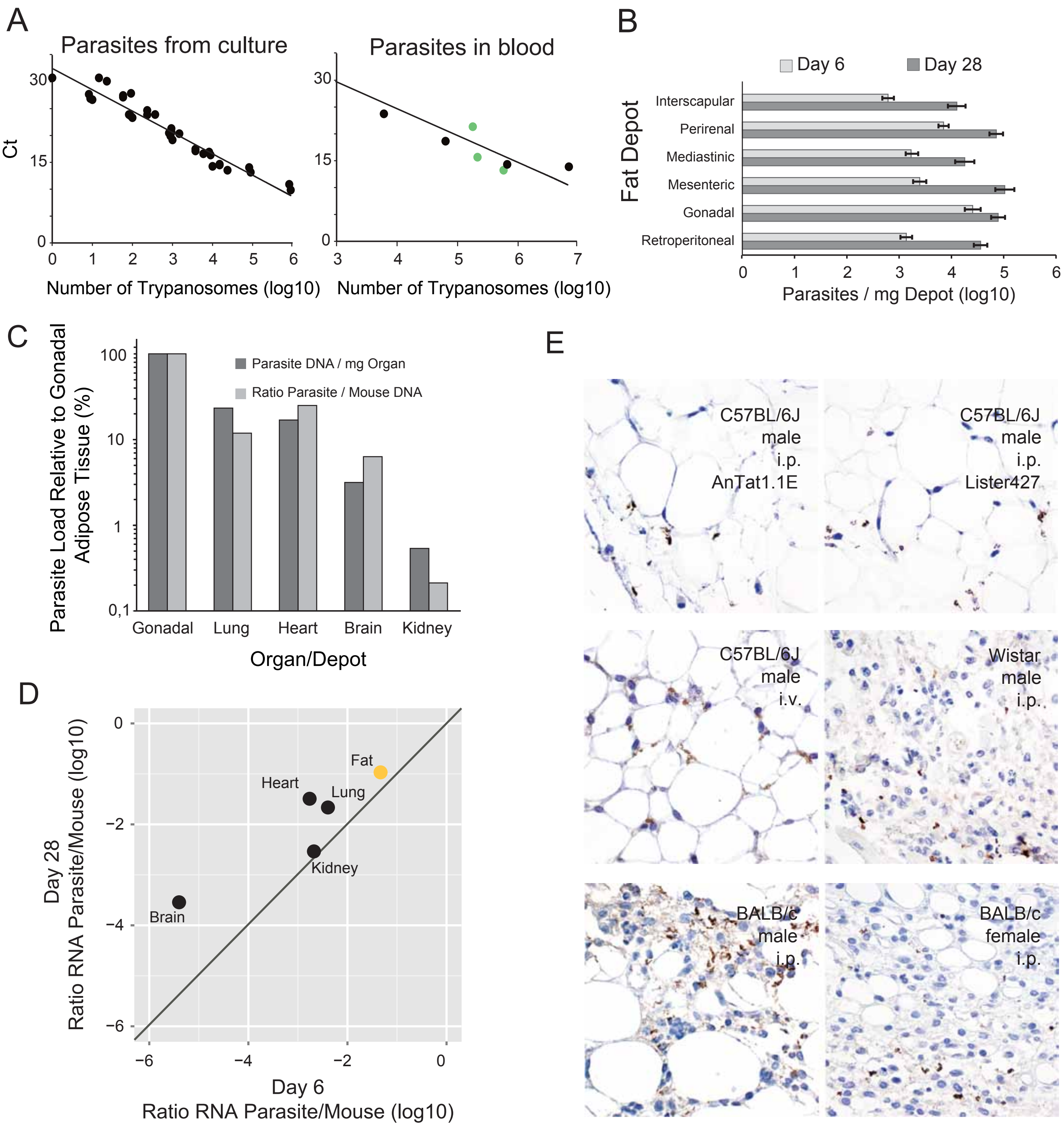

A
Parasites from culture
Parasites in blood
Ct
Number of Trypanosomes (log10)
Number of Trypanosomes (log10)
B
Day 6
Day 28
Fat Depot
Interscapular
Perirenal
Mediastinic
Mesenteric
Gonadal
Retroperitoneal
Parasites / mg Depot (log10)
C
Parasite Load Relative to Gonadal Adipose Tissue (%)
Parasite DNA / mg Organ
Ratio Parasite / Mouse DNA
Gonadal
Lung
Heart
Brain
Kidney
Organ/Depot
D
Day 28 Ratio RNA Parasite/Mouse (log10)
Fat
Heart
Lung
Kidney
Brain
Day 6
Ratio RNA Parasite/Mouse (log10)
E
C57BL/6J male i.p. AnTat1.1E
C57BL/6J male i.p. Lister427
C57BL/6J male i.v.
Wistar male i.p.
BALB/c male i.p.
BALB/c female i.p.

Figure S2. Related to Figure 1 and 2. Further validation of preferential accumulation of parasites in adipose tissue.

(A) Calibration curve obtained from four independent *in vitro* cultures of known cell density. gDNA was extracted, serially diluted and amplified by quantitative PCR using *T. brucei* 18S rDNA primers. The goodness of fit of the linear regression is $R^2 = 0.933$. Calibration curve obtained from blood from infected mice (n = 3) (green dots) and from a culture of parasites diluted in blood from naive mice (n = 1) (black dots). The goodness of fit of the linear regression is $R^2 = 0.925$.
(B) Parasite density in six fat depots on day 6 (n = 6-12) and 28 (n = 3-6) post-infection determined by qPCR of gDNA. For each depot, significant differences were found between days 6 and 28 post infection (Student's unpaired t test, $P < 0.05$).
(C) Two different gDNA qPCR methods were compared on day 28 post-infection: i. number of parasites per mg of organ (n = 3 - 9) and ii. ratio between *T. brucei and* mouse 18S gDNA (n = 3 - 9). Both methods show a similar parasite density in different tissues (LME, P = 0.72).
(D) Parasite density on day 6 and 28 post-infection determined by qRT-PCR of RNA. Transcripts of the parasite *TbZFP3* gene were normalized to the mouse *Gapdh*. Each point represents the geometric mean of the parasite density on day 6 (n = 3 - 4) and on day 28 post-infection (n = 3 - 5). RNA quantification validates the conclusions taken from gDNA qPCR: the adipose tissue is the major parasite reservoir (LME, P = 0.0006). The relative contribution of other organs is similar to what was measured by gDNA, except for brain, in which parasite density was lower than expected. Perhaps *TbZFP3* is downregulated in this organ.
(E) Representative brightfield micrographs of *T. brucei* in gonadal adipose tissue in different models of infection, assessed by immunohistochemistry with anti-VSG antibody (parasites appear in brown). Original magnification, 400x.

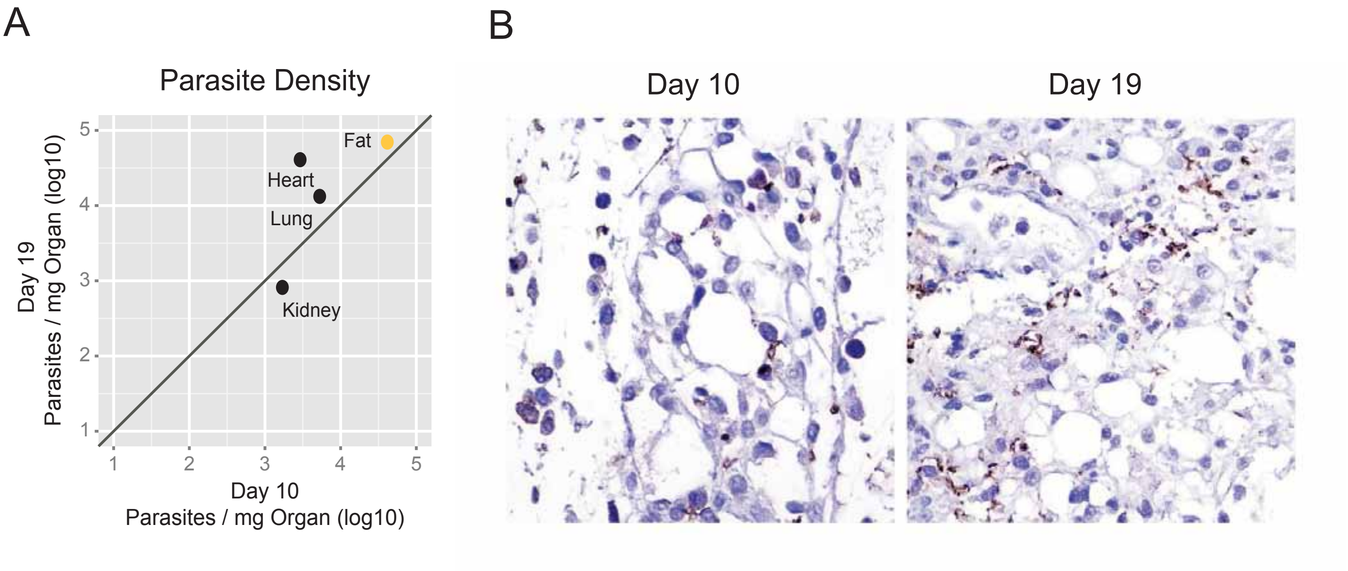

Figure S3. Related to Figure 2. Parasites accumulate in fat when infection is initiated by tsetse bite.

(A) Parasite density in multiple organs in mice naturally infected by the bite of a tsetse fly. Mice were sacrificed at the first and second peaks of parasitemia (10 and 19 days post-infection respectively) and parasite density determined by amplification of gDNA, as described in Figure 2. Each point represents the geometric mean of the parasite density at day 10 (n = 4) and at day 19 (n = 8).
(B) Representative brightfield micrographs of gonadal fat depot immunostained with anti-VSG serum, 10 and 19 days post-infection by tsetse bite. Original magnification, 40x.

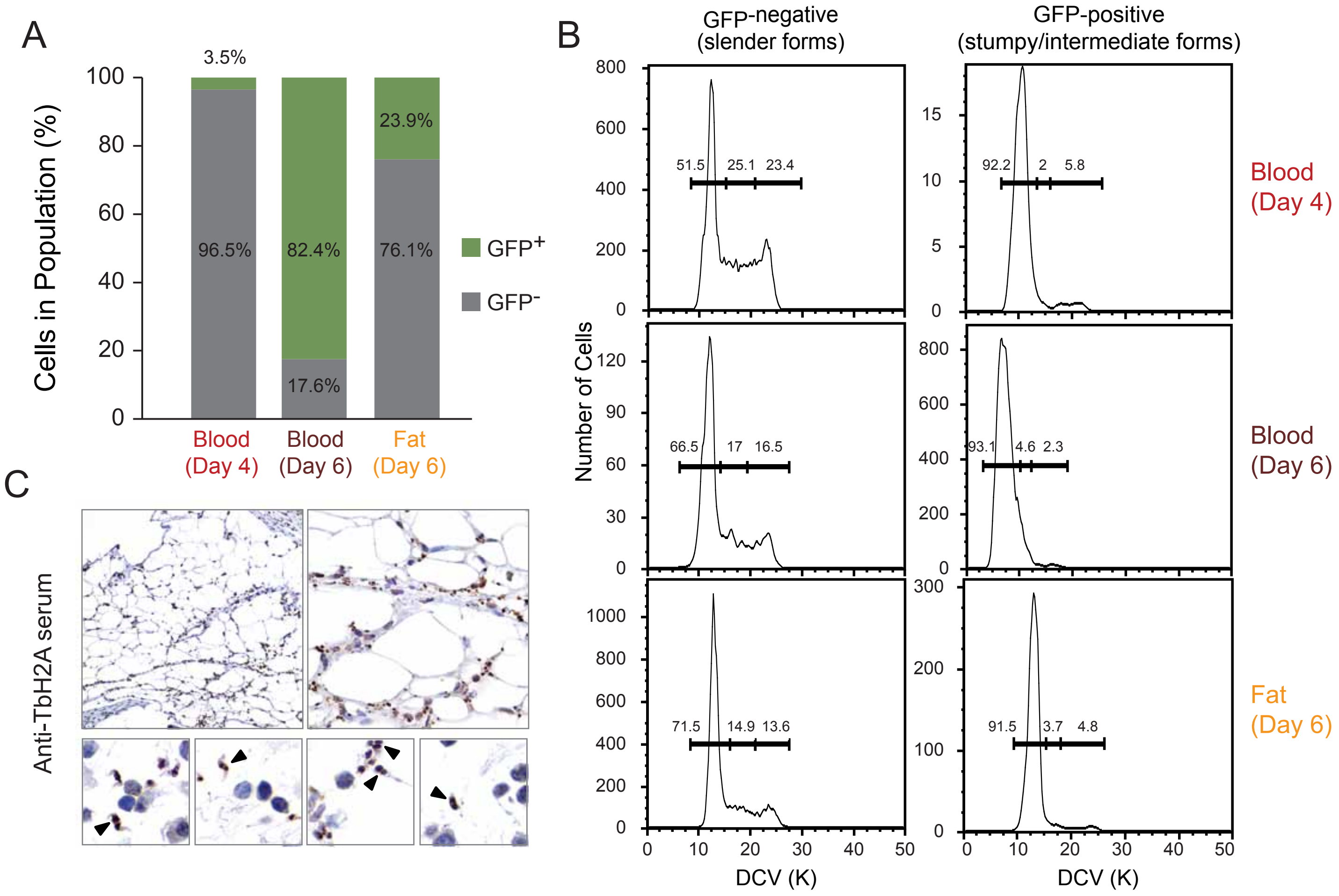

Figure S4. Related to Figure 3. Complementary methods to confirm presence of both replicative and cell-cycle arrested parasites in fat on day 6 post-infection.

(A) GFP Expression and (B) Cell cycle analysis in parasites isolated from blood and adipose tissue, 4 or 6 days post-infection, assayed by FACS. A *GFP::PAD1*$_{3UTR}$ *T. brucei* reporter cell-line was stained with DyeCycle Violet. The histograms represent the distribution and the percentage of parasites in each cell cycle stage. DyeCycle Violet staining validates the conclusions taken from propidium iodide staining analysis.

(C) Representative brightfield micrographs of gonadal fat depots on day 6 post-infection, assessed by immunohistochemistry with anti-*T. brucei* H2A rabbit serum (parasites appear in brown). Original magnification, 20x, 40x and 100x. Arrowheads indicate parasites undergoing nuclear division.

A

GFP expression	Tissue	Length (µm)	Width (µm)
GFP-negative (slenders)	Fat	24.57 ± 2.99	2.12± 0.26
	Blood	24.39 ± 2.50	2.15 ± 0.26
GFP-positive (stumpy/interm.)	Fat	21.32 ± 2.73	2.29 ± 0.31
	Blood	18.43 ± 1.81	3.11 ± 0.38

n= 100, Numbers are mean values with ± standard deviations .

B

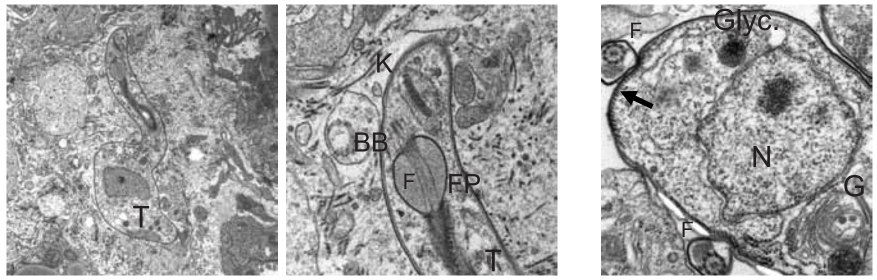

C

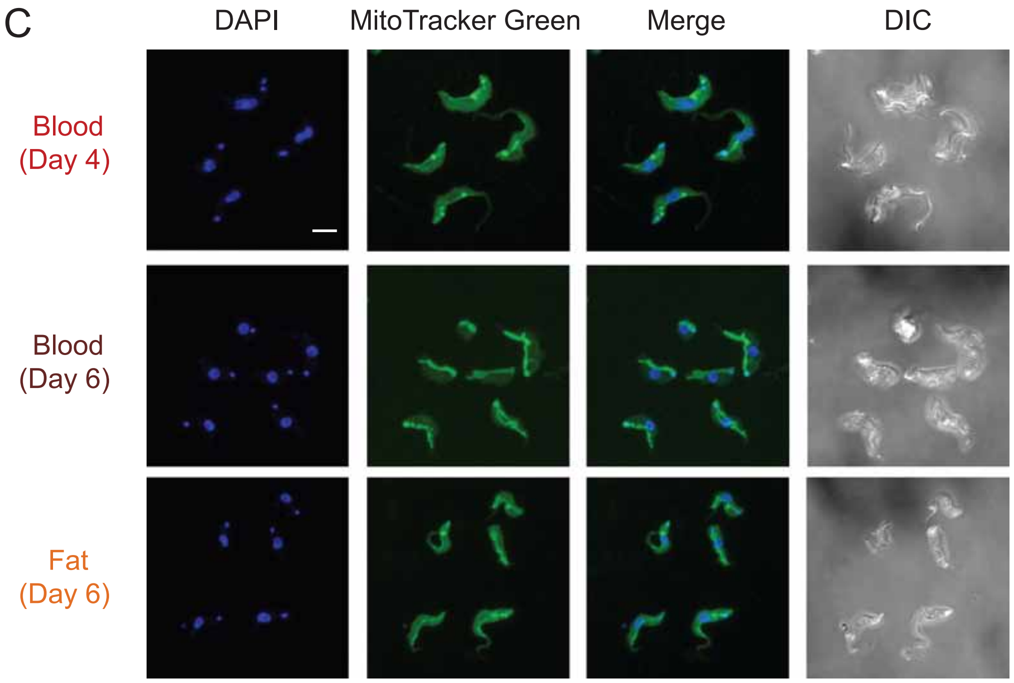

Figure S5. Related to Figure 4. Subcellular organization of parasites from adipose tissue.

(A) Length and width mean values of GFP-negative (slender) and GFP-positive (stumpy/intermediate) parasites isolated from blood or fat.
(B) Transmission electron micrographs of parasites in gonadal adipose tissue (day 28 post-infection).
T, trypanosome; K, kinetoplast; BB, basal body; F, flagellum; FP, flagellar pocket; N, nucleus; Glyc, Glycosomes; Arrow: subpellicular microtubules. Scale bars represent 2 and 0,5 µm in the left and right panels, respectively.
(C) MitoTracker Green, which stains in live cells the mitochondrion membrane, regardless of its membrane potential, was used to assess mitochondrion morphology. DNA was stained by DAPI and images were captured under a confocal microscope. Bloodstream form parasites from day 4 of infection showed a punctate pattern typical of mitochondrion in slender parasites, while on day 6, mitochondrion displayed a tubular structure with a few branches in what appeared to be stumpy forms. The mitochondrion of adipose tissue forms present a tubular structure, but with fewer branches and thinner. All panels are shown with the same magnification. Scale bar represents 5 µm.

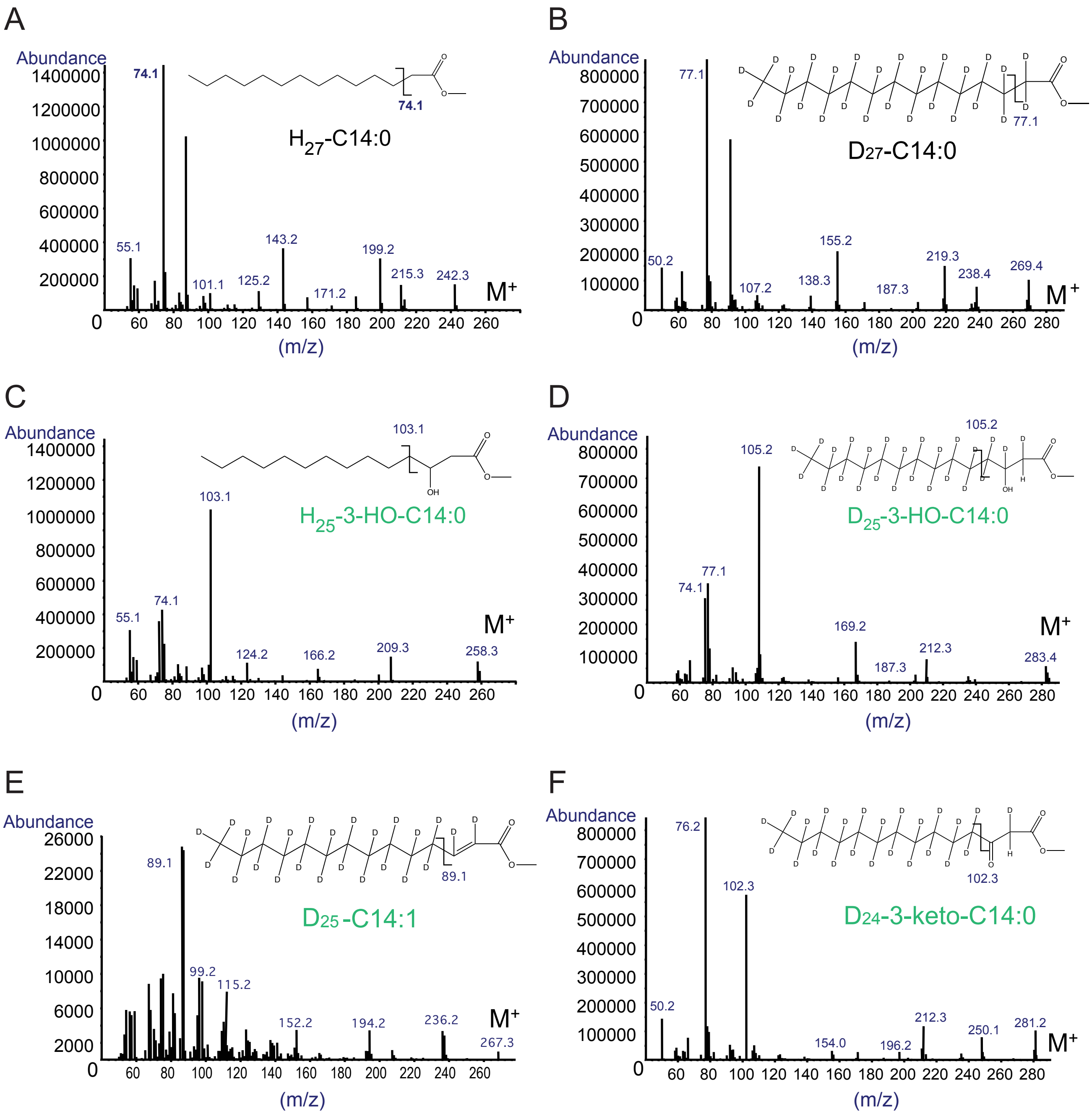

Figure S6. Related to Figure 6. Lipid metabolites identified by GC-MS.

All panels show the structure and fragmentation pattern of methyl ester derivatives of:

(A) myristic acid (C14:0)

(B) fully deuterated-myristic acid (D_{27}-C14:0)

(C) 3-hydroxy- myristic acid (3-HO-C14:0)

(D) 3-hydroxy-deuterated- myristic acid (D_{24}-3-HO-C14:0)

(E) deuterated-myristoleic acid (D_{25}-C14:1)

(F) 3-keto-deuterated- myristic acid (D_{24}-3-keto-C14:0)

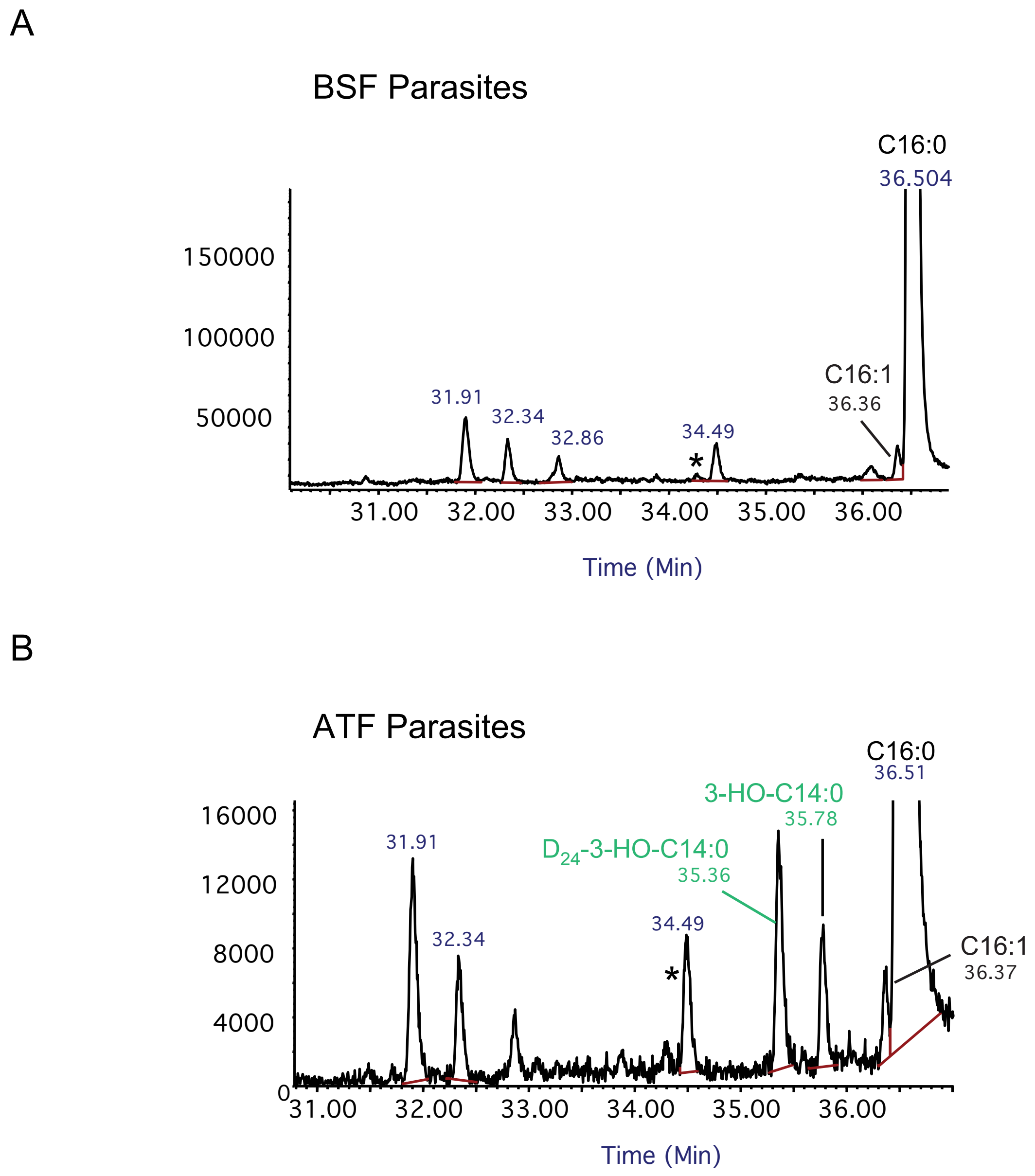

Figure S7. Related to Figure 6. Only ATF parasites produce hydroxyl-fatty acids as part of β-oxidation catabolism of fatty acids.
FAME analysis by GC-MS of D_{27}-Mys labeled and subsequently chased bloodstream (A) and adipose tissue (B) forms. Trace 31-37 minutes showing positions of (3-HO-C14:0) and (D_{24}-3-HO-C14:0) in adipose tissue forms only.

Supplemental Tables and Movies

Table S1, related to Figure 5. Mapping information of RNA-Seq reads in samples from blood and adipose tissue.

Sample	Total # reads	# of reads mapped to mouse genome	% of mouse reads in dataset	# of reads mapped to *T. brucei* genome	% of *T. brucei* reads in dataset
Blood-A	20 491 498	282 806	1,4%	16 004 171	78,1%
Blood-B	22 485 284	255 967	1,1%	18 418 295	81,9%
Fat-A	36 739 561	32 564 158	88,6%	3 396 819	9,2%
Fat-B	37 741 627	36 837 101	97,6%	415 936	1,1%
Fat-C	45 755 070	42 030 335	91,9%	3 311 276	7,2%

Table S2, related to Figure 5. Genes differentially expressed between parasites from adipose tissue and blood. (EXCEL file)

Table S3, related to Figure 5. Classes of transcripts differentially expressed between parasites in adipose tissue and blood. (EXCEL file)

Movie S1, related to Figure 4. Model constructed from hand-drawn contours marking the boundaries of cellular components in a tomogram of a trypanosome isolated from mouse gonadal adipose. The video is projected using Image J and various sub-cellular organelles are highlighted through subjectively attributed colors (plasma membrane in yellow; glycosomes in pink; nucleus in white; mitochondrion in green).

Supplemental Experimental Procedures

Ethical Statements

Animal experiments were performed according to EU regulations and approved by the Animal Ethics Committee of Instituto de Medicina Molecular (iMM) (AEC_2011_006_LF_TBrucei_IMM). The animal facility of iMM complies with the Portuguese law for the use of laboratory animals (Decreto-Lei 113/2013); and follows the European Directive 2010/63/EU and the FELASA (Federation of European Laboratory Animal Science Associations) guidelines and recommendations concerning laboratory animal welfare. The tsetse fly mediated *T. brucei* infection work was performed in compliance with the regulations for biosafety and animal ethics (VPU2014_1) and under approval from the Environmental administration of the Flemish government.

Cell Lines

The majority of the infections described in this manuscript were performed using *T. brucei* AnTat 1.1E, a pleomorphic clone derived from an EATRO1125 clone. AnTat 1.1E 90-13 is a transgenic cell-line encoding the tetracyclin repressor and T7 RNA polymerase (Engstler and Boshart, 2004). Tsetse infections were performed with *T. brucei* AnTAR1 strain. We also used *T. brucei* Lister 427, a monomorphic strain derived from antigenic type MiTat 1.2, clone 221a (Johnson and Cross, 1979). The stumpy reporter cell line *GFP::PAD1*$_{utr}$ derives from AnTat 1.1E 90-13 in which the green fluorescent protein, GFP, is coupled to PAD1 3'UTR. A nuclear localization signal (NLS) targets GFP protein into the nucleus. Prior to infection, *T. brucei* cryostabilates were thawed and parasite mobility was checked under an optic microscope.

Mice Infections

Mice were group-housed in filter-top cages and maintained in a Specific-Pathogen-Free barrier facility. The facility has standard laboratory conditions: 21 to 22°C ambient temperature and a 12 h light/12 h dark cycle. Chow and water were available *ad libitum*. Unless otherwise stated, all infections were performed in wild-type male C57BL/6J mice, 6-10 week old (Charles River, France), by intraperitoneally (i.p.) infection of 2000 *T. brucei* AnTat 1.1E 90-13 parasites.

To test if the presence of parasites in fat was dependent on the model, we performed the following variations in the infection protocol: a) male C57BL/6J mice were infected with a more virulent strain, the *T. b. brucei* strain Lister 427; b) male C57BL/6J mice were infected intravenously (i.v.) in the tail vein; c) a different strain of mice, BALB/c, and 7 week old Wistar rats (Charles River, France) were also infected (the latter with 4000 parasites); d) female C57BL/6J mice and finally e) male C57BL/6J mice naturally infected by tsetse bite. For this latter protocol, freshly emerged *Glossina morsitans morsitans* flies were fed their first blood meal on a *T. brucei* AnTAR1 infected mice at the peak of parasitemia. Subsequently, flies were maintained on commercially available defibrinated horse blood through *in vitro* membrane feeding. Thirty days after the infective blood meal, individual flies were evaluated for the presence of metacyclic trypanosomes in their salivary glands by salivation on pre-warmed (37°C) glass slides. To initiate a natural infection, one individual tsetse fly with a mature salivary gland infection was allowed to probe and feed per mouse.

Clinical Parameters and Organ Collection

All measurements in mice were made between 17:00 and 18:00. For parasite counts, blood samples were taken daily from the tail vein, and parasitemia was determined by manual counting using a Neubauer chamber. Organs/tissues of infected C57BL/6J mice (male and female) were collected at days 6, 13, 21 and 28 post-infection; for the infection with *T. b. brucei* strain Lister 427, organs were collected once parasitemia reached $1x10^8$ parasites/mL; for BALB/c mice, at day 6 post-infection; and for Wistar rats and C57BL/6J mice i.v. infections, at days 6, 13 and 20 post-infection. Animals were sacrificed by CO_2 narcosis, blood was collected by cardiac puncture and perfusion was performed to eliminate blood and parasites from circulation. Briefly, mice were perfused transcardially with pre-warmed heparinized saline (50 mL 1X phosphate buffered saline (1X PBS) with 250 µL of 5000 I.U./mL heparine per animal) using a peristaltic pump, ranging its speed from 2 mL/min to 8 mL/min. Organs were then collected and either used immediately for parasites isolation, snap frozen in liquid nitrogen for molecular analysis, or fixed in 10% neutral-buffered formalin for histopathology.

Transplantations

Donor mice were sacrificed with CO_2 narcosis (at days 21 and 28 post-infection) and the gonadal fat depot, brain, heart, and 600 µL of blood were harvested. Organs were manually homogenized through a 70 µm mesh into 1X PBS. Cell suspension was centrifuged at 1000 g for 10 minutes and resuspended in 800 µL of HMI11. Tissue lysates were injected intraperitoneally in naïve mice. Blood was diluted in 1X PBS and centrifuged for 5 minutes at 2800 g. Cell pellet was diluted in 800 µL of 1X PBS and injected intraperitoneally in naïve mice.

Histology

Formalin-fixed organs were embedded in paraffin and 3µm sections were stained with hematoxylin and eosin (H&E). For immunohistochemistry, 3µm sections were stained for VSG using non-purified rabbit sera anti-*T. brucei* VSG13 antigen (cross-reactive with most VSGs via the C-terminal domain) or anti-*T. brucei* H2A (generated against a recombinant protein) (kind gift of Christian Janzen), diluted 1:5000 and 1:3000, respectively. Briefly, antigen heat-retrieval was performed in a microwave oven (800 w) for 15 minutes with pH 9 Sodium Citrate buffer (Leica Biosystems, MO, USA). Incubation with ENVISION kit (Peroxidase/DAB detection system, Dako Corp, Santa Barbara, CA) was followed by Mayer's hemalumen counterstaining. No staining was observed in the negative control (without primary antibody). Tissue sections were examined by a pathologist (TC), blinded to experimental groups, in a Leica DM2500 microscope coupled to a Leica MC170 HD microscope camera.

For transmission electron microscopy, gonadal fat depot from infected mice (days 6 and 28 post-infection) was collected and fixed for three hours at 4ºC in 0.1 M cacodylate buffer, pH 7.4, containing 2.5% (v/v) glutaraldehyde. After staining for 1 hour with 1% (w/v) osmium tetroxide and 30 minutes with 1% (w/v) uranyl acetate, samples were dehydrated in an ethanol gradient (70-95-100%), transferred to propylene oxide and embedded in EPON™ resin. Semi-thin sections (300-400 nm) were stained with toluidine blue for light microscopy evaluation. Ultra-thin sections (70 nm) were collected in copper slot grids and stained with 2% uranyl acetate and lead citrate (Reynolds recipe). Grids were screened in a Hitachi H-7650 transmission electron microscope at 100 kV acceleration.

For 3D reconstruction of isolated trypanosomes, parasites isolated from gonadal fat depot were centrifuged at 5000 rpm and processed as described above for whole tissue. After embedding, approximately 26 serial ultra-thin sections (70 nm) were collected for each individual parasite. Grids of seven parasites were screened in a Hitachi H-7650 transmission electron microscope at 100 kV acceleration, serial section alignment was achieved using the IMOD software package version 4.7.3 for alignment and modeling (Kremer et al., 1996). ,Videos were projected using ImageJ 4.47v.

Microscopy analysis

Parasites from blood were isolated using a DEAE column (Taylor et al., 1974) and parasites from fat were isolated from gonadal fat depot by incubating the depot in HMI11 for up to 40 minutes and then purified from tissue debris over a DEAE column. For morphometric analysis, $1x10^6$ parasites were settled for 15 minutes to a pre-coated dish with 10% poly-Lysine, fixed with 2% paraformaldehyde for 10 minutes at room temperature, washed with 1X PBS and stained with diamidino-2-phenylindole (DAPI) (5 µg/mL in 1X PBS). Vectashield solution was used to mount the dish. Fluorescence and Phase Contrast images were acquired using Zeiss Cell Observer wide-field microscope. Parasite length was taken essentially as described in (Wheeler et al., 2012). Parasite width was manually scored in the nuclear region of the cell body.

For mitochondrial staining, isolated parasites were incubated in HMI-11 with 700 µM MitoTracker Green for 30 minutes at 37˚C. Excess of MitoTracker stain was removed and cells were chased in HMI-11 medium for 40 minutes at 37˚C. After washing with 1X PBS, cells were fixed and handled as above. Fluorescence and DIC images were acquired using a confocal Laser Point-Scanning Microscope (Zeiss LSM 710).

For tissue fluorescence analysis, the gonadal depot was stained with LipidTox (1:200 v/v in 1X PBS) for 30 minutes at 4ºc with agitation and then fixed in 10% neutral-buffered formalin, Sigma, and washed twice in 1 mL 1X PBS for 30 minutes at 37ºC with 150 rpm horizontal agitation. After, fat samples were embedded and mounted in Fluoromount-G. Fluorescence images were taken using a 40X objective in a Zeiss Cell Observer WF Microscope.

Parasite quantification in organs and tissues

Collected organs/tissues were snap frozen in liquid nitrogen. Genomic DNA (gDNA) was extracted from tissues using NZY tissue gDNA isolation kit (NZYTech, Portugal). The primers used for amplification of 18S rDNA gene of *T. brucei* were 5'–ACGGAATGGCACCACAAGAC–3' and 5'–GTCCGTTGACGGAATCAACC–3' and for the mouse 18S rDNA gene were 5'–TCGAGGCCCTGTAATTGGAA–3' and 5'–CTTTAATATACGCTATTGGAGCTGGAA–3'. By qPCR, the amount of *T. brucei* 18S rDNA present per milligram of organ/tissue were measured and converted into number of parasites using a calibration curve. The curve used to calculate parasite density in most solid organs/tissues was obtained by quantifying *T. brucei* 18S rDNA from serial dilutions of four independent cultures of AnTat1.1E 90-13 of known cell densities. Because PCR amplification from blood genomic DNA is sensitive to hemoglobin, we made a calibration curve specifically for blood samples, which was obtained by quantifying *T. brucei* 18S rDNA from blood from three infected mice of known parasitemia and from a known number of culture parasites diluted in blood. Parasite density in organs/tissues was calculated by dividing the number of parasites by the weight of organ/tissue used for qPCR, while total parasite load was calculated by multiplying the number of parasites/mg by the weight of organ/tissue at the corresponding day of infection. Blood density is about 1.05 kg/L, which means that 1 mL of blood is roughly equivalent 1050 mg. This conversion was taken into account to convert the number of parasites per mL of blood into number of parasites per mg of blood.

Parasite quantification from gDNA was also undertaken using ΔΔCt method. Here *T. brucei* and Mouse 18S rDNA genes were quantified from total genomic DNA. The ratio of the two genes provided a relative parasite density.

Given that trypanosomal gDNA can remain in circulation for up to 14 days, RNA qPCR was also used to quantify parasite load in tissues by calculating the ratio of *T. brucei TbZFP3* transcript to mouse *Gapdh* transcript. RNA was extracted using TRIzol or TRIzol LS reagents (Life Technologies) and cDNA prepared with TaqMan® Reverse Transcription Reagents (Invitrogen). The primers used for amplification of *TbZFP3* gene of *T. brucei* were 5'–CAGGGGAAACGCAAAACTAA–3' and 5'–TGTCACCCCAACTGCATTCT–3' and for mouse *Gapdh* gene were 5'–CAAGGAGTAAGAAACCCTGGACC–3' and 5'– CGAGTTGGGATAGGGCCTCT–3'. Quantitative PCR (qPCR) was performed on an ABI StepOnePlus real-time PCR machine and data was analyzed with the ABI StepOne software.

GFP Expression and Cell Cycle FACS Analysis

To quantify and characterize stumpy population in blood and fat, blood was collected by heart puncture from mice infected for 4 or 6 days, while gonadal fat depot was collected only on day 6 post infection (day 4 yield is too low to obtain enough isolated parasites). Parasites from blood were isolated using a DEAE column (Taylor et al., 1974) and parasites from fat were isolated from gonadal depot by incubating the depot in HMI11 for up to 40 minutes (hemocytometer was used to score the number of released parasites with time). GFP-expression in isolated parasites was analyzed on a BD LSRFortessa™ cell analyzer and data processed using FlowJo.

Cell-cycle analysis was performed using propidium iodide or Vybrant DyeCycle violet. For propidium-iodide, cells were first washed in ice-cold 1X PBS and then fixed with ice-cold 100% ethanol. After a washing step in 1X PBS, 0.5 μL of propidium iodide and 0.5 μL of RNaseA were added per each million of isolated parasites and incubated for 30 minutes at 37ºC. Vybrant DyeCycle violet staining was used in live cells. Essentially, cell suspensions from both blood and fat were washed once in 1X Trypanosome Dilution Buffer (1X TDB). 0.5 μL of DyeCycle violet was added per each million of isolated parasites and incubated for 10 minutes at 37ºC. Intensities were measured with BD LSRFortessa™ cell analyzer.

RNA-Sequencing

Blood from mice infected for 4 days was collected by heart puncture, parasites were purified over a DEAE column (Taylor et al., 1974) and total RNA extracted using TRIzol LS reagent. Mice infected for 6 days were sacrificed and perfused as explained above. Gonadal fat depot was collected and immediately lysed using TRIzol reagent. RNA and cDNA library preparation essentially followed the protocol in (Pena et al., 2014). Samples were sequenced in an Illumina HiSeq2000 platform (EMBL and BGI). Sequenced reads were 100bp paired-end for day 6 fat samples A and B, and 52bp single-end for the remaining samples. Paired-end reads were preprocessed by discarding the second read of each mate-pair, and trimming the first read to 52bp. Sequenced read quality was assessed using the FASTQC quality

control tool (http://www.bioinformatics.babraham.ac.uk/projects/fastqc/). The SolexaQA suite of programs (http://www.biomedcentral.com/1471-2105/11/485) (Cox et al., 2010) was used to trim raw reads to their longest contiguous segment above a PHRED quality threshold of 28, and reads smaller than 25 nucleotides long were discarded. Reads were mapped to the *T. brucei* TREU927 (TriTrypDB 8.0) and *Mus musculus* (GRCm38) genomes using Tophat 2 (REF: http://www.genomebiology.com /2013/14/4/R36/abstract) (Kim et al., 2013) with library type unstranded and the total number of reads mapped to each organism was assessed (Table S1). The number of reads mapped to each CDS was quantified in the R software environment using the function summarizeOverlaps from the GenomicAlignments (1.2.2). We found that approximately 0.1% of the reads could not be unambiguously assigned to the mouse or Trypanosome genome. To avoid a possible contamination of the Trypanosome differential expression results with reads originating from mouse RNA, all reads that mapped, uniquely or not, to the mouse genome were discarded for further analysis. Differential gene expression was analysed using DESeq2 (v1.6.3) (Anders and Huber, 2010), edgeR (v3.8.6) (Robinson et al., 2010) and baySeq (2.0.50) (Hardcastle and Kelly, 2010) from Bioconductor (v3.0). Genes were considered differentially expressed if they were detected by at least two of these three algorithms (P adjusted < 0.01). The ArrayExpress accession number for Lister427 culture parasites is E-MTAB-1715. Sequence data generated as part of this study have been submitted to the ArrayExpress database (EMBL-EBI) under accession number E-MTAB-4061.

Myristate Metabolic Labeling

To evaluate myristate incorporation and degradation by *T. brucei*, blood parasites were isolated using a DEAE column (Taylor et al., 1974) and fat parasites were isolated from gonadal depot by incubating it in lipid free 1X Minimum Essential Medium (MEM) for up to 40 minutes (hemocytometer was used to score the number of released parasites with time). MEM was chosen instead of HMI11 to ensure no external stimulus would be given to the parasites. After isolation, parasites were washed in MEM, resuspended in 1 mL of the same medium, placed in an open/vented tube and starved of fatty acids for 30 minutes while in a water bath at 37ºC with 100 rpm horizontal agitation. To the starved parasites, 200 μL of labeled myristate (D_{27}-myristic acid, CDN) pre-coupled with fatty acids free BSA were added. This solution was obtained by adding $2x10^{-3}$g of labeled myristate dissolved in 40 μL 100% ethanol (that was left at room temperature for 20 minutes) to $2x10^{-3}$g of fatty acids free BSA dissolved in 960 μL of MEM (that was left at 60ºC for 20 minutes), after a short spin the supernatant was ready for use. Parasites were once again incubated in a water bath at 37ºC with 100 rpm horizontal agitation for a one hour period (pulse). After pulse, 450 μL of the cell suspension were washed in 1 mL 1X TDB, snap frozen in liquid nitrogen and lyophilized in glass vials. The remaining parasites were centrifuged at 800 g for 5 minutes and re-suspended in 500 μL of MEM and 100 μL of HMI11. This cell suspension was incubated in a water bath at 37ºC with 100 rpm horizontal agitation for one hour (chase). Just as after pulse, cells were washed in 1X TDB and snap frozen in liquid nitrogen and lyophilized in glass vials. After, samples were spiked with an internal standard fatty acid C17:0, Sigma (20 μL of 1 mM) and dried under nitrogen. Acid hydrolysis was conducted using constant boiling HCl 6 M (200 μL) vortexing/sonication followed by incubation for 16 hours at 110 ºC. After cooling, the samples were evaporated to dryness in a speedvac concentrator and dried twice more from 200 μL of methanol:water (1:1). The protonated fatty acids were extracted by partitioning between 500 μL of HCl 20 mM and 500 μL of ether, the aqueous phase was re-extracted with 500 μL of fresh ether and the combined ether phases were dried under nitrogen in a glass vial. These fatty acids were then converted to methyl esters (FAME), by adding diazomethane (3 x 20 μL aliquots) to the dried residue, while on ice. After 30 minutes samples were allowed to warm to room temperature and left to evaporate to dryness in a fume hood. The FAME products were dissolved in 10-20 μL of dichloromethane and 1-2 μL analyzed by GC-MS on a Agilent Technologies (GC-6890N, MS detector-5973) with a ZB-5 column (30 M x 25 mm x 25 mm, Phenomenex), with a temperature program of at 70 ºC for 10 minutes followed by a gradient to 220 ºC at 5 ºC /minute and held at 220ºC for a further 15 minutes. Mass spectra were acquired from 50-550 amu. The FAME and the β-oxidation metabolites were identified based upon retention time and their fragmentation spectra.

Statistical Analysis

Statistical analyses were performed in the free software R: http://www.r-project.org. Statistically significant variation of body weight, food intake between infected and control mice and parasite density in multiple fat depots were determined by using Student's unpaired t test. Statistically significant variation of parasite morphology was determined by Wilcoxon rank sum test. To define the uniformity of the outcome of the two different parasites quantification methods we fitted a Linear Mixed Effects Model

(LME), using the nlme package in R, considering mice as random factors. The bias in parasite density and parasite load for fat relative to non-fat organs was also determined by fitting a linear mixed effect model, using the nlme package in R, considering mice as random factors. For the statistical analysis of RNA-Seq data, see RNA-Sequencing Section above.

Supplemental References

Cox, M. P., Peterson, D. A., and Biggs, P. J. (2010). SolexaQA: At-a-glance quality assessment of Illumina second-generation sequencing data. BMC Bioinformatics *11*, 485.

Engstler, M., and Boshart, M. (2004). Cold shock and regulation of surface protein trafficking convey sensitization to inducers of stage differentiation in *Trypanosoma brucei*. Genes Dev *18*, 2798-2811.

Hardcastle, T. J., and Kelly, K. A. (2010). baySeq: empirical Bayesian methods for identifying differential expression in sequence count data. BMC Bioinformatics *11*, 422.

Johnson, J. G., and Cross, G. A. M. (1979). Selective cleavage of variant surface glycoproteins from *Trypanosoma brucei.* Biochem J *178*, 689-697.

Kim, D., Pertea, G., Trapnell, C., Pimentel, H., Kelley, R., and Salzberg, S. L. (2013). TopHat2: accurate alignment of transcriptomes in the presence of insertions, deletions and gene fusions. Genome Biol *14*, R36.

Robinson, M. D., McCarthy, D. J., and Smyth, G. K. (2010). edgeR: a Bioconductor package for differential expression analysis of digital gene expression data. Bioinformatics *26*, 139-140.

Taylor, A. E., Lanham, S. M., and Williams, J. E. (1974). Influence of methods of preparation on the infectivity, agglutination, activity, and ultrastructure of bloodstream trypanosomes. Exp Parasitol *35*, 196-208.
