# Supplementary figures and images for: Trypanosoma brucei Parasites Occupy and Functionally Adapt to the Adipose Tissue in Mice
Source: Cell Host Microbe. 2016 Jun 8;19(6):837–48. doi: 10.1016/j.chom.2016.05.002 (PMC4906371; doi:10.1016/j.chom.2016.05.002)

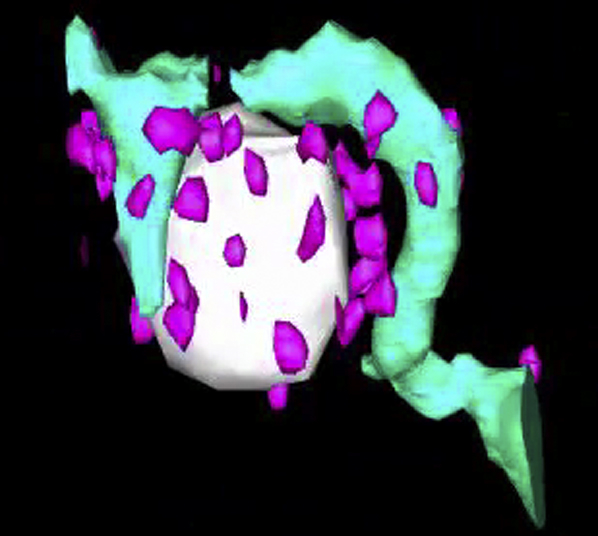

Supplement: Movie S1. 3D-Reconstruction Model of Part of an ATF Parasite — Related to Figure 4. Model constructed from hand-drawn contours marking the boundaries of cellular components in a tomogram of a trypanosome isolated from mouse gonadal adipose. The video is projected using Image J, and various sub-cellular organelles are highlighted through subjectively attributed colors (plasma membrane in yellow, endocytic vesicles in dark blue, glycosomes in pink, nucleus in white, and mitochondrion in green). [file mmc4.jpg]
