# Supplementary material for: Trypanosoma brucei Parasites Occupy and Functionally Adapt to the Adipose Tissue in Mice
Source: Cell Host Microbe. 2016 Jun 8;19(6):837–48. doi: 10.1016/j.chom.2016.05.002 (PMC4906371; doi:10.1016/j.chom.2016.05.002)
Supplement: Document S2. Article plus Supplemental Information [file mmc5.pdf]

Article

Cell Host & Microbe

Trypanosoma brucei Parasites Occupy and Functionally Adapt to the Adipose Tissue in Mice

Graphical Abstract

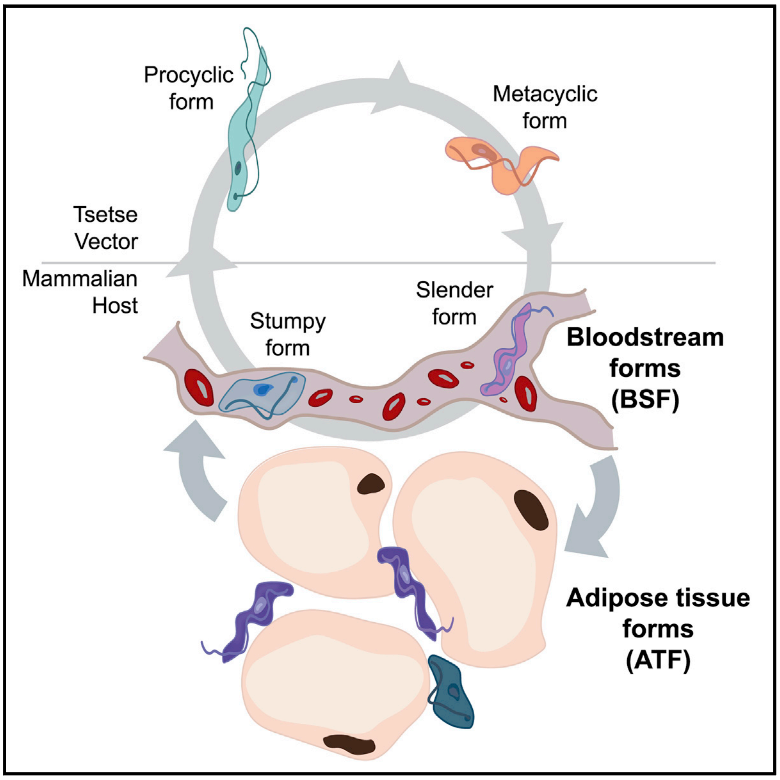

Authors

Sandra Trindade, Filipa Rijo-Ferreira, Tânia Carvalho, ..., Sérgio Dias, Terry K. Smith, Luisa M. Figueiredo

Correspondence

lmf@medicina.ulisboa.pt

In Brief

Trypanosoma brucei is found in the bloodstream and interstitial compartment of several organs in the mammalian host. Trindade et al. uncover the adipose tissue as a major extravascular parasite niche. Extensive remodeling of parasite gene expression in this lipid-rich environment includes upregulation of fatty acid β-oxidation enzymes, suggestive of a functional adaptation.

Highlights

- *T. brucei* parasites accumulate in the adipose tissue early after mouse infection
- Adipose tissue forms (ATFs) can replicate and are capable of infecting naive mice
- ATFs are transcriptionally distinct and upregulate genes for fatty acid metabolism
- ATFs can actively uptake exogenous myristate and form β-oxidation intermediates

Accession Numbers

E-MTAB-1715, E-MTAB-4061

Trindade et al., 2016, Cell Host & Microbe *19*, 837–848
June 8, 2016 © 2016 The Author(s). Published by Elsevier Inc.
http://dx.doi.org/10.1016/j.chom.2016.05.002

CellPress

Trypanosoma brucei Parasites Occupy and Functionally Adapt to the Adipose Tissue in Mice

Sandra Trindade,[1,9] Filipa Rijo-Ferreira,[1,2,3,9] Tânia Carvalho,[1] Daniel Pinto-Neves,[1] Fabien Guegan,[1] Francisco Aresta-Branco,[1] Fabio Bento,[1] Simon A. Young,[7] Andreia Pinto,[1] Jan Van Den Abbeele,[5,6] Ruy M. Ribeiro,[4,8] Sérgio Dias,[1] Terry K. Smith,[7] and Luisa M. Figueiredo[1,*]

[1]Instituto de Medicina Molecular, Faculdade de Medicina, Universidade de Lisboa, 1990-375 Lisboa, Portugal
[2]Department of Neuroscience, University of Texas Southwestern Medical Center, Dallas, TX 75390-9111, USA
[3]Graduate Program in Areas of Basic and Applied Biology, Instituto de Ciências Biomédicas Abel Salazar, Universidade do Porto, 4099-002 Porto, Portugal
[4]Theoretical Division, Los Alamos National Laboratory, Los Alamos, NM 87545, USA
[5]Department of Biomedical Sciences, Unit of Veterinary Protozoology, Institute of Tropical Medicine Antwerp, B-2000 Antwerp, Belgium
[6]Department of Physiology, Laboratory of Zoophysiology, University of Ghent, B-9000 Ghent, Belgium
[7]Biomedical Sciences Research Complex, University of St Andrews, North Haugh, St Andrews, Fife KY16 9ST, UK
[8]Guest Professor, Faculdade de Medicina, Universidade de Lisboa, 1990-375 Lisboa, Portugal
[9]Co-first author
*Correspondence: lmf@medicina.ulisboa.pt
http://dx.doi.org/10.1016/j.chom.2016.05.002

SUMMARY

***Trypanosoma brucei* is an extracellular parasite that causes sleeping sickness. In mammalian hosts, trypanosomes are thought to exist in two major niches: early in infection, they populate the blood; later, they breach the blood-brain barrier. Working with a well-established mouse model, we discovered that adipose tissue constitutes a third major reservoir for *T. brucei*. Parasites from adipose tissue, here termed adipose tissue forms (ATFs), can replicate and were capable of infecting a naive animal. ATFs were transcriptionally distinct from bloodstream forms, and the genes upregulated included putative fatty acid β-oxidation enzymes. Consistent with this, ATFs were able to utilize exogenous myristate and form β-oxidation intermediates, suggesting that ATF parasites can use fatty acids as an external carbon source. These findings identify the adipose tissue as a niche for *T. brucei* during its mammalian life cycle and could potentially explain the weight loss associated with sleeping sickness.**

INTRODUCTION

Human African trypanosomiasis (HAT), also known as sleeping sickness, is a neglected tropical disease that is almost always fatal if left untreated. This disease is caused by *Trypanosoma brucei*, a unicellular parasite that lives in the blood, lymphatic system, and interstitial spaces of organs (reviewed in Kennedy, 2013). Disease pathology often correlates with sites of accumulation of the infectious agent within its host, including the brain, which is associated with characteristic neuropsychiatric symptoms and sleep disorder. Weight loss is another typical clinical feature of sleeping sickness pathology (Kennedy, 2013), but is essentially unstudied.

T. brucei is transmitted through the bite of a tsetse and quickly adapts to the mammalian host to become what is known as a “slender” bloodstream form (BSF). As parasitemia increases, slender forms are capable of sensing population density, and this triggers differentiation to the stumpy form, which is pre-adapted to life in the transmitting tsetse vector and, once there, further differentiates into procyclic form (PCF). Several studies have shown 10%–30% of genes being differentially expressed between BSFs and PCFs (reviewed in Siegel et al., 2011), including genes involved in metabolism, organelle activity, cell-cycle regulation, and endocytic activity. Recent proteomic studies also revealed around 33% of proteins that are developmentally regulated (Butter et al., 2013).

A major difference between BSFs and PCFs is their energy production, with the former utilizing glucose via glycolysis within the glycosome and the latter utilizing proline and, to a lesser extent, other amino acids as their carbon source, via the Krebs cycle in mitochondrion (reviewed in Szöör et al., 2014). To date, no fatty acid β-oxidation has been observed as a carbon source in any life cycle stage of this parasite. This has been a puzzling observation, as the genes required for productive β-oxidation, including the carnitine-acyltransferases (for mitochondrial import of fatty acids), are present in the genome.

Here, we describe an additional form of *T. brucei* in mammalian hosts: we demonstrate that *T. brucei* accumulates in adipose tissue, consistent with recent studies showing accumulation of parasites in the lower abdomen (Claes et al., 2009; McLatchie et al., 2013). Adipose tissue resident *T. brucei* have a different metabolic profile from either slender or stumpy forms in the blood, and this profile is consistent with their utilization of fatty acids (myristate) as a carbon source. These experiments describe an additional form of *T. brucei* life cycle and possibly explain weight loss (wasting), one of the characteristic pathological features of sleeping sickness.

CrossMark

© 2016 The Author(s). Published by Elsevier Inc.
This is an open access article under the CC BY license (http://creativecommons.org/licenses/by/4.0/).

RESULTS

T. brucei Parasites Are Heterogeneously Distributed in Mice

The well-established mouse model (C57BL/6J mice with a pleomorphic clone AnTat1.1^E) was used to confirm weight loss during infection as observed in humans with sleeping sickness. Parasitemia followed a previously described pattern: the first peak of parasitemia occurred 5–6 days post-infection, at around 2×10^8 parasites/mL, and after approximately 4 days of undetectable parasitemia, parasites could be detected again with a fluctuating parasitemia of 10^6–10^7 parasites/mL (Figure 1A). After the first peak of parasitemia, all infected animals showed reduced food intake and a 10%–15% decrease in body weight. Eventually, all mice recovered normal food intake, although their body weight remained 5% lower than that of non-infected animals (Figures 1B and S1A, available online). The weight of most organs from mice sacrificed on days 6 and 28 post-infection showed minimal changes relative to day 0, except fat depots, which decreased on average 43% ± 12%. Spleen size and weight increased dramatically as previously reported (Figure 1C). Infected mice died 35 ± 2.5 days post-infection (Figure 1D).

To assess the parasite load in different organs, we used immunohistochemistry at different days of infection (Figures 1E and S1B). Parasites were consistently detected in the fat 6 days post-infection and at later time points, while in other organs they were seen sporadically and at very low densities. As infection progressed, we observed an increase in parasite load in most organs, with fat, heart, brain, lung, and kidney being the most visibly infiltrated. Parasites were always found extracellularly within the interstitium of these organs. In the brain, our data corroborate the extensively reported evidence for the localization of parasites being restricted to the choroid plexuses and meninges (Kennedy, 2013) (Figure 1E).

Histologically, thymus, lymph nodes, bone marrow, skin (of the head and neck), salivary glands, spleen, gastrointestinal mucosa, testis, and liver displayed few or no parasites (Figure 1E; data not shown). Although parasites in the stroma of the testis were absent, the epidymal fat body and stroma of the epididymis (a small paired organ in the posterior end of the testis) contained a significant number of parasites, many of which appeared as debris, but which could explain the bioluminescence detected by Claes et al. (2009).

Early in Infection, *T. brucei* Accumulate in Adipose Tissue

Immunohistochemical staining showed parasites in the stroma of several fat depots: gonadal, mediastinic, mesenteric, retroperitoneal, perirenal, and interscapular (Figure 2A). Transmission electron microscopy (TEM) confirmed that these parasites were indeed extravascular, as numerous trypanosomes were observed in the interstitial space, either between adjacent adipocytes or between the adipocytes and the capillaries (Figure 2B).

To quantify parasite density, we used as a proxy Trypanosome genomic DNA (gDNA), which was quantified at 6 and 28 days post-infection in the organs/tissues where parasites had been detected by histology, i.e., fat, lung, heart, kidney, brain, and blood (Figures 2C and S2A). The blood had the highest parasite density on day 6. Among solid organs/tissues, for the same day of infection, parasite density was relatively low, except for fat, which had on average 60-fold more parasites than lung, heart, kidney, and brain and 7-fold less than blood. On day 28 of infection, parasite density remained equally high in fat (10^4–10^5 parasites/mg), while it increased, on average, 20-fold in the brain, heart, and lung (Figure 2C). The overall high parasite density was detected in all fat depots characterized in this study, with no significant differences between white and brown fat depots (Figure S2B; Supplemental Experimental Procedures). The blood was the only site where we observed a decrease in parasite density during infection, which is consistent with parasitemia dynamics (Figures 1A and 2C). As a consequence, on day 28, fat was the compartment with the highest parasite density (linear mixed-effects model [LME], $p < 0.0001$).

Overall, the density of parasites per milligram of organ/tissue correlated well with the density calculated as a ratio of parasite gDNA versus mouse gDNA in each tissue (Figure S2C). We also observed essentially the same pattern of parasite density and the same preferential accumulation in the fat when we quantified parasite RNA (qRT-PCR) instead of DNA (Figure S2D), suggesting that gDNA quantification reflects accurately the number of live parasites. Immunohistochemistry also showed accumulation of parasites in fat regardless of parasite strain (EATRO1125 AnTat1.1^E, Lister 427), infection route (intraperitoneal, intravascular), mouse strains (C57BL/6J, BALB/c), animal gender (male, female), or rodent species (mice, rat) (Figure S2E).

Fat represents around 14% of the body weight of a healthy mouse (Jackson Mouse Phenome Database); thus, it is potentially a very large reservoir of parasites. The number of parasites in the organs/tissues (parasite load) was determined by multiplying parasite density by the weight of the organ at the corresponding time of infection. For fat, we used the weight of the six depots characterized in this work, which comprises around 25% of the total body fat. We observed that while 6 days post-infection, the blood contained the majority of the parasites (around 10^8 parasites), on day 28 the six depots of fat contained overall more parasites than the blood, brain, and all other tested organs combined (LME, $p < 0.0001$) (Figure 2D). A similar preference for accumulation in fat was observed when the mouse infection was initiated by a tsetse bite, which deposits metacyclic forms in the skin of the mouse (Figure S3) (LME, $p < 0.0001$). Overall, these data revealed that fat represents a major reservoir of parasites, regardless of whether the infection was initiated by BSFs or metacyclic forms.

Adipose Tissue Contains Replicative and Infective Parasites

In the blood, parasites can be either replicative slender, G1-arrested stumpy forms or intermediate forms that are not fully differentiated (reviewed in MacGregor et al., 2012). To investigate whether the parasites from fat (referred to hereafter as adipose tissue forms [ATFs]) are replicative or not, we infected mice with a *GFP::PAD1*$_{utr}$ reporter cell-line, in which a *GFP* gene is followed by a *PAD1* 3′ UTR that confers maximum expression in stumpy forms (J. Sunter, A. Schwede, and M. Carrington, personal communication; MacGregor et al., 2012). Four and six days post-infection, blood and fat were collected, and parasites isolated and purified. As described by MacGregor et al. (2011), on day 4 we observed that most

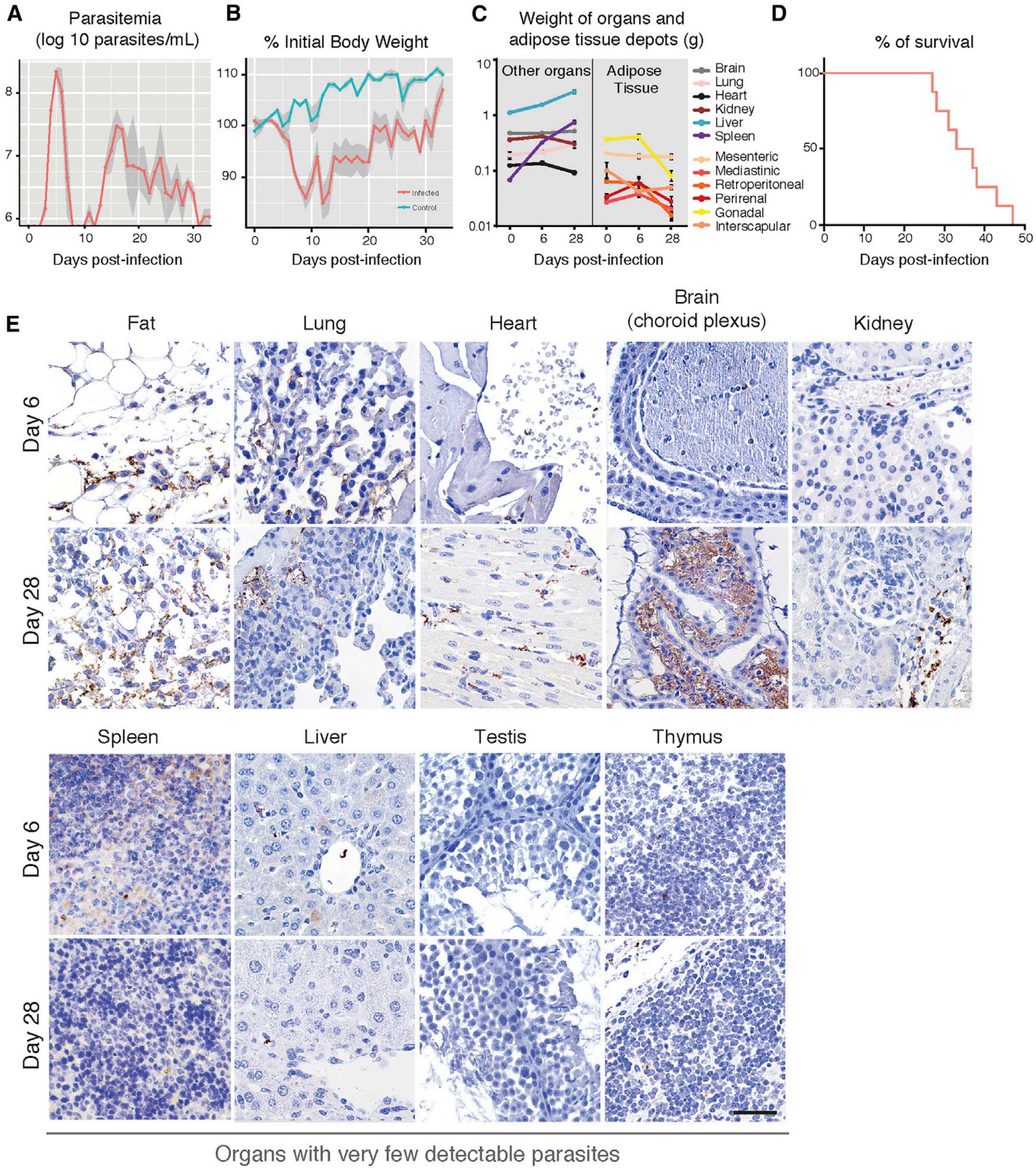

Figure 1. Tissue Distribution of *T. brucei* during a Mouse Infection Is Heterogeneous

(A) Mean parasitemia profile of 20 mice infected with *T. brucei* AnTat1.1^{E}. Parasitemia was assessed from tail blood using a hemocytometer (limit of detection is around 4×10^5 parasites/mL). Light gray shaded area represents SEM.

(B) Variation of body weight during infection. Daily body weight measurement of control and infected mice (n = 15 per group). Light gray shaded area represents SEM.

(C) Variation of organ weight during infection (n = 4 per group).

(D) Survival curve of *T. brucei* infected mice (n = 8).

(E) Representative brightfield micrographs of *T. brucei* distribution in several organs/tissues at days 6 and 28 post-infection, assessed by immunohistochemistry with a non-purified rabbit anti-VSG antibody (parasites appear in brown). n = 5 per time point. Scale bar, 50 μm.

See also Figures S1 and S2.

parasites in the blood were GFP negative (98% ± 0.3%), while on day 6 most parasites expressed high levels of GFP (86% ± 2.6%). Interestingly, from day 5, we noted the presence of parasites expressing lower levels of GFP, which likely correspond to differentiating intermediate forms (data not shown). As the yield of isolation of ATF parasites was very low on days 4 and 5, we analyzed these parasites only on day 6. The majority of the ATF parasites were GFP negative (79% ± 4.6%), while 21% ± 4.6% expressed GFP, indicating that on day 6 fat contains fewer stumpy/intermediate forms than blood (Figure 3A).

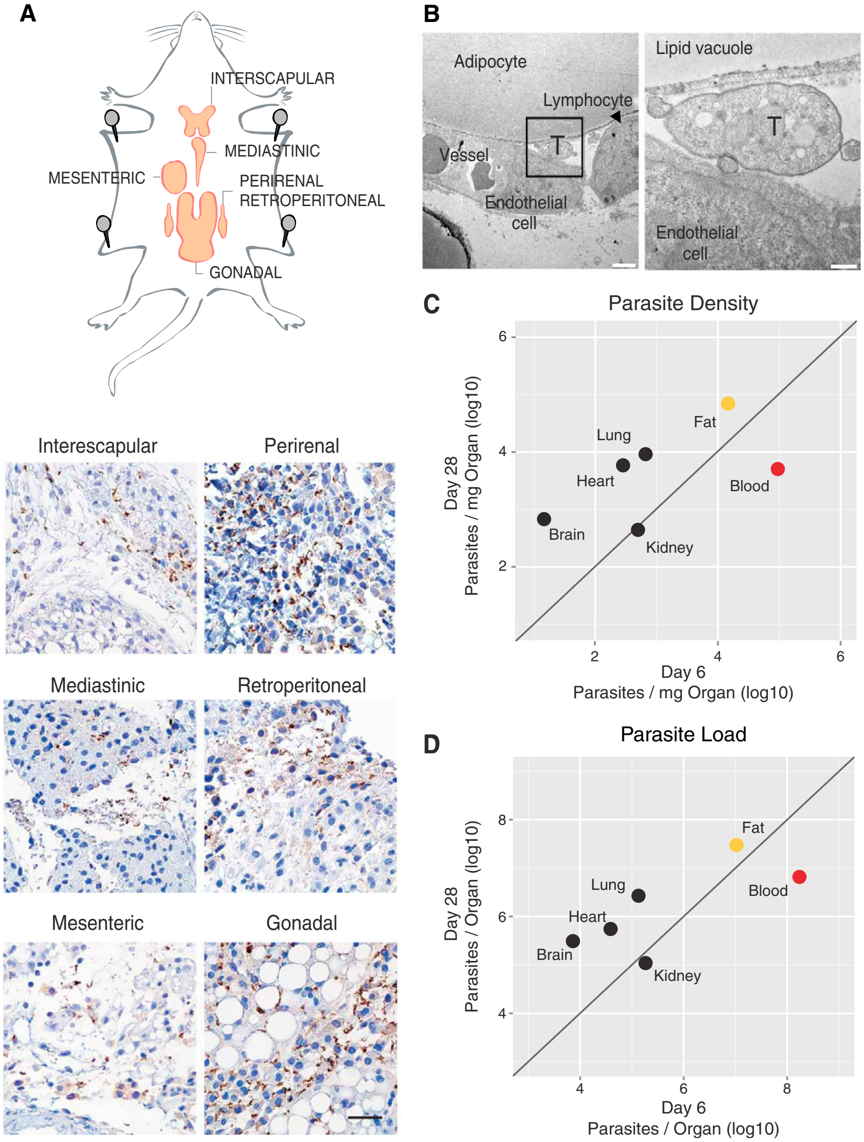

Figure 2. Fat Depots Are a Major Parasite Reservoir

(A) Schematic representation of mice fat depots and anti-VSG immunohistochemistry images of six different fat depots, collected 28 days post-infection. Scale bar, 50 μm.

(B) Transmission electron micrograph of a gonadal fat depot 6 days post-infection. Trypanosome (T) and lymphocyte in the interstitial space, adjacent to an adipocyte and next to a small capillary. Scale bars, 2 and 0.5 μm in the left and right panels, respectively.

(C) Parasite density in multiple organs/tissues (6 and 28 days post-infection) was measured by qRT-PCR of gDNA (quantification of *T. brucei* 18s rDNA relative to the tissue/organ weight). Blood density was assumed 1.05 g/mL. Fat value is the average of quantification of the six depots indicated in (A). Each point represents the geometric mean of the parasite density on days 6 (n = 3–9) and 28 post-infection (n = 3–6).

(D) Parasite load in multiple organs/tissues estimated by multiplying parasite density with organ weight at the corresponding day of infection. Each point represents the geometric mean of the parasite density on days 6 (n = 3–9) and 28 post-infection (n = 3–6).

See also Figures S1–S3.

To confirm if GFP-negative ATF parasites are replicative and GFP-positive parasites are cell-cycle arrested, we stained the parasite nuclear DNA with propidium iodide and quantified it by flow cytometry. In all samples (blood day 4, blood day 6, and fat day 6), we observed that GFP-negative parasites displayed a cell-cycle profile characteristic of replicative cells (around 60%–70% of cells in G1, 5% in S-phase and 20%–30% in G2/M), while GFP-positive cells were cell-cycle arrested in G1/G0 (90%–95%) (Figure 3B). Similar data were obtained by performing cell-cycle analysis with DyeCycle Violet (Figures S4A and S4B), further confirming the presence of slender and stumpy/intermediate forms in fat.

To validate the presence of stumpy/intermediate forms in fat, we used fluorescence microscopy on an intact gonadal depot infected with *GFP::PAD1*$_{utr}$-expressing parasites (Figure 3C). LipidTOX stains the lipids in the large lipid droplet of adipocytes. Among the adipocytes, we could clearly observe many green foci, which represent the parasite nuclei where GFP accumulates, thus confirming the presence of GFP-positive parasites (stumpy and/or intermediate forms) in close proximity to adipocytes. The presence of replicating parasites in intact tissue was confirmed by immunohistochemistry with an anti-H2A antibody. Dividing nuclei were clearly visible in close proximity to adipocytes (Figure S4C), further confirming the fluorescence-activated cell sorting (FACS) cell-cycle data.

To test whether ATF parasites are capable of establishing a new infection, an infected donor mouse was sacrificed and perfused, and several organs were collected, homogenized, and injected intraperitoneally into recipient naive mice. Parasitemia was assessed daily thereafter and scored on the first day it became detectable (Figure 3D). Mice that received blood or a fat homogenate showed parasitemia earlier (around 3 days post-transplantation) than animals injected with heart and brain homogenates (around 4 days post-transplantation), consistent with the observed parasite load in these organs (Figures 2D and 3D). Transplant of intact gonadal fat depot also led to successful infection of the recipient naive mice (data not shown), suggesting that parasites can exit from an intact tissue. These results showed that parasites from fat, heart, and brain are capable of reinvading the bloodstream and establishing a new infection.

Morphology of Adipose Tissue Forms

Although *T. brucei* is always extracellular, its morphology changes during the life cycle, which may reflect a specific adaptation to the host niche (Wheeler et al., 2013; Bargul et al., 2016).

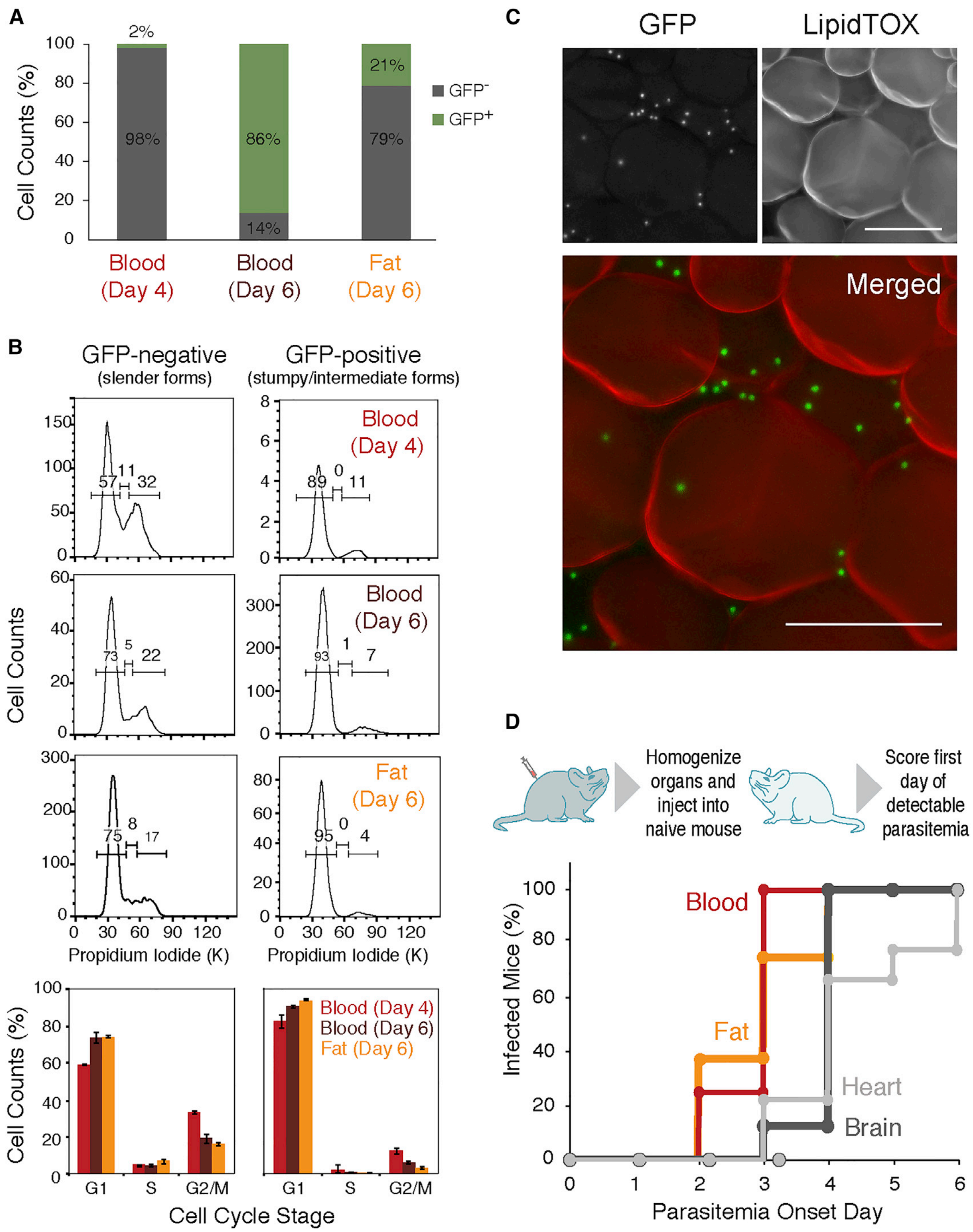

Figure 3. Fat Harbors Replicative Forms that Can Establish a New Infection

(A) Frequency of GFP expression measured by flow cytometry in parasites isolated from blood and fat, 4 and 6 days post-infection with a *GFP::PAD1$_{utr}$ T. brucei* reporter cell line (n = 2–3).

(B) Cell-cycle analysis assayed by flow cytometry of propidium iodide-stained parasites (n = 2–3). The values represented are the means of the percentage of the cell population in each cell-cycle stage and their SEM.

(C) Fluorescence microscopy of gonadal adipose tissue from a mouse infected for 6 days with *GFP::PAD1$_{utr}$* reporter cell line. Lipid droplets were stained with LipidTOX (red), and nuclei of GFP-expressing parasites (stumpy and/or intermediate forms) are green. Scale bar, 50 μm.

(D) Onset of parasitemia curves in mice that were injected intraperitoneally with infected organs/tissues lysates from a donor mouse. Lysates from blood, heart, brain, and gonadal fat depot were prepared from mice sacrificed between 21 and 28 days post-infection to ensure presence of a larger number of parasites (n = 9). See also Figure S4.

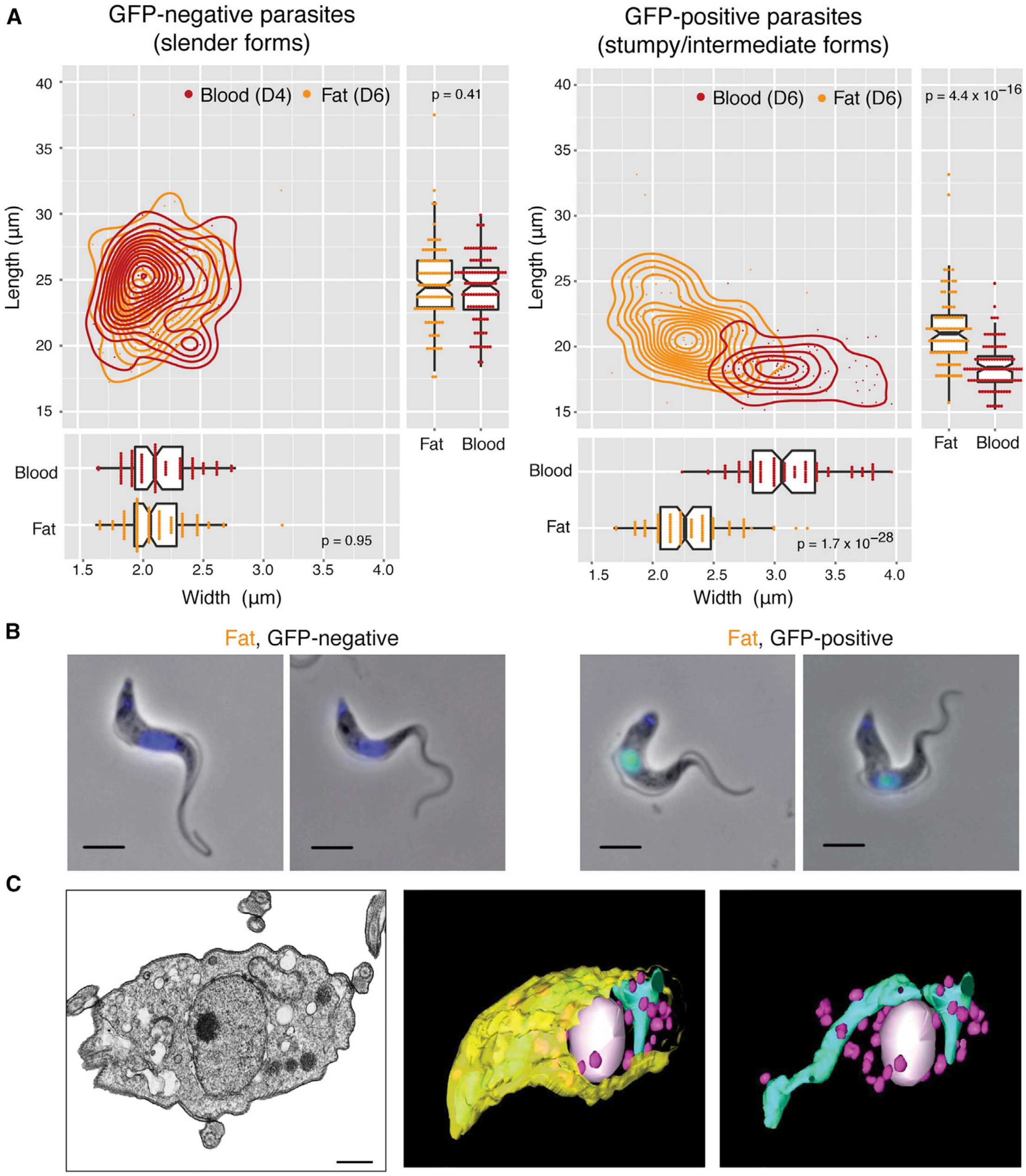

Figure 4. Fat Is Populated by Slender, Intermediate, and Stumpy Forms

(A) Morphological features (length and width) of fixed parasites isolated from fat and blood of mice infected with *GFP::PAD1*$_{utr}$ reporter. Fat gonadal tissue was collected on day 6 post-infection. The blood "controls" were obtained as follows: GFP-negative parasites were collected on day 4 post-infection (mostly slender forms), and GFP-positive parasites were collected on day 6 post-infection (mostly stumpy forms). Morphometric measurements were scored from phase contrast microscopy images, analyzed via HTIAoT, and confirmed by manual measurement. GFP negative, slender form; GFP positive, stumpy and intermediate forms. n = 100 per group, from three independent mouse experiments. Statistical significance was assessed using a Wilcoxon rank-sum test.

(B) Representative images of parasites isolated from fat. Replicating parasites (such as the second from the left) were excluded from morphometric analysis. DNA was stained with DAPI (blue). GFP protein (green) is localized in the nucleus of intermediate and stumpy forms. Scale bar, 4 μm.

(C) Transmission electron micrograph and 3D tomography images of a parasite isolated from gonadal adipose tissue. Mitochondrion is represented in cyan, glycosomes in pink, nucleus in white, and plasma membrane in yellow. Scale bar, 500 nm.

See also Figure S5 and Movie S1.

ATF parasites, like BSFs, have an undulating appearance, with a flagellum attached to the cell body and with kinetoplast DNA positioned between nucleus and flagellar pocket (Figure 4B).

To characterize in more detail the morphology of ATF parasites, we compared the length and width of *GFP::PAD1*$_{utr}$ parasites isolated from adipose tissue (day 6 post-infection) and

blood (days 4 and 6 post-infection) (Figures 4A and 4B). Automatic measurements of phase contrast microscopy images were generated via HTIAoT (Wheeler et al., 2012) and confirmed with manual measurements (Figure S5A). We observed that slender forms (GFP negative) from blood and adipose tissue were very similar both in length (blood, 24.39 ± 2.50 μm; fat, 24.57 ± 2.99 μm) and width (blood, 2.15 ± 0.26 μm; fat, 2.12 ± 0.26 μm). This average length is consistent with previous reports (Tyler, 1998; Tyler et al., 2001; Bargul et al., 2016). In blood day 6 post-infection, GFP-positive parasites were, as expected, shorter (18.43 ± 1.81 μm) and wider (3.11 ± 0.38 μm) than slender counterparts of day 4, corresponding to the morphology of stumpy forms (Tyler et al., 2001). Interestingly, in adipose tissue we found not only stumpy forms, but also GFP-positive parasites that morphologically were in between slender and stumpy forms (length, 21.32 ± 2.73 μm; width, 2.29 ± 0.31 μm) (Tyler, 1998). These probably correspond to the previously described blood intermediate forms, which, as the name suggests, are not fully differentiated into stumpy forms, but could already express GFP::PAD1 (MacGregor et al., 2011, 2012). These results indicate that adipose tissue is populated by parasites whose morphology has been previously found in the blood. The only significant difference is their relative distribution: on day 6 of infection, while blood is mostly populated by stumpy forms, adipose tissue appears to be "delayed," as we detected both intermediate and stumpy forms.

At the ultrastructure level, ATF parasites contain all major structures described in other stages of life cycle (Gull, 1999), including an electron-dense coat, nucleus, mitochondrion, endoplasmic reticulum, Golgi apparatus, glycosomes, dense granules and numerous vesicles compatible with endosomes, an internal subpellicular corset of microtubules underneath plasma membrane, and a flagellum attached to the cell body (Figures 4C and S5B). Using serial 3D tomography, we observed that the single mitochondrion of ATF parasites occupies a small volume of parasite body and is not highly branched (Figure 4C; Movie S1). This organization was confirmed by Mitotracker Green staining, which showed no major differences between the mitochondrion of parasites in blood and adipose tissue (Figure S5C; Movie S1).

Transcriptome of ATF Parasites Reveals Differences in Several Key Regulatory Processes

During its life cycle, *T. brucei* adapts to its environment by changing gene expression (reviewed in Siegel et al., 2011). To test whether parasites within fat also adapted to the new environment, total RNA was extracted from infected gonadal fat depot (n = 3) on day 6, along with parasites from blood (n = 2) on day 4 (maximizing slender and minimizing stumpy/intermediate forms), and was subjected to RNA sequencing (RNA-seq) analysis. As expected, sequence reads from blood samples corresponded mainly to parasite transcripts, while sequence reads from fat corresponded mainly to host transcripts. Nevertheless, the 1%–9% of the sequence reads from *T. brucei* provided enough statistical power to detect changes in the transcriptomes of ATF parasites (Table S1). Two previously published RNA-seq datasets of BSF parasites grown in culture were also included in this analysis (Pena et al., 2014).

Unbiased clustering of gene expression profiles revealed that ATF parasites clustered separately from parasites isolated from blood or culture (Figure 5A), suggesting significant changes in their overall transcriptome. Changes were identified using three methods of differential expression analysis, and only those genes identified by at least two methods with an adjusted p value <0.01 were considered. These analyses showed that 2,328 genes (around 20% of transcriptome) were differentially expressed between BSF and ATF parasites: 1,160 were upregulated in ATF parasites and 1,178 were upregulated in BSFs (Figures 5B and 5C; Table S2).

Significant changes were found in genes involved in gene expression regulation, cell cycle, and cell signaling (Table S3). RNA-binding proteins play an important role in gene expression and differentiation throughout the *T. brucei* life cycle. For example, RNA-binding protein 42 (RBP42; TriTrypDB: Tb927.6.4440, http://tritrypdb.org/tritrypdb/) binds many mRNAs involved in cellular energy metabolism (Das et al., 2012). Upregulation of RBP42 in ATF parasites could be involved in the metabolic rewiring when parasites enter the fat (Table S3).

ATF parasite transcriptome also showed dramatic changes in gene expression of various post- and co-translational modifying enzymes that might have considerable influence on diverse cellular processes (Table S3). A small number of genes potentially acting in the cell cycle and cytokinesis was identified with significant differential expression, including the cytoskeleton-associated AIR9 protein and spastin, which were upregulated (Table S3), suggesting differences in cell-cycle regulation in these parasites. Consistent with a minor stumpy form population in fat, we did not find enrichment of stumpy-specific genes in the transcriptome of ATF parasites. Extracellular signaling mechanisms also seem to be affected in ATF parasites, including upregulation of TOR3, which can relate the supply of external nutrients to internal energy levels to regulate cellular growth (de Jesus et al., 2010).

Interestingly, although by TEM an electron-dense coat can be observed around the parasite (Figure 2B), we found that the transcript levels of the active variant surface glycoprotein (VSG) (VSG AnTat1.1, CAA25971.1) are 3-fold downregulated in adipose tissue, suggesting VSG downregulation or VSG switching within the tissue. As the VSGnome of AnTat1.1E clone is currently unknown, we could not test whether silent VSGs were upregulated as a compensatory mechanism. Genes encoding for other surface molecules, such as the haptoglobin receptor and most procyclins, were not differentially expressed.

Transcriptome of ATF Parasites Reveals Metabolic Adaptations

One of the most evident changes in ATF transcriptome was the upregulation of many metabolic pathways, including glycolysis, pentose phosphate, purine salvage, sterol and lipid metabolism, and, surprisingly, β-oxidation. Thirteen of the 14 enzymatic steps of glycolysis were upregulated relative to BSFs (Table S3). This may either be a response to the lower glucose concentration in fat interstitial fluid relative to bloodstream, or an upregulated gluconeogenesis, which relies mostly on the same enzymes.

In ATF parasites, genes involved in three out of the five biosynthetic steps in the pentose-phosphate pathway were upregulated, including the rate-limiting glucose-6-phosphate

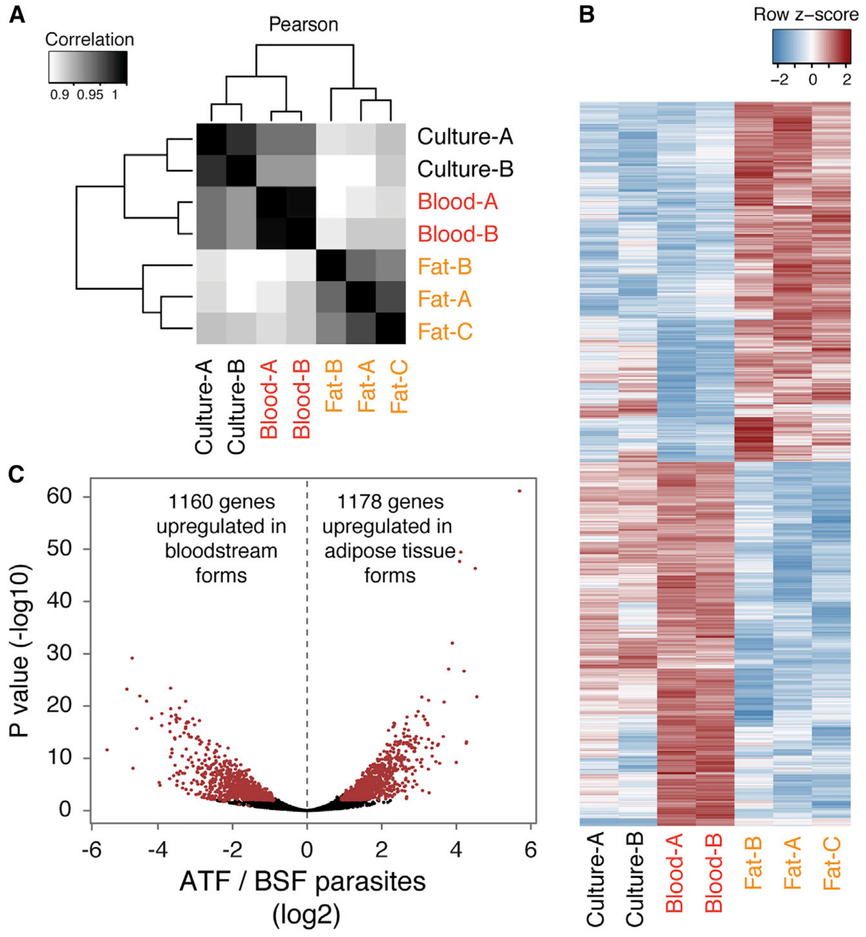

Figure 5. ATF Parasites Are Transcriptionally Different from BSFs

(A) Hierarchically clustered heat map of Pearson correlations of transcript levels (log_2 transformed RPKM) from independent RNA-seq datasets: Lister427 parasites grown in culture (Pena et al., 2014) (n = 2), parasites isolated from blood of AnTat1.1-infected mice on day 4 post-infection (n = 2), and parasites isolated from gonadal fat on day 6 post-infection (n = 3).

(B) Heat map view of relative transcript levels for differentially expressed genes from culture and in vivo in parasites isolated from the two tissues (adjusted $p < 0.01$ in at least two of three methods).

(C) Volcano plot displaying in red the differentially expressed genes represented in (B). Displayed p values and fold changes are from DESeq2.

See also Tables S1 and S2.

dehydrogenase (TriTrypDB: Tb927.10.2490). This observation, taken together with the fact that numerous enzymes (16 in total) involved in purine salvage pathway were also upregulated, suggests that ATF parasites may increase purine production. Interestingly, the purine phosphatases (TriTrypDB: Tb927.8.3800 and Tb927.7.1930) and cAMP phosphodiesterase PDEA (TriTrypDB: Tb927.10.13000) are up- and downregulated, respectively, suggesting that the increased purine production may be directed toward cAMP signaling (Table S3).

ATF parasites showed significant upregulation of the alanine and aspartate aminotransferases and the glutamate shunt, which feed products into the tricarboxylic acid (TCA) cycle. Additionally, this cycle also appeared to be more active, given the upregulation in three key steps, allowing it to process succinate, fumarate, and 2-oxoglutarate, resembling the TCA cycle of the *T. brucei* insect form (reviewed in Szöör et al., 2014). These changes suggest that the F0/F1 ATP synthase complex is functional and that the associated electron transport chain is operating in ATF parasites in a manner similar to that in PCF parasites.

Significant changes in gene expression of lipid and sterol metabolic pathways were also observed in ATF parasites. However, one of the most striking observations in the RNA-seq data was the potential presence of an active fatty acid β-oxidation, which produces energy from fatty acid catabolism. This was unexpected, as β-oxidation activity has never been detected in any *T. brucei* life cycle stage to date. ATF parasites showed upregulation of the putative genes responsible for the second and fourth steps of the β-oxidation cycle (enoyl-CoA hydratase and 3-ketoacyl-CoA thiolase, respectively) (Figure 6A). Moreover, fatty acid transport across the mitochondrial membrane (facilitated by acyl-CoA synthases and carnitine-acyltransferases) was upregulated, while fatty acid elongases 2 and 4 were downregulated, suggesting that in ATF parasites, endocytosed fatty acids are not being elongated and anabolized into more complex molecules. Instead, they may enter the glycosomes and/or mitochondrion, where they are catabolized via a β-oxidation pathway to form acetyl-CoA (experimentally validated; see below and Figure 6), which feeds into the now-active TCA cycle.

ATF Parasites Have Active Fatty Acid β-Oxidation

To investigate whether ATF parasites are capable of β-oxidation, labeled myristate was used in a pulse-chase experiment with living trypanosomes, and potential labeled β-oxidation intermediates were identified by gas chromatography-mass spectrometry (GC-MS). Myristate (C14:0) was chosen, as it is efficiently taken up and incorporated into lipids and glycosylphosphatidylinositol (GPI) anchors in the slender BSF parasites (reviewed in van Hellemond and Tielens, 2006). Isolated ATF parasites were labeled with deuterated myristate (D_{27}-C14:0) for 1 hr and then chased with serum, following which labeled myristate metabolites were identified. As expected, BSFs showed accumulation of D_{27}-C14:0 during the pulse and chase periods (Figures 6B and 6C). ATF parasites also showed D_{27}-C14:0 accumulation during the pulse (Figure 6B, upper panel, and Figure 6C), but the amount of D_{27}-C14:0 decreased significantly during the chase (Figure 6B, lower panel, and Figure 6C). Importantly, the decrease of

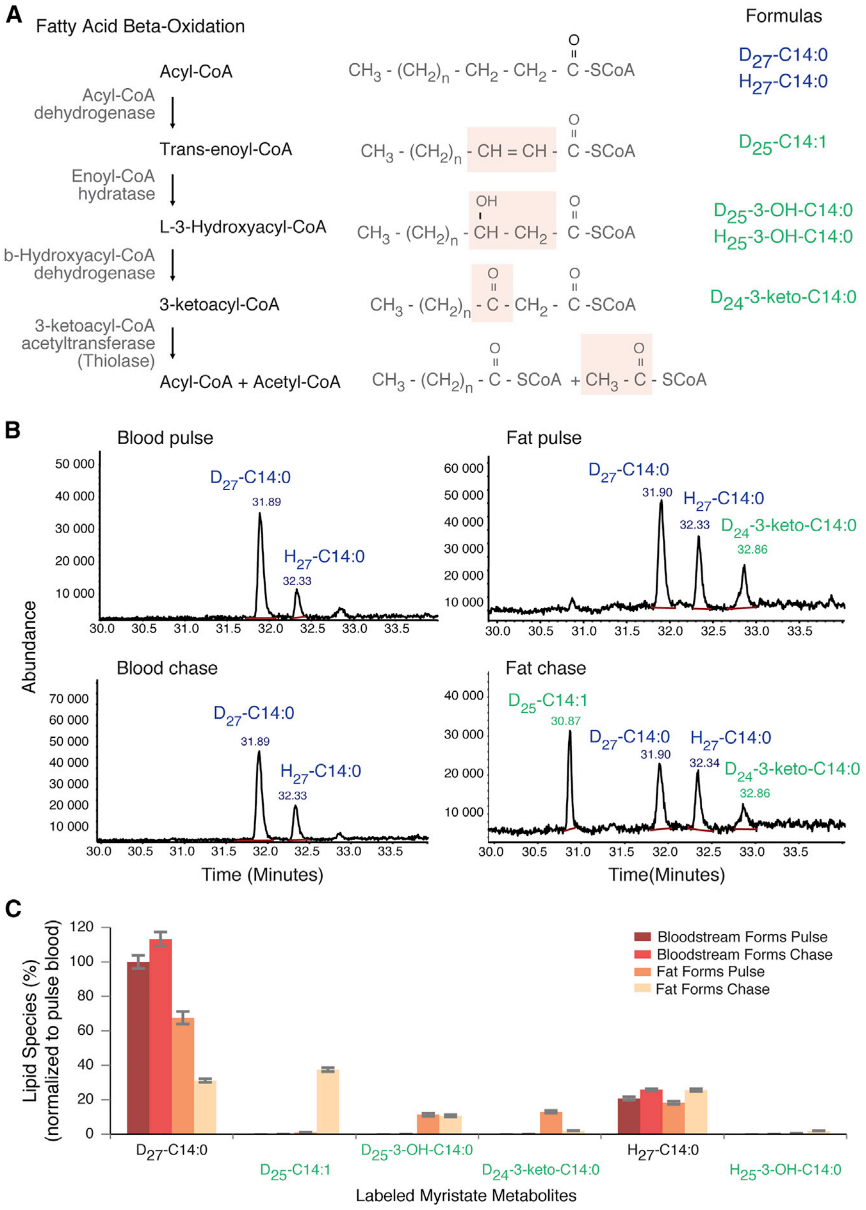

Figure 6. Fatty Acid β-Oxidation Is Active in ATF Parasites

(A) Schematic of fatty acid β-oxidation pathway. Four enzymatic modifications are indicated by shaded box on the fatty acid structures where biotransformation takes place. Formulas in blue and green indicate the myristate and β-oxidation metabolites from the non-labeled and labeled myristate, respectively, identified in this work.

(B) Fatty acid methyl ester (FAME) analysis by GC-MS of D_{27}-C14:0-labeled BSF (left) or ATF (right) parasites for 1 hr (upper) and chased for a further 1 hr (lower). GC-MS trace shows 30–34 min (n = 3).

(C) Uptake of D_{27}-C14:0 and β-oxidation metabolites after normalization to the added internal standard C17:0. 100% equates to the amount of D_{27}-C14:0 taken up by bloodstream form in the 1 hr labeling (pulse) (n = 3). The values represented are the means and the respective SEM.

See also Figures S6 and S7.

D_{27}-C14:0 in ATF parasites coincided with the detection of β-oxidation metabolites derived from the labeled myristate, including myristoleic acid (D_{25}-C14:1), 3-hydroxy-myristate (D_{25}-3-OH-C14:0), and 3-oxo-myristic acid (D_{24}-3-keto-C14:0) (Figure 6B, right panels, and Figures 6C, S6, and S7). The latter two metabolites were also observed to some minor extent during the pulse (Figure 6B, upper panel, and Figure 6C), while D_{25}-C14:1 was present in higher amount during the chase period. Minor amounts of unlabeled 3-hydroxy-myristrate (H_{26}-3-OH-C14:0) were also observed in ATF parasites, but not BSFs (Figure 6C).

Collectively these data show that ATF parasites are able to actively take up exogenous myristate and form β-oxidation intermediates, demonstrating the existence of this pathway in trypanosomes and suggesting that ATF parasites could in part use fatty acid β-oxidation to satisfy their energy requirements.

DISCUSSION

A well-established feature of the unicellular, extracellular *T. brucei* parasite is its ability to invade the CNS. Here, we show that while blood is the major site of parasite accumulation on the first peak of parasitemia, fat contains the highest density and total number of parasites later in infection (around 100- to 800-fold more than the brain). Although the reason(s) why parasites accumulate in adipose tissue remain unknown, we clearly show it has dramatic consequences for the parasites. They functionally adapt to the tissue environment by rewiring gene expression, including the possibility of using lipids/fatty acids as a carbon source.

Possible Advantages to Parasite Accumulation in the Adipose Tissue

Accumulation in the adipose tissue could be due to several non-mutually exclusive reasons that may have provided a selective advantage during evolution: parasites may be less efficiently eliminated by adipose tissue-specific immune response, parasites may grow at a faster rate, parasite differentiation may be delayed, and/or parasite entry in adipose tissue may be more efficient in adipose tissue than in other organs/tissues. Depending on the dynamics of parasite movement to/from adipose tissue, it is possible that fat acts as a source of parasites that can repopulate the blood. This reversible movement between blood and fat could have important implications for (1) the transmission dynamics, since stumpy formation is triggered by a quorum-sensing mechanism (MacGregor et al., 2011), and (2) antigenic variation, if fat, for example, would favor the appearance of new VSG variants that could later go to the blood (Mugnier et al., 2015).

An intriguing question is whether stumpy forms could be directly ingested by a tsetse fly from the subcutaneous fat.

Although in our histological analysis we did not find a significant number of parasites in this fat depot, it is possible we missed a preferential skin location. Moreover, we performed this analysis in mice infected by intraperitoneal injection, which bypasses the skin as the first entry point of metacyclic forms. So it remains to be determined whether subcutaneous fat is important for accumulation of metacyclics and/or ATF parasites and how it impacts transmission.

Not all *Trypanosoma* species occupy the same niche in the host. *T. brucei* and *T. evansi* are mainly tissue-invading parasites, while *T. congolense* stays in smaller capillaries and venules of tissues and *T. vivax* remains mainly in circulation (Losos and Ikede, 1972). These differences have been associated with the different swimming properties of each *Trypanosoma* species (Bargul et al., 2016). Previous reports had indirectly suggested that *T. brucei* parasites could be present in adipose tissue (Fernandes et al., 1997; Giroud et al., 2009). Our study demonstrates that *T. brucei* parasites accumulate in high numbers in the fat of rodents. Although mouse is an accepted model to study *T. brucei* infection (Giroud et al., 2009), we cannot exclude the possibility that accumulation in adipose tissue is a result of the selection process that happens when *T. brucei* infects a non-natural host. In the future, it would be interesting to confirm whether fat preference is a common feature of this and other *Trypanosoma* species in their natural hosts and to compare their swimming properties in different tissues/organs.

It is intriguing to note that several pathogens infect adipose tissue. *T. cruzi*, the causative agent of Chagas disease in Latin America, invades adipocytes during acute infections in mice and humans (Ferreira et al., 2011). Also, *Plasmodium berghei*, a causative agent of rodent malaria, sequesters in lungs and fat (Franke-Fayard et al., 2005). *Mycobacterium tuberculosis* infects adipocytes, where it accumulates in intracytoplasmic lipid inclusions and survives in a "dormant" non-replicating state that is insensitive to anti-mycobacterial drugs (Neyrolles et al., 2006). HIV takes advantage of the fat as a viral reservoir during the chronic stage of infection, and persistence on this reservoir is an obstacle for treatment (Chun et al., 2015). It is possible that persistence of *T. brucei* in the fat may also account for some of the treatment failures in humans (Richardson et al., 2016).

Functional Adaptation to Host Adipose Tissue

A major observation in this work is that 20% of the genes are differentially expressed between ATFs and BSFs, which is comparable with the differences between BSFs and PCFs (around 30%) and between slender and stumpy BSF forms (around 12%) (reviewed in Siegel et al., 2011). Parasites adapt to the fat environment by changing transcript levels of genes involved in metabolism, signaling, cell-cycle control, and RNA binding. Using biochemical assays, we confirmed that ATF can utilize fatty acids, i.e., myristate, and catabolize them via β-oxidation, which could lead to the production of ATP via the TCA cycle and oxidative phosphorylation. Therefore, it seems that parasites can sense and adapt to the adipose environment by rewiring their gene expression, including the ability to use lipid/fatty acid as a carbon source.

The major carbon source of BSF and PCF parasites is glucose and proline, respectively, both of which are readily available nutrients in the host (reviewed in Szöör et al., 2014). Fat is, in its essence, a lipid-rich environment. Therefore, it is possible that fatty acids or some other form of lipid are released from the host adipocytes and are endocytosed or actively transported via a receptor-mediated process by ATF parasites (Vassella et al., 2000). So far, only one receptor has been identified in *T. brucei* as necessary for the import of LDL particles (Coppens et al., 1987). Its transcript levels are not altered in ATF parasites, suggesting either that this protein can be upregulated post- or co-translationally or that lipid/fatty acid import is mediated by yet-uncharacterized transporters.

Consumption/utilization of host's lipids during a *T. brucei* infection could contribute to the weight loss observed in patients with sleeping sickness, cattle with Nagana, and mice infected with *T. brucei* (Kennedy, 2013; Ranjithkumar et al., 2013). Interestingly, obese mice ($db^{-/-}$ knockout mice) infected with *T. brucei* live 3-fold longer than their littermates, suggesting that having more adipose tissue partially protected mice from a *T. brucei* infection (Amole et al., 1985). Because obesity is associated with persistent low-grade chronic inflammation in adipose tissue (Ouchi et al., 2011), it is possible that in obese mice, parasites get more efficiently eliminated (or controlled), thus prolonging the survival of the host.

Most of what is known today about the mechanisms of virulence, persistence, and transmission of *T. brucei* results from studies performed in BSF parasites. The identification of adipose tissue as an additional major reservoir of functionally differentiated *T. brucei* brings a unique perspective to our understanding of this parasite and raises several questions. What is the relative contribution of BSF and ATF parasites for pathogenicity and host metabolic alterations? Could fat act as a source of parasites expressing novel VSGs? What are the implications of such a large reservoir of ATF parasites in terms of transmission? What are the dynamics of parasite entry and exit from fat? Given that the brain is a lipid-rich organ, which is also invaded by *T. brucei*, it is obvious to ask whether these parasites also adapt their gene expression and how this impacts brain-associated pathology. Do ATF parasites induce changes in the host metabolism, providing an advantage to the parasitic infection? Is the immune response of the adipose tissue more permissive to *T. brucei* parasites? Are anti-trypanosome drugs equally efficient at eliminating ATFs and BSFs? In sum, our findings have important consequences for the understanding of parasite biology, disease, and drug treatment efficacy.

EXPERIMENTAL PROCEDURES

Detailed experimental procedures can be found in Supplemental Experimental Procedures.

Animal Experiments

Animal experiments were performed according to EU regulations and approved by the Animal Ethics Committee of Instituto de Medicina Molecular (AEC_2011_006_LF_TBrucei_IMM). Tsetse fly infections were performed in compliance with the regulations for biosafety and animal ethics (VPU2014_1) and under approval from the environmental administration of the Flemish government. Unless otherwise indicated, all infections were performed in wild-type male C57BL/6J mice, 6–10 weeks old (Charles River, France), by intraperitoneal injection of 2,000 *T. brucei* AnTat 1.1^{E} 90-13 parasites. For parasite counts, blood samples were taken daily from the tail vein. Organs/tissues of infected mice were collected at days 6 and 28 post-infection unless otherwise stated. Animals were sacrificed by CO_2 narcosis, blood collected

by heart puncture, and mice immediately perfused. Collected organs were snap frozen in liquid nitrogen or fixed in 10% neutral-buffered formalin. In transplants, homogenates as well as 600 μL of blood were transplanted into age- and sex-matched naive mice.

Histology and Electron and Fluorescence Microscopy

Formalin-fixed organs were immunostained with a non-purified rabbit serum anti-*T. brucei* VSG13 antigen (which crossreacts with many VSGs) and a non-purified rabbit serum anti-*T. brucei* H2A. For TEM, ultra-thin sections (70 nm) were screened in a Hitachi H-7650 microscope. 3D reconstruction of isolated trypanosome was done using the IMOD software package version 4.7.3 for alignment and modeling (Kremer et al., 1996).

For fluorescence analysis, the gonadal depot was stained with LipidTox, fixed in 10% neutral-buffered formalin and embedded in Fluoromount-G. Fluorescence images were taken using a 40× objective in a Zeiss Cell Observer wide-field microscope. For morphometry analysis, isolated parasites were fixed with paraformaldehyde, DAPI stained, and embedded in vectashield. Images were taken using a 63× oil objective with optional optovar magnification (1.6×) in the same wide-field microscope. Parasite measurements were taken essentially as described in Wheeler et al. (2012).

The mitochondrion of isolated parasites was labeled using MitoTracker Green (Invitrogen/Molecular Probes, M-7514) according to the manufacturer's instructions. Fluorescence and DIC images were acquired using a confocal laser point-scanning microscope (Zeiss LSM 710).

Parasite Quantification

T. brucei 18S rDNA genes were amplified from gDNA of a known mass of tissue and converted into parasite number using a standard curve. For RNA quantification, the ΔΔCt method was used by amplifying *TbZFP3* and mouse *Gapdh* genes from tissue total RNA.

Parasite Isolation from Tissues

Bloodstream parasites were purified over a DEAE column (Taylor et al., 1974), while ATF parasites were isolated from gonadal fat depot by incubating the depot in MEM or HMI11 at 37°C and 150 rpm agitation for up to 40 min.

Flow Cytometry

Cell-cycle analysis was performed using propidium-iodide (PI) or Vybrant DyeCycle violet (DCV) in fixed or live cells, respectively. PI staining was done according to Aresta-Branco et al. (2016). For DCV staining, cell suspensions were washed, and 0.5 μL DCV was added per each million isolated parasites and incubated for 10 min at 37°C. PI, DCV, and GFP intensities were measured with BD LSRFortessa cell analyzer.

RNA-Seq

RNA and cDNA library of both blood and gonadal fat depot from days 4 and 6 of infection, respectively, were prepared as described (Pena et al., 2014), and samples sequenced in an Illumina HiSeq2000 platform. Reads were processed and mapped to the *T. brucei* TREU927 genome. Differential gene expression was analyzed, and genes were considered differentially expressed if they were detected by at least two of the three considered algorithms (p adjusted < 0.01).

Myristate Metabolic Labeling

To evaluate myristate incorporation and metabolism, the fat isolation protocol was performed in lipid-free minimum essential medium (MEM). Parasites were placed in a vented tube with 1 mL MEM and starved for 30 min at 37°C. Starved parasites were then labeled with 0.4 mg of radiolabeled D_{27}-C14:0 pre-coupled with defatted BSA for 1 hr. A total of 450 μL of the cell suspension was washed, snap frozen in liquid nitrogen, and lyophilized in glass vials (pulse sample). The remaining parasites were re-suspended in 500 μL MEM and 100 μL HMI11 for 1 hr at 37°C, and at the end processed as for pulse sample (chase sample). Metabolite extraction, identification, and quantification were conducted as described in Oyola et al. (2012), with the exception that fatty acids were released by acid hydrolysis (200 μL 6M HCl at 110°C for 16 hr).

Statistical Analysis

Statistical analyses were performed by fitting LME models with mice as random effects unless otherwise indicated. At least three independent experiments were considered in each case and statistical significance was set to $\alpha = 0.05$ level. Data were analyzed after logarithm transformation.

ACCESSION NUMBERS

The accession number for Lister427 culture parasites is ArrayExpress: E-MTAB-1715. Sequence data generated as part of this study have been submitted to the ArrayExpress database (EMBL-EBI) under accession number ArrayExpress: E-MTAB-4061.

SUPPLEMENTAL INFORMATION

Supplemental Information includes Supplemental Experimental Procedures, seven figures, three tables, and one movie and can be found with this article online at http://dx.doi.org/10.1016/j.chom.2016.05.002.

AUTHOR CONTRIBUTIONS

S.T., F.R.-F., T.K.S., and L.M.F. designed the experiments and wrote the paper; S.T., F.R.-F., T.C., F.G., F.A.-B., F.B., A.P., and T.K.S. conducted the experiments; D.P.-N. and S.A.Y. analyzed the RNA-seq data; J.V.D.A. designed and conducted experiments with tsetse flies; R.M.R. conducted the statistical analysis; and S.D. designed experiments.

ACKNOWLEDGMENTS

The authors thank Keith Matthews (University of Edinburgh) for providing AnTat1.1^{E} clone, Christian Janzen (University of Wurzburg) for the *GFP::PAD1$_{utr}$* cell line, Dave Barry (University of Glasgow) for critical reading of the manuscript, Catarina Gadelha (University of Nottingham) for guidance in electron microscopy studies, Rita Ventura (ITQB) for support with lyophilization of samples, João Rodrigues (iMM) and Eugénia Carvalho (University of Coimbra) for useful discussions of the data, Leonor Pinho and Margarida Vaz for help with mouse work, and Fernando Augusto for drawing the graphical abstract. The authors would like to acknowledge the Rodent Facility, the Histology and Comparative Pathology and Bioimaging Laboratories of the Instituto de Medicina Molecular, and all members of the L.M.F. lab. This work was supported by 55007419 (HHMI) and 2151 (EMBO) to L.M.F., D.P.-N., F.B., and F.G.; FCT fellowships to S.T., F.R.-F., and F.A.-B. (SFRH/BPD/89833/2012, SFRH/BD/51286/2010, and SFRH/BD/80718/2011, respectively); Wellcome Trust grant (093228), MRC MR/M020118/1, and European Community Seventh Framework Programme under grant agreement No. 602773 (Project KINDRED) to S.A.Y. and T.K.S.; and PAI 7/41 (Belspo) and ERC-NANOSYM to J.V.D.A.

Received: December 10, 2015
Revised: March 21, 2016
Accepted: April 30, 2016
Published: May 26, 2016

REFERENCES

Amole, B.O., Wittner, M., Hewlett, D., and Tanowitz, H.B. (1985). *Trypanosoma brucei*: infection in murine diabetes. Exp. Parasitol. *60*, 342–347.

Aresta-Branco, F., Pimenta, S., and Figueiredo, L.M. (2016). A transcription-independent epigenetic mechanism is associated with antigenic switching in *Trypanosoma brucei*. Nucleic Acids Res. *44*, 3131–3146.

Bargul, J.L., Jung, J., McOdimba, F.A., Omogo, C.O., Adung'a, V.O., Krüger, T., Masiga, D.K., and Engstler, M. (2016). Species-specific adaptations of *Trypanosome* morphology and motility to the mammalian host. PLoS Pathog. *12*, e1005448.

Butter, F., Bucerius, F., Michel, M., Cicova, Z., Mann, M., and Janzen, C.J. (2013). Comparative proteomics of two life cycle stages of stable isotope-labeled *Trypanosoma brucei* reveals novel components of the parasite's host adaptation machinery. Mol. Cell. Proteomics *12*, 172–179.

Chun, T.W., Moir, S., and Fauci, A.S. (2015). HIV reservoirs as obstacles and opportunities for an HIV cure. Nat. Immunol. *16*, 584–589.

Claes, F., Vodnala, S.K., van Reet, N., Boucher, N., Lunden-Miguel, H., Baltz, T., Goddeeris, B.M., Büscher, P., and Rottenberg, M.E. (2009). Bioluminescent imaging of *Trypanosoma brucei* shows preferential testis dissemination which may hamper drug efficacy in sleeping sickness. PLoS Negl. Trop. Dis. *3*, e486.

Coppens, I., Opperdoes, F.R., Courtoy, P.J., and Baudhuin, P. (1987). Receptor-mediated endocytosis in the bloodstream form of *Trypanosoma brucei*. J. Protozool. *34*, 465–473.

Das, A., Morales, R., Banday, M., Garcia, S., Hao, L., Cross, G.A., Estevez, A.M., and Bellofatto, V. (2012). The essential polysome-associated RNA-binding protein RBP42 targets mRNAs involved in *Trypanosoma brucei* energy metabolism. RNA *18*, 1968–1983.

de Jesus, T.C., Tonelli, R.R., Nardelli, S.C., da Silva Augusto, L., Motta, M.C., Girard-Dias, W., Miranda, K., Ulrich, P., Jimenez, V., Barquilla, A., et al. (2010). Target of rapamycin (TOR)-like 1 kinase is involved in the control of polyphosphate levels and acidocalcisome maintenance in *Trypanosoma brucei*. J. Biol. Chem. *285*, 24131–24140.

Fernandes, J.H., Atouguia, J.M., Peleteiro, M.C., Jennings, F.W., and Rosário, V.E. (1997). Post-treatment hind-leg paralysis in mice infected with *Trypanosoma brucei brucei:* a light microscopic study. Acta Trop. *63*, 179–184.

Ferreira, A.V., Segatto, M., Menezes, Z., Macedo, A.M., Gelape, C., de Oliveira Andrade, L., Nagajyothi, F., Scherer, P.E., Teixeira, M.M., and Tanowitz, H.B. (2011). Evidence for Trypanosoma cruzi in adipose tissue in human chronic Chagas disease. Microbes Infect. *13*, 1002–1005.

Franke-Fayard, B., Janse, C.J., Cunha-Rodrigues, M., Ramesar, J., Büscher, P., Que, I., Löwik, C., Voshol, P.J., den Boer, M.A., van Duinen, S.G., et al. (2005). Murine malaria parasite sequestration: CD36 is the major receptor, but cerebral pathology is unlinked to sequestration. Proc. Natl. Acad. Sci. USA *102*, 11468–11473.

Giroud, C., Ottones, F., Coustou, V., Dacheux, D., Biteau, N., Miezan, B., Van Reet, N., Carrington, M., Doua, F., and Baltz, T. (2009). Murine models for *Trypanosoma brucei* gambiense disease progression—from silent to chronic infections and early brain tropism. PLoS Negl. Trop. Dis. *3*, e509.

Gull, K. (1999). The cytoskeleton of trypanosomatid parasites. Annu. Rev. Microbiol. *53*, 629–655.

Kennedy, P.G. (2013). Clinical features, diagnosis, and treatment of human African trypanosomiasis (sleeping sickness). Lancet Neurol. *12*, 186–194.

Kremer, J.R., Mastronarde, D.N., and McIntosh, J.R. (1996). Computer visualization of three-dimensional image data using IMOD. J. Struct. Biol. *116*, 71–76.

Losos, G.J., and Ikede, B.O. (1972). Review of pathology of diseases in domestic and laboratory animals caused by *Trypanosoma congolense, T. vivax, T. brucei, T. rhodesiense* and *T. gambiense*. In Veterinary Pathology, D.C. Dodd, ed. (S. Karger), pp. 1–71.

MacGregor, P., Savill, N.J., Hall, D., and Matthews, K.R. (2011). Transmission stages dominate trypanosome within-host dynamics during chronic infections. Cell Host Microbe *9*, 310–318.

MacGregor, P., Szöőr, B., Savill, N.J., and Matthews, K.R. (2012). Trypanosomal immune evasion, chronicity and transmission: an elegant balancing act. Nat. Rev. Microbiol. *10*, 431–438.

McLatchie, A.P., Burrell-Saward, H., Myburgh, E., Lewis, M.D., Ward, T.H., Mottram, J.C., Croft, S.L., Kelly, J.M., and Taylor, M.C. (2013). Highly sensitive in vivo imaging of *Trypanosoma brucei* expressing “red-shifted” luciferase. PLoS Negl. Trop. Dis. *7*, e2571.

Mugnier, M.R., Cross, G.A., and Papavasiliou, F.N. (2015). The in vivo dynamics of antigenic variation in *Trypanosoma brucei*. Science *347*, 1470–1473.

Neyrolles, O., Hernández-Pando, R., Pietri-Rouxel, F., Fornès, P., Tailleux, L., Barrios Payán, J.A., Pivert, E., Bordat, Y., Aguilar, D., Prévost, M.C., et al. (2006). Is adipose tissue a place for Mycobacterium tuberculosis persistence? PLoS ONE *1*, e43.

Ouchi, N., Parker, J.L., Lugus, J.J., and Walsh, K. (2011). Adipokines in inflammation and metabolic disease. Nat. Rev. Immunol. *11*, 85–97.

Oyola, S.O., Evans, K.J., Smith, T.K., Smith, B.A., Hilley, J.D., Mottram, J.C., Kaye, P.M., and Smith, D.F. (2012). Functional analysis of *Leishmania* cyclopropane fatty acid synthetase. PLoS ONE *7*, e51300.

Pena, A.C., Pimentel, M.R., Manso, H., Vaz-Drago, R., Pinto-Neves, D., Aresta-Branco, F., Rijo-Ferreira, F., Guegan, F., Pedro Coelho, L., Carmo-Fonseca, M., et al. (2014). *Trypanosoma brucei* histone H1 inhibits RNA polymerase I transcription and is important for parasite fitness in vivo. Mol. Microbiol. *93*, 645–663.

Ranjithkumar, M., Malik, T.A., Saxena, A., Dan, A., Sakthivel, P.C., and Dey, S. (2013). Hyperlipidaemia in trypanosomiasis of naturally infected horses: possible cachexia-anorexia syndrome? Trop. Anim. Health Prod. *45*, 417–421.

Richardson, J.B., Evans, B., Pyana, P.P., Van Reet, N., Sistrom, M., Büscher, P., Aksoy, S., and Caccone, A. (2016). Whole genome sequencing shows sleeping sickness relapse is due to parasite regrowth and not reinfection. Evol. Appl. *9*, 381–393.

Siegel, T.N., Gunasekera, K., Cross, G.A., and Ochsenreiter, T. (2011). Gene expression in *Trypanosoma brucei*: lessons from high-throughput RNA sequencing. Trends Parasitol. *27*, 434–441.

Szöör, B., Haanstra, J.R., Gualdrón-López, M., and Michels, P.A. (2014). Evolution, dynamics and specialized functions of glycosomes in metabolism and development of trypanosomatids. Curr. Opin. Microbiol. *22*, 79–87.

Taylor, A.E., Lanham, S.M., and Williams, J.E. (1974). Influence of methods of preparation on the infectivity, agglutination, activity, and ultrastructure of bloodstream trypanosomes. Exp. Parasitol. *35*, 196–208.

Tyler, K. (1998). Differentiation and division of *Trypanosoma brucei* in the mammalian bloodstream. PhD thesis (Manchester: University of Manchester).

Tyler, K.M., Matthews, K.R., and Gull, K. (2001). Anisomorphic cell division by African trypanosomes. Protist *152*, 367–378.

van Hellemond, J.J., and Tielens, A.G. (2006). Adaptations in the lipid metabolism of the protozoan parasite *Trypanosoma brucei*. FEBS Lett. *580*, 5552–5558.

Vassella, E., Den Abbeele, J.V., Bütikofer, P., Renggli, C.K., Furger, A., Brun, R., and Roditi, I. (2000). A major surface glycoprotein of *trypanosoma brucei* is expressed transiently during development and can be regulated post-transcriptionally by glycerol or hypoxia. Genes Dev. *14*, 615–626.

Wheeler, R.J., Gull, K., and Gluenz, E. (2012). Detailed interrogation of trypanosome cell biology via differential organelle staining and automated image analysis. BMC Biol. *10*, 1.

Wheeler, R.J., Gluenz, E., and Gull, K. (2013). The limits on trypanosomatid morphological diversity. PLoS ONE *8*, e79581.

Cell Host & Microbe, Volume *19*

Supplemental Information

Trypanosoma brucei Parasites Occupy and Functionally Adapt to the Adipose Tissue in Mice

Sandra Trindade, Filipa Rijo-Ferreira, Tânia Carvalho, Daniel Pinto-Neves, Fabien Guegan, Francisco Aresta-Branco, Fabio Bento, Simon A. Young, Andreia Pinto, Jan Van Den Abbeele, Ruy M. Ribeiro, Sérgio Dias, Terry K. Smith, and Luisa M. Figueiredo

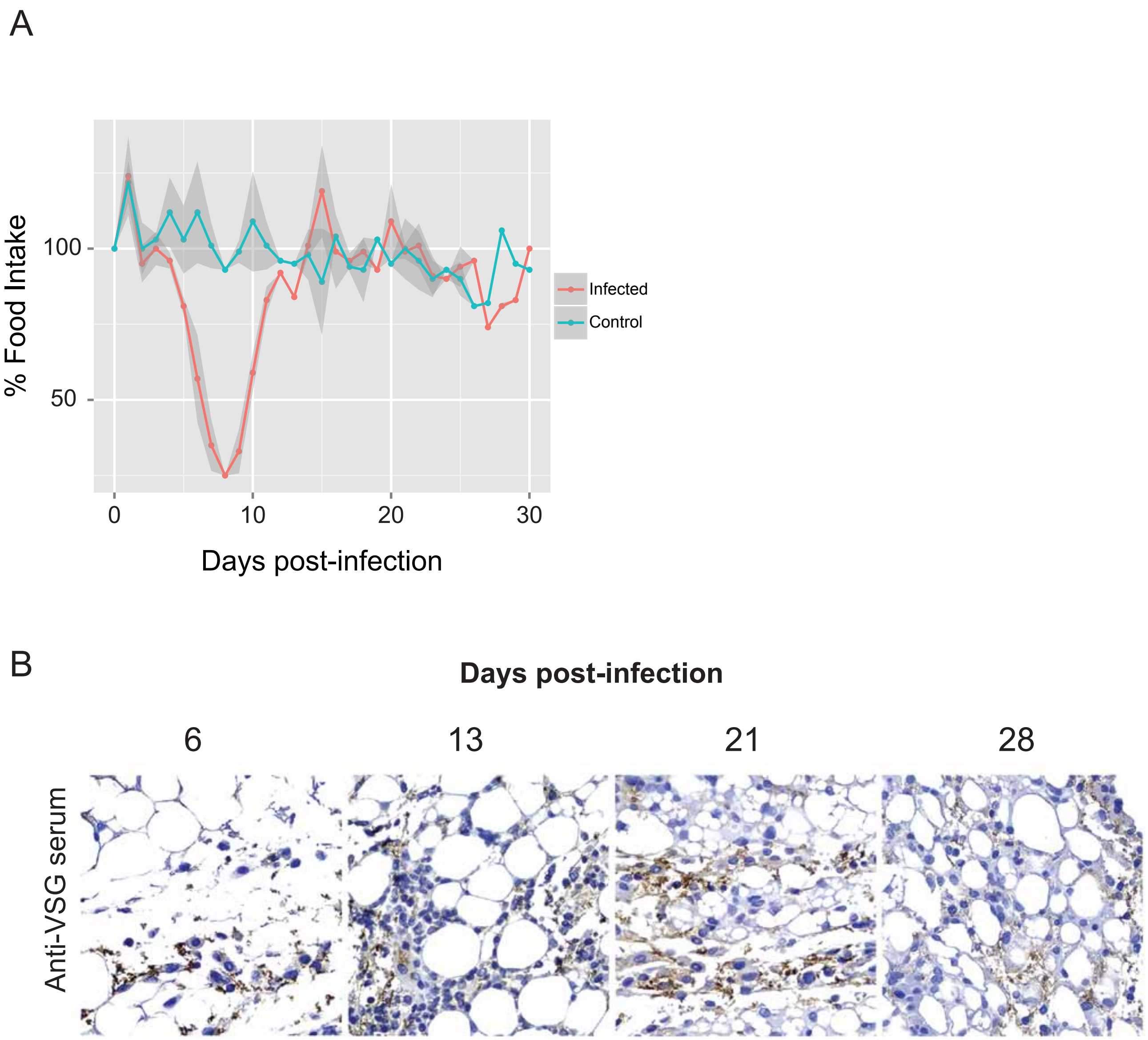

Figure S1. Related to Figure 1 and Figure 2. Clinical and histology details during *T. brucei* infection.

C57BL/6J mice were injected i.p. with 2000 AnTat1.1E parasites.
(A) Variation of food intake during infection. Animals (n = 15 per group) were group housed and food intake was measured daily by weighting the food and dividing by the number of mice per cage (n = 4 per condition). Light grey shaded area represents SEM.
(B) Representative brightfield micrographs of gonadal fat depots at different days post-infection, assessed by immunohistochemistry with anti-VSG antibody (parasites appear in brown). Original magnification, 400x.

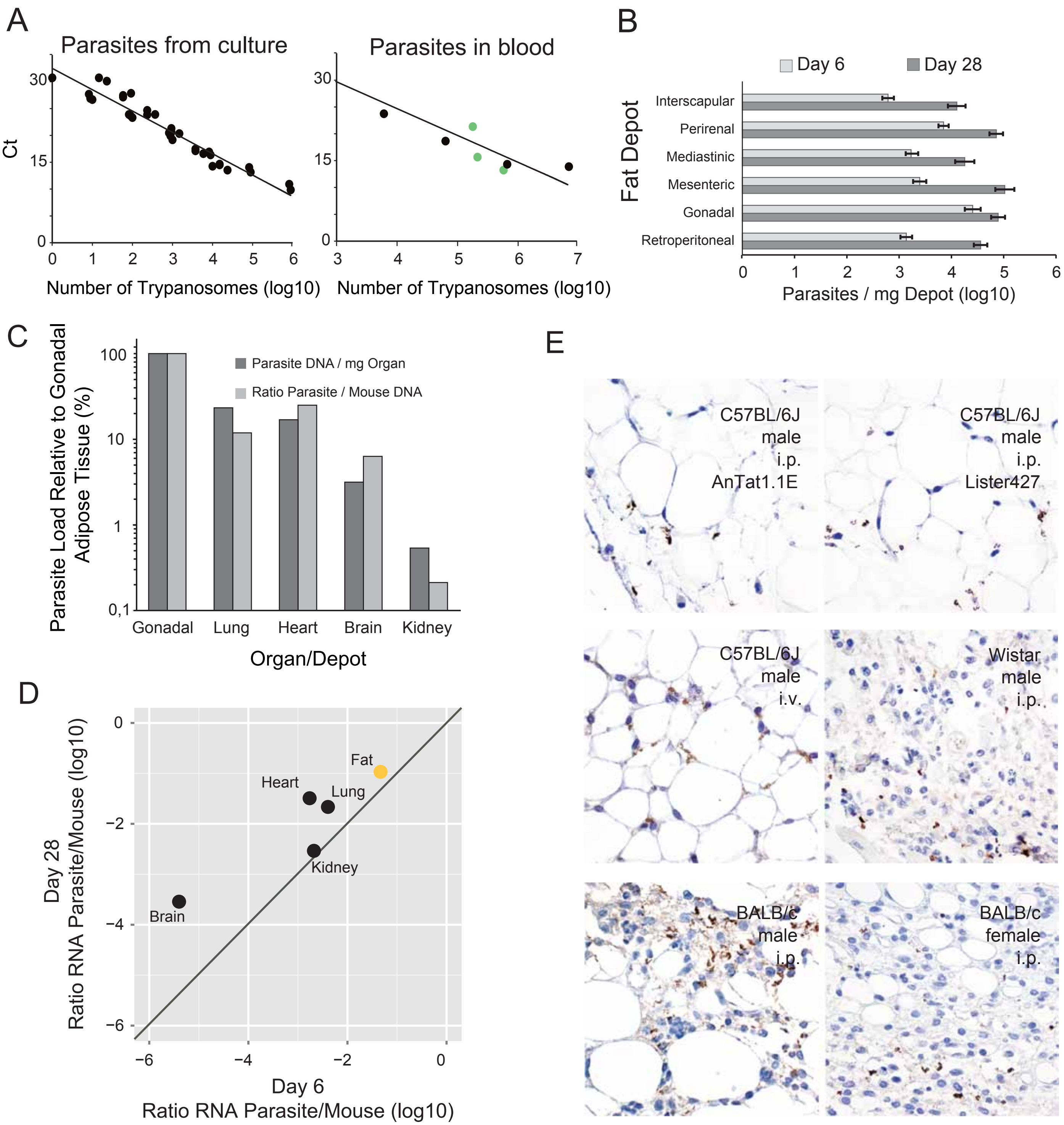
A
Parasites from culture
Ct
Number of Trypanosomes (log10)
Parasites in blood
Number of Trypanosomes (log10)
B
Day 6
Day 28
Fat Depot
Interscapular
Perirenal
Mediastinic
Mesenteric
Gonadal
Retroperitoneal
Parasites / mg Depot (log10)
C
Parasite Load Relative to Gonadal Adipose Tissue (%)
Parasite DNA / mg Organ
Ratio Parasite / Mouse DNA
Gonadal
Lung
Heart
Brain
Kidney
Organ/Depot
D
Day 28
Ratio RNA Parasite/Mouse (log10)
Fat
Heart
Lung
Kidney
Brain
Day 6
Ratio RNA Parasite/Mouse (log10)
E
C57BL/6J male i.p. AnTat1.1E
C57BL/6J male i.p. Lister427
C57BL/6J male i.v.
Wistar male i.p.
BALB/c male i.p.
BALB/c female i.p.

Figure S2. Related to Figure 1 and 2. Further validation of preferential accumulation of parasites in adipose tissue.

(A) Calibration curve obtained from four independent *in vitro* cultures of known cell density. gDNA was extracted, serially diluted and amplified by quantitative PCR using *T. brucei* 18S rDNA primers. The goodness of fit of the linear regression is $R^2 = 0.933$. Calibration curve obtained from blood from infected mice (n = 3) (green dots) and from a culture of parasites diluted in blood from naive mice (n = 1) (black dots). The goodness of fit of the linear regression is $R^2 = 0.925$.
(B) Parasite density in six fat depots on day 6 (n = 6-12) and 28 (n = 3-6) post-infection determined by qPCR of gDNA. For each depot, significant differences were found between days 6 and 28 post infection (Student's unpaired t test, $P < 0.05$).
(C) Two different gDNA qPCR methods were compared on day 28 post-infection: i. number of parasites per mg of organ (n = 3 - 9) and ii. ratio between *T. brucei and* mouse 18S gDNA (n = 3 - 9). Both methods show a similar parasite density in different tissues (LME, P = 0.72).
(D) Parasite density on day 6 and 28 post-infection determined by qRT-PCR of RNA. Transcripts of the parasite *TbZFP3* gene were normalized to the mouse *Gapdh*. Each point represents the geometric mean of the parasite density on day 6 (n = 3 - 4) and on day 28 post-infection (n = 3 - 5). RNA quantification validates the conclusions taken from gDNA qPCR: the adipose tissue is the major parasite reservoir (LME, P = 0.0006). The relative contribution of other organs is similar to what was measured by gDNA, except for brain, in which parasite density was lower than expected. Perhaps *TbZFP3* is downregulated in this organ.
(E) Representative brightfield micrographs of *T. brucei* in gonadal adipose tissue in different models of infection, assessed by immunohistochemistry with anti-VSG antibody (parasites appear in brown). Original magnification, 400x.

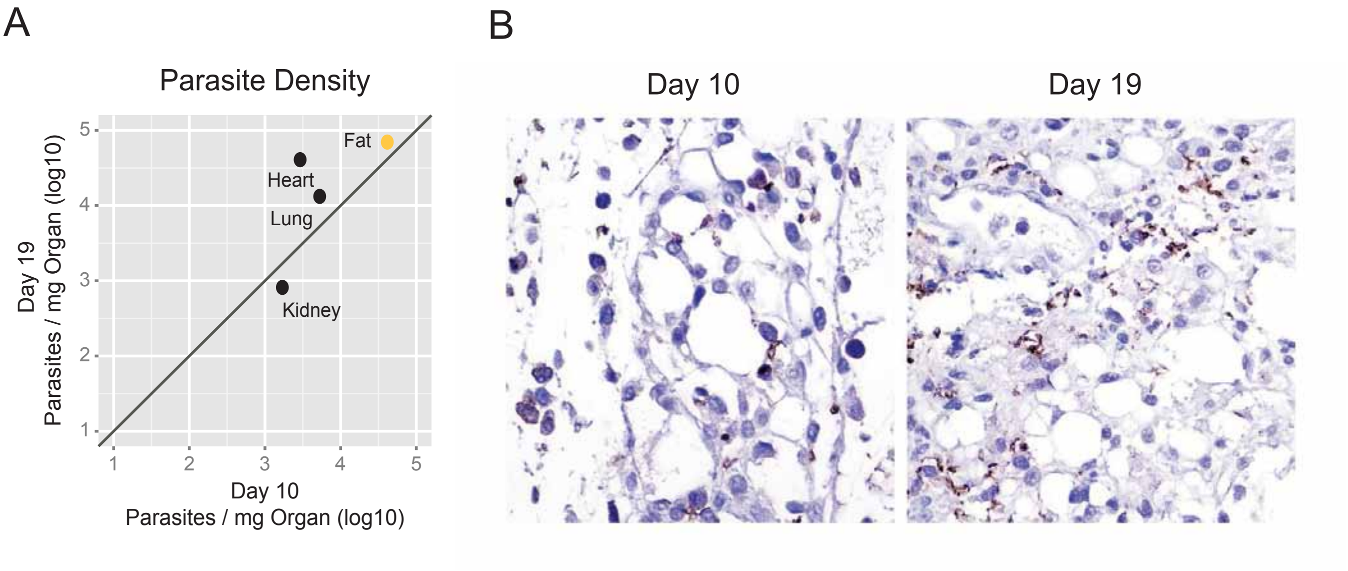

Figure S3. Related to Figure 2. Parasites accumulate in fat when infection is initiated by tsetse bite.

(A) Parasite density in multiple organs in mice naturally infected by the bite of a tsetse fly. Mice were sacrificed at the first and second peaks of parasitemia (10 and 19 days post-infection respectively) and parasite density determined by amplification of gDNA, as described in Figure 2. Each point represents the geometric mean of the parasite density at day 10 (n = 4) and at day 19 (n = 8).

(B) Representative brightfield micrographs of gonadal fat depot immunostained with anti-VSG serum, 10 and 19 days post-infection by tsetse bite. Original magnification, 40x.

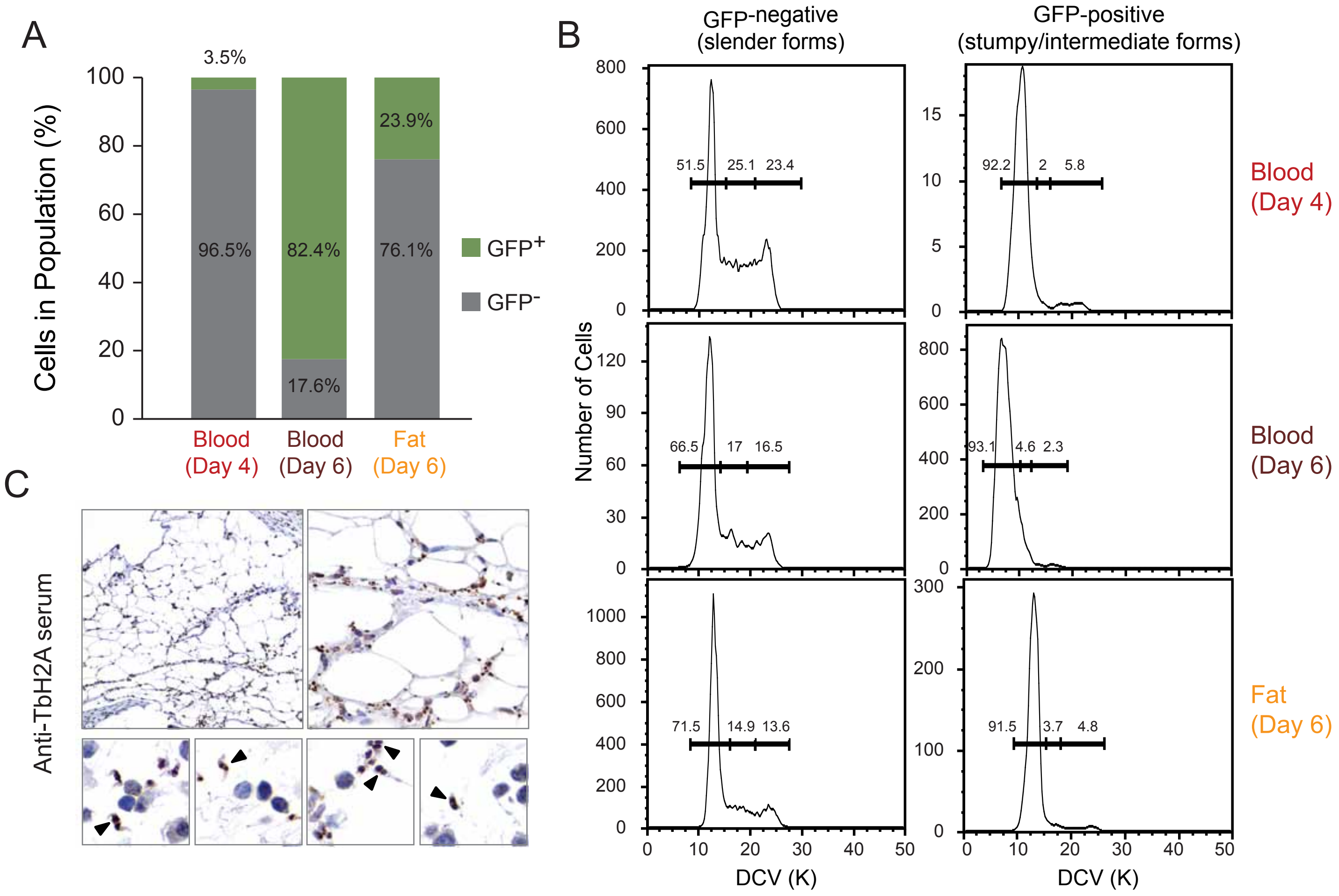

Figure S4. Related to Figure 3. Complementary methods to confirm presence of both replicative and cell-cycle arrested parasites in fat on day 6 post-infection.

(A) GFP Expression and (B) Cell cycle analysis in parasites isolated from blood and adipose tissue, 4 or 6 days post-infection, assayed by FACS. A *GFP::PAD1$_{3UTR}$ T. brucei* reporter cell-line was stained with DyeCycle Violet. The histograms represent the distribution and the percentage of parasites in each cell cycle stage. DyeCycle Violet staining validates the conclusions taken from propidium iodide staining analysis.

(C) Representative brightfield micrographs of gonadal fat depots on day 6 post-infection, assessed by immunohistochemistry with anti-*T. brucei* H2A rabbit serum (parasites appear in brown). Original magnification, 20x, 40x and 100x. Arrowheads indicate parasites undergoing nuclear division.

A

GFP expression	Tissue	Length (µm)	Width (µm)
GFP-negative (slenders)	Fat	24.57 ± 2.99	2.12± 0.26
	Blood	24.39 ± 2.50	2.15 ± 0.26
GFP-positive (stumpy/interm.)	Fat	21.32 ± 2.73	2.29 ± 0.31
	Blood	18.43 ± 1.81	3.11 ± 0.38

n= 100, Numbers are mean values with ± standard deviations .

B

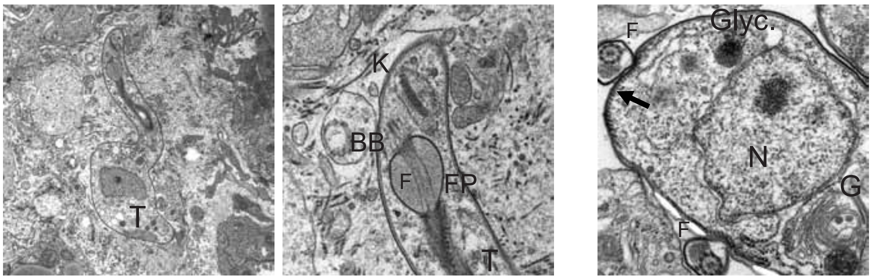

C

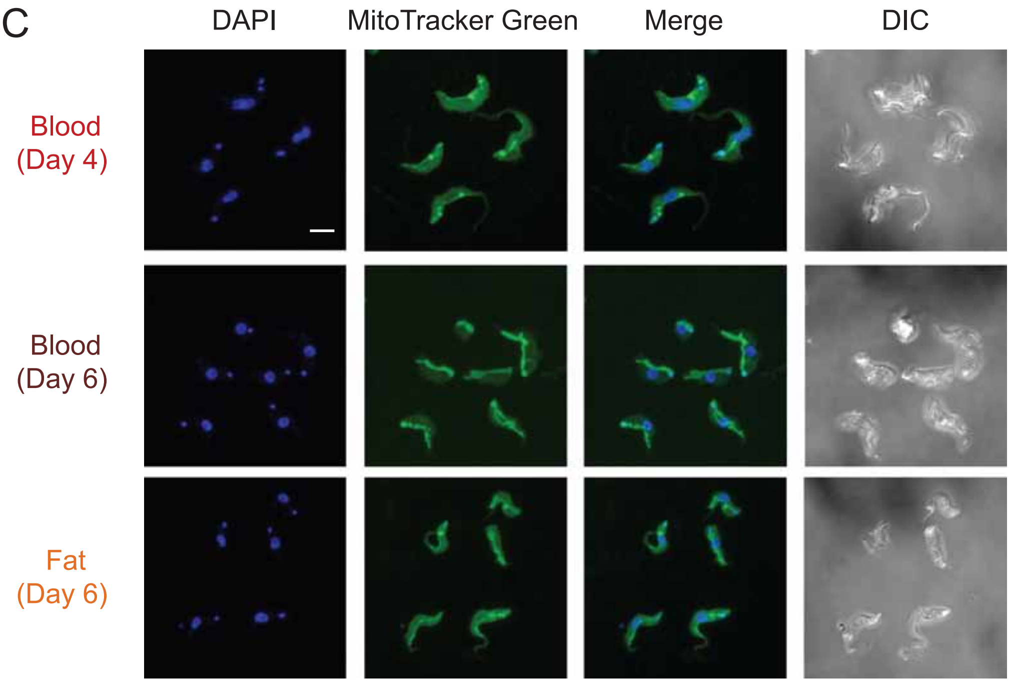

Figure S5. Related to Figure 4. Subcellular organization of parasites from adipose tissue.

(A) Length and width mean values of GFP-negative (slender) and GFP-positive (stumpy/intermediate) parasites isolated from blood or fat.
(B) Transmission electron micrographs of parasites in gonadal adipose tissue (day 28 post-infection).
T, trypanosome; K, kinetoplast; BB, basal body; F, flagellum; FP, flagellar pocket; N, nucleus; Glyc, Glycosomes; Arrow: subpellicular microtubules. Scale bars represent 2 and 0,5 µm in the left and right panels, respectively.
(C) MitoTracker Green, which stains in live cells the mitochondrion membrane, regardless of its membrane potential, was used to assess mitochondrion morphology. DNA was stained by DAPI and images were captured under a confocal microscope. Bloodstream form parasites from day 4 of infection showed a punctate pattern typical of mitochondrion in slender parasites, while on day 6, mitochondrion displayed a tubular structure with a few branches in what appeared to be stumpy forms. The mitochondrion of adipose tissue forms present a tubular structure, but with fewer branches and thinner. All panels are shown with the same magnification. Scale bar represents 5 µm.

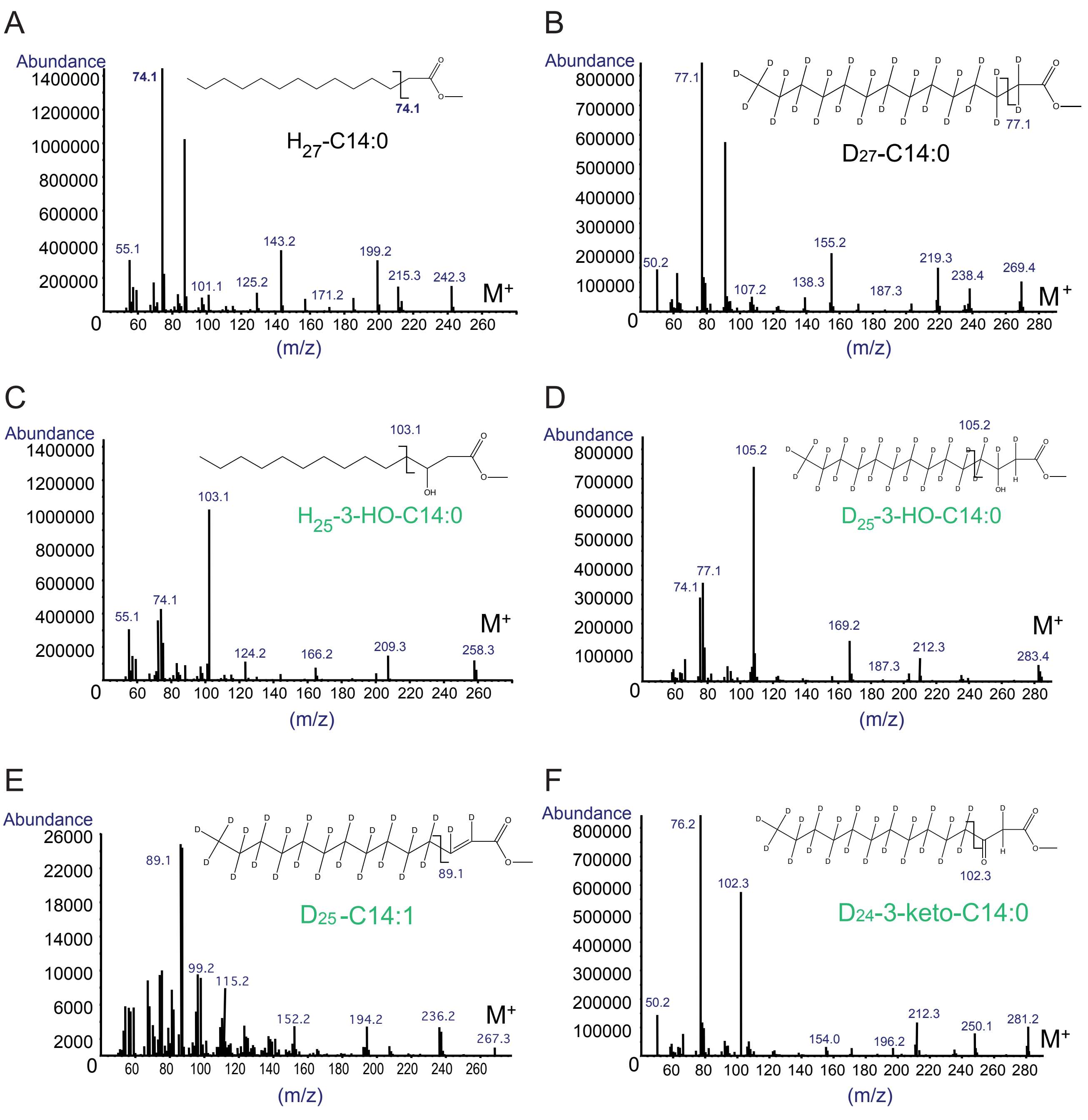

Figure S6. Related to Figure 6. Lipid metabolites identified by GC-MS.

All panels show the structure and fragmentation pattern of methyl ester derivatives of:

(A) myristic acid (C14:0)

(B) fully deuterated-myristic acid (D_{27}-C14:0)

(C) 3-hydroxy- myristic acid (3-HO-C14:0)

(D) 3-hydroxy-deuterated- myristic acid (D_{24}-3-HO-C14:0)

(E) deuterated-myristoleic acid (D_{25}-C14:1)

(F) 3-keto-deuterated- myristic acid (D_{24}-3-keto-C14:0)

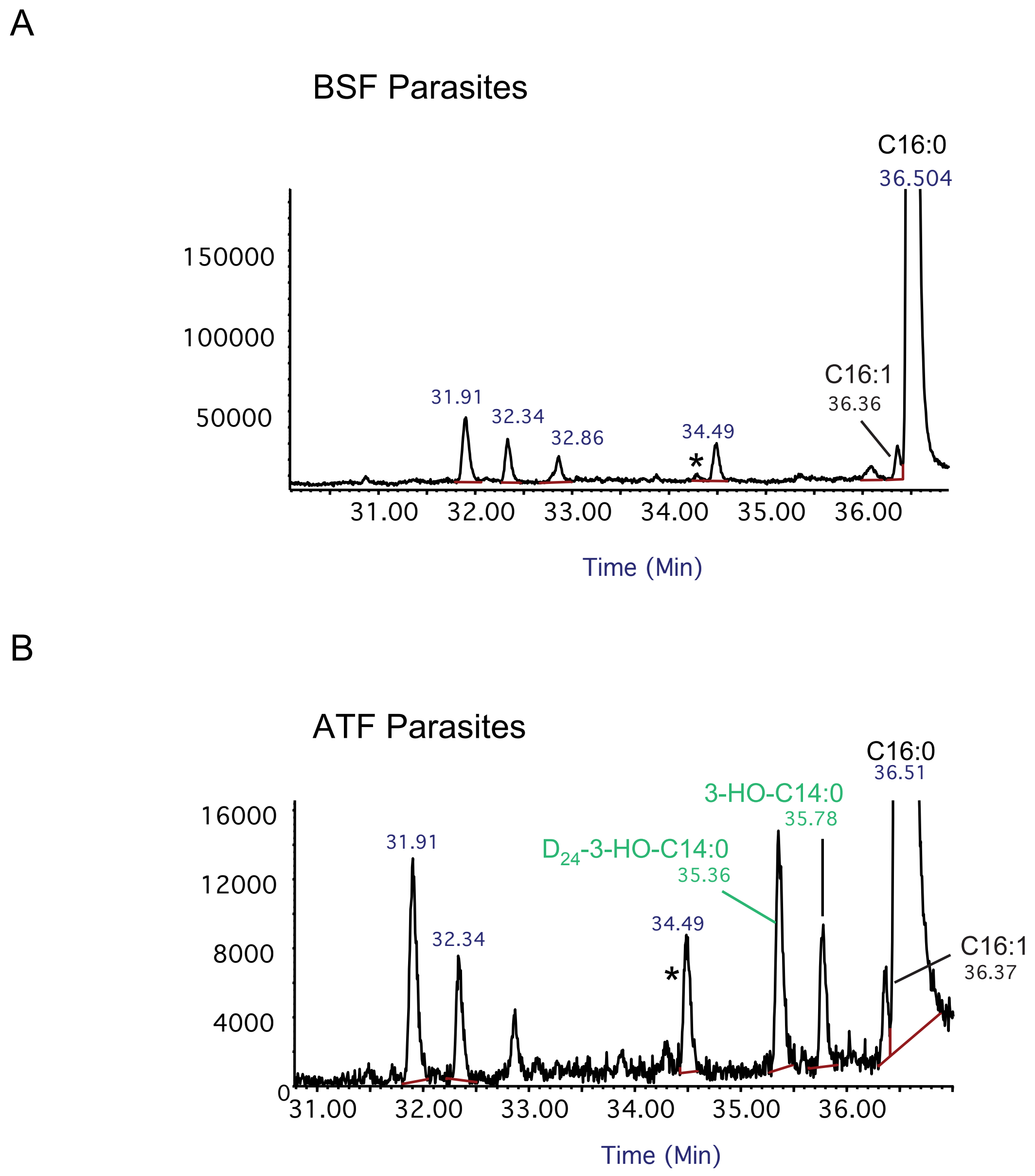

Figure S7. Related to Figure 6. Only ATF parasites produce hydroxyl-fatty acids as part of β-oxidation catabolism of fatty acids.
FAME analysis by GC-MS of D_{27}-Mys labeled and subsequently chased bloodstream (A) and adipose tissue (B) forms. Trace 31-37 minutes showing positions of (3-HO-C14:0) and (D_{24}-3-HO-C14:0) in adipose tissue forms only.

Supplemental Tables and Movies

Table S1, related to Figure 5. Mapping information of RNA-Seq reads in samples from blood and adipose tissue.

Sample	Total # reads	# of reads mapped to mouse genome	% of mouse reads in dataset	# of reads mapped to *T. brucei* genome	% of *T. brucei* reads in dataset
Blood-A	20 491 498	282 806	1,4%	16 004 171	78,1%
Blood-B	22 485 284	255 967	1,1%	18 418 295	81,9%
Fat-A	36 739 561	32 564 158	88,6%	3 396 819	9,2%
Fat-B	37 741 627	36 837 101	97,6%	415 936	1,1%
Fat-C	45 755 070	42 030 335	91,9%	3 311 276	7,2%

Table S2, related to Figure 5. Genes differentially expressed between parasites from adipose tissue and blood. (EXCEL file)

Table S3, related to Figure 5. Classes of transcripts differentially expressed between parasites in adipose tissue and blood. (EXCEL file)

Movie S1, related to Figure 4. Model constructed from hand-drawn contours marking the boundaries of cellular components in a tomogram of a trypanosome isolated from mouse gonadal adipose. The video is projected using Image J and various sub-cellular organelles are highlighted through subjectively attributed colors (plasma membrane in yellow; glycosomes in pink; nucleus in white; mitochondrion in green).

Supplemental Experimental Procedures

Ethical Statements

Animal experiments were performed according to EU regulations and approved by the Animal Ethics Committee of Instituto de Medicina Molecular (iMM) (AEC_2011_006_LF_TBrucei_IMM). The animal facility of iMM complies with the Portuguese law for the use of laboratory animals (Decreto-Lei 113/2013); and follows the European Directive 2010/63/EU and the FELASA (Federation of European Laboratory Animal Science Associations) guidelines and recommendations concerning laboratory animal welfare. The tsetse fly mediated *T. brucei* infection work was performed in compliance with the regulations for biosafety and animal ethics (VPU2014_1) and under approval from the Environmental administration of the Flemish government.

Cell Lines

The majority of the infections described in this manuscript were performed using *T. brucei* AnTat 1.1E, a pleomorphic clone derived from an EATRO1125 clone. AnTat 1.1E 90-13 is a transgenic cell-line encoding the tetracyclin repressor and T7 RNA polymerase (Engstler and Boshart, 2004). Tsetse infections were performed with *T. brucei* AnTAR1 strain. We also used *T. brucei* Lister 427, a monomorphic strain derived from antigenic type MiTat 1.2, clone 221a (Johnson and Cross, 1979). The stumpy reporter cell line *GFP::PAD1*$_{utr}$ derives from AnTat 1.1E 90-13 in which the green fluorescent protein, GFP, is coupled to PAD1 3'UTR. A nuclear localization signal (NLS) targets GFP protein into the nucleus. Prior to infection, *T. brucei* cryostabilates were thawed and parasite mobility was checked under an optic microscope.

Mice Infections

Mice were group-housed in filter-top cages and maintained in a Specific-Pathogen-Free barrier facility. The facility has standard laboratory conditions: 21 to 22°C ambient temperature and a 12 h light/12 h dark cycle. Chow and water were available *ad libitum*. Unless otherwise stated, all infections were performed in wild-type male C57BL/6J mice, 6-10 week old (Charles River, France), by intraperitoneally (i.p.) infection of 2000 *T. brucei* AnTat 1.1E 90-13 parasites.

To test if the presence of parasites in fat was dependent on the model, we performed the following variations in the infection protocol: a) male C57BL/6J mice were infected with a more virulent strain, the *T. b. brucei* strain Lister 427; b) male C57BL/6J mice were infected intravenously (i.v.) in the tail vein; c) a different strain of mice, BALB/c, and 7 week old Wistar rats (Charles River, France) were also infected (the latter with 4000 parasites); d) female C57BL/6J mice and finally e) male C57BL/6J mice naturally infected by tsetse bite. For this latter protocol, freshly emerged *Glossina morsitans morsitans* flies were fed their first blood meal on a *T. brucei* AnTAR1 infected mice at the peak of parasitemia. Subsequently, flies were maintained on commercially available defibrinated horse blood through *in vitro* membrane feeding. Thirty days after the infective blood meal, individual flies were evaluated for the presence of metacyclic trypanosomes in their salivary glands by salivation on pre-warmed (37°C) glass slides. To initiate a natural infection, one individual tsetse fly with a mature salivary gland infection was allowed to probe and feed per mouse.

Clinical Parameters and Organ Collection

All measurements in mice were made between 17:00 and 18:00. For parasite counts, blood samples were taken daily from the tail vein, and parasitemia was determined by manual counting using a Neubauer chamber. Organs/tissues of infected C57BL/6J mice (male and female) were collected at days 6, 13, 21 and 28 post-infection; for the infection with *T. b. brucei* strain Lister 427, organs were collected once parasitemia reached $1x10^8$ parasites/mL; for BALB/c mice, at day 6 post-infection; and for Wistar rats and C57BL/6J mice i.v. infections, at days 6, 13 and 20 post-infection. Animals were sacrificed by CO_2 narcosis, blood was collected by cardiac puncture and perfusion was performed to eliminate blood and parasites from circulation. Briefly, mice were perfused transcardially with pre-warmed heparinized saline (50 mL 1X phosphate buffered saline (1X PBS) with 250 μL of 5000 I.U./mL heparine per animal) using a peristaltic pump, ranging its speed from 2 mL/min to 8 mL/min. Organs were then collected and either used immediately for parasites isolation, snap frozen in liquid nitrogen for molecular analysis, or fixed in 10% neutral-buffered formalin for histopathology.

Transplantations

Donor mice were sacrificed with CO_2 narcosis (at days 21 and 28 post-infection) and the gonadal fat depot, brain, heart, and 600 µL of blood were harvested. Organs were manually homogenized through a 70 µm mesh into 1X PBS. Cell suspension was centrifuged at 1000 g for 10 minutes and resuspended in 800 µL of HMI11. Tissue lysates were injected intraperitoneally in naïve mice. Blood was diluted in 1X PBS and centrifuged for 5 minutes at 2800 g. Cell pellet was diluted in 800 µL of 1X PBS and injected intraperitoneally in naïve mice.

Histology

Formalin-fixed organs were embedded in paraffin and 3µm sections were stained with hematoxylin and eosin (H&E). For immunohistochemistry, 3µm sections were stained for VSG using non-purified rabbit sera anti-*T. brucei* VSG13 antigen (cross-reactive with most VSGs via the C-terminal domain) or anti-*T. brucei* H2A (generated against a recombinant protein) (kind gift of Christian Janzen), diluted 1:5000 and 1:3000, respectively. Briefly, antigen heat-retrieval was performed in a microwave oven (800 w) for 15 minutes with pH 9 Sodium Citrate buffer (Leica Biosystems, MO, USA). Incubation with ENVISION kit (Peroxidase/DAB detection system, Dako Corp, Santa Barbara, CA) was followed by Mayer's hemalumen counterstaining. No staining was observed in the negative control (without primary antibody). Tissue sections were examined by a pathologist (TC), blinded to experimental groups, in a Leica DM2500 microscope coupled to a Leica MC170 HD microscope camera.

For transmission electron microscopy, gonadal fat depot from infected mice (days 6 and 28 post-infection) was collected and fixed for three hours at 4ºC in 0.1 M cacodylate buffer, pH 7.4, containing 2.5% (v/v) glutaraldehyde. After staining for 1 hour with 1% (w/v) osmium tetroxide and 30 minutes with 1% (w/v) uranyl acetate, samples were dehydrated in an ethanol gradient (70-95-100%), transferred to propylene oxide and embedded in EPON™ resin. Semi-thin sections (300-400 nm) were stained with toluidine blue for light microscopy evaluation. Ultra-thin sections (70 nm) were collected in copper slot grids and stained with 2% uranyl acetate and lead citrate (Reynolds recipe). Grids were screened in a Hitachi H-7650 transmission electron microscope at 100 kV acceleration.

For 3D reconstruction of isolated trypanosomes, parasites isolated from gonadal fat depot were centrifuged at 5000 rpm and processed as described above for whole tissue. After embedding, approximately 26 serial ultra-thin sections (70 nm) were collected for each individual parasite. Grids of seven parasites were screened in a Hitachi H-7650 transmission electron microscope at 100 kV acceleration, serial section alignment was achieved using the IMOD software package version 4.7.3 for alignment and modeling (Kremer et al., 1996). ,Videos were projected using ImageJ 4.47v.

Microscopy analysis

Parasites from blood were isolated using a DEAE column (Taylor et al., 1974) and parasites from fat were isolated from gonadal fat depot by incubating the depot in HMI11 for up to 40 minutes and then purified from tissue debris over a DEAE column. For morphometric analysis, $1x10^6$ parasites were settled for 15 minutes to a pre-coated dish with 10% poly-Lysine, fixed with 2% paraformaldehyde for 10 minutes at room temperature, washed with 1X PBS and stained with diamidino-2-phenylindole (DAPI) (5 µg/mL in 1X PBS). Vectashield solution was used to mount the dish. Fluorescence and Phase Contrast images were acquired using Zeiss Cell Observer wide-field microscope. Parasite length was taken essentially as described in (Wheeler et al., 2012). Parasite width was manually scored in the nuclear region of the cell body.

For mitochondrial staining, isolated parasites were incubated in HMI-11 with 700 µM MitoTracker Green for 30 minutes at 37˚C. Excess of MitoTracker stain was removed and cells were chased in HMI-11 medium for 40 minutes at 37˚C. After washing with 1X PBS, cells were fixed and handled as above. Fluorescence and DIC images were acquired using a confocal Laser Point-Scanning Microscope (Zeiss LSM 710).

For tissue fluorescence analysis, the gonadal depot was stained with LipidTox (1:200 v/v in 1X PBS) for 30 minutes at 4ºc with agitation and then fixed in 10% neutral-buffered formalin, Sigma, and washed twice in 1 mL 1X PBS for 30 minutes at 37ºC with 150 rpm horizontal agitation. After, fat samples were embedded and mounted in Fluoromount-G. Fluorescence images were taken using a 40X objective in a Zeiss Cell Observer WF Microscope.

Parasite quantification in organs and tissues

Collected organs/tissues were snap frozen in liquid nitrogen. Genomic DNA (gDNA) was extracted from tissues using NZY tissue gDNA isolation kit (NZYTech, Portugal). The primers used for amplification of 18S rDNA gene of *T. brucei* were 5'–ACGGAATGGCACCACAAGAC–3' and 5'–GTCCGTTGACGGAATCAACC–3' and for the mouse 18S rDNA gene were 5'–TCGAGGCCCTGTAATTGGAA–3' and 5'–CTTTAATATACGCTATTGGAGCTGGAA–3'. By qPCR, the amount of *T. brucei* 18S rDNA present per milligram of organ/tissue were measured and converted into number of parasites using a calibration curve. The curve used to calculate parasite density in most solid organs/tissues was obtained by quantifying *T. brucei* 18S rDNA from serial dilutions of four independent cultures of AnTat1.1E 90-13 of known cell densities. Because PCR amplification from blood genomic DNA is sensitive to hemoglobin, we made a calibration curve specifically for blood samples, which was obtained by quantifying *T. brucei* 18S rDNA from blood from three infected mice of known parasitemia and from a known number of culture parasites diluted in blood. Parasite density in organs/tissues was calculated by dividing the number of parasites by the weight of organ/tissue used for qPCR, while total parasite load was calculated by multiplying the number of parasites/mg by the weight of organ/tissue at the corresponding day of infection. Blood density is about 1.05 kg/L, which means that 1 mL of blood is roughly equivalent 1050 mg. This conversion was taken into account to convert the number of parasites per mL of blood into number of parasites per mg of blood.

Parasite quantification from gDNA was also undertaken using ΔΔCt method. Here *T. brucei* and Mouse 18S rDNA genes were quantified from total genomic DNA. The ratio of the two genes provided a relative parasite density.

Given that trypanosomal gDNA can remain in circulation for up to 14 days, RNA qPCR was also used to quantify parasite load in tissues by calculating the ratio of *T. brucei TbZFP3* transcript to mouse *Gapdh* transcript. RNA was extracted using TRIzol or TRIzol LS reagents (Life Technologies) and cDNA prepared with TaqMan® Reverse Transcription Reagents (Invitrogen). The primers used for amplification of *TbZFP3* gene of *T. brucei* were 5'–CAGGGGAAACGCAAAACTAA–3' and 5'–TGTCACCCCAACTGCATTCT–3' and for mouse *Gapdh* gene were 5'–CAAGGAGTAAGAAACCCTGGACC–3' and 5'– CGAGTTGGGATAGGGCCTCT–3'. Quantitative PCR (qPCR) was performed on an ABI StepOnePlus real-time PCR machine and data was analyzed with the ABI StepOne software.

GFP Expression and Cell Cycle FACS Analysis

To quantify and characterize stumpy population in blood and fat, blood was collected by heart puncture from mice infected for 4 or 6 days, while gonadal fat depot was collected only on day 6 post infection (day 4 yield is too low to obtain enough isolated parasites). Parasites from blood were isolated using a DEAE column (Taylor et al., 1974) and parasites from fat were isolated from gonadal depot by incubating the depot in HMI11 for up to 40 minutes (hemocytometer was used to score the number of released parasites with time). GFP-expression in isolated parasites was analyzed on a BD LSRFortessa™ cell analyzer and data processed using FlowJo.

Cell-cycle analysis was performed using propidium iodide or Vybrant DyeCycle violet. For propidium-iodide, cells were first washed in ice-cold 1X PBS and then fixed with ice-cold 100% ethanol. After a washing step in 1X PBS, 0.5 μL of propidium iodide and 0.5 μL of RNaseA were added per each million of isolated parasites and incubated for 30 minutes at 37ºC. Vybrant DyeCycle violet staining was used in live cells. Essentially, cell suspensions from both blood and fat were washed once in 1X Trypanosome Dilution Buffer (1X TDB). 0.5 μL of DyeCycle violet was added per each million of isolated parasites and incubated for 10 minutes at 37ºC. Intensities were measured with BD LSRFortessa™ cell analyzer.

RNA-Sequencing

Blood from mice infected for 4 days was collected by heart puncture, parasites were purified over a DEAE column (Taylor et al., 1974) and total RNA extracted using TRIzol LS reagent. Mice infected for 6 days were sacrificed and perfused as explained above. Gonadal fat depot was collected and immediately lysed using TRIzol reagent. RNA and cDNA library preparation essentially followed the protocol in (Pena et al., 2014). Samples were sequenced in an Illumina HiSeq2000 platform (EMBL and BGI). Sequenced reads were 100bp paired-end for day 6 fat samples A and B, and 52bp single-end for the remaining samples. Paired-end reads were preprocessed by discarding the second read of each mate-pair, and trimming the first read to 52bp. Sequenced read quality was assessed using the FASTQC quality

control tool (http://www.bioinformatics.babraham.ac.uk/projects/fastqc/). The SolexaQA suite of programs (http://www.biomedcentral.com/1471-2105/11/485) (Cox et al., 2010) was used to trim raw reads to their longest contiguous segment above a PHRED quality threshold of 28, and reads smaller than 25 nucleotides long were discarded. Reads were mapped to the *T. brucei* TREU927 (TriTrypDB 8.0) and *Mus musculus* (GRCm38) genomes using Tophat 2 (REF: http://www.genomebiology.com /2013/14/4/R36/abstract) (Kim et al., 2013) with library type unstranded and the total number of reads mapped to each organism was assessed (Table S1). The number of reads mapped to each CDS was quantified in the R software environment using the function summarizeOverlaps from the GenomicAlignments (1.2.2). We found that approximately 0.1% of the reads could not be unambiguously assigned to the mouse or Trypanosome genome. To avoid a possible contamination of the Trypanosome differential expression results with reads originating from mouse RNA, all reads that mapped, uniquely or not, to the mouse genome were discarded for further analysis. Differential gene expression was analysed using DESeq2 (v1.6.3) (Anders and Huber, 2010), edgeR (v3.8.6) (Robinson et al., 2010) and baySeq (2.0.50) (Hardcastle and Kelly, 2010) from Bioconductor (v3.0). Genes were considered differentially expressed if they were detected by at least two of these three algorithms (P adjusted < 0.01). The ArrayExpress accession number for Lister427 culture parasites is E-MTAB-1715. Sequence data generated as part of this study have been submitted to the ArrayExpress database (EMBL-EBI) under accession number E-MTAB-4061.

Myristate Metabolic Labeling

To evaluate myristate incorporation and degradation by *T. brucei*, blood parasites were isolated using a DEAE column (Taylor et al., 1974) and fat parasites were isolated from gonadal depot by incubating it in lipid free 1X Minimum Essential Medium (MEM) for up to 40 minutes (hemocytometer was used to score the number of released parasites with time). MEM was chosen instead of HMI11 to ensure no external stimulus would be given to the parasites. After isolation, parasites were washed in MEM, resuspended in 1 mL of the same medium, placed in an open/vented tube and starved of fatty acids for 30 minutes while in a water bath at 37ºC with 100 rpm horizontal agitation. To the starved parasites, 200 µL of labeled myristate (D_{27}-myristic acid, CDN) pre-coupled with fatty acids free BSA were added. This solution was obtained by adding $2x10^{-3}$g of labeled myristate dissolved in 40 µL 100% ethanol (that was left at room temperature for 20 minutes) to $2x10^{-3}$g of fatty acids free BSA dissolved in 960 µL of MEM (that was left at 60ºC for 20 minutes), after a short spin the supernatant was ready for use. Parasites were once again incubated in a water bath at 37ºC with 100 rpm horizontal agitation for a one hour period (pulse). After pulse, 450 µL of the cell suspension were washed in 1 mL 1X TDB, snap frozen in liquid nitrogen and lyophilized in glass vials. The remaining parasites were centrifuged at 800 g for 5 minutes and re-suspended in 500 µL of MEM and 100 µL of HMI11. This cell suspension was incubated in a water bath at 37ºC with 100 rpm horizontal agitation for one hour (chase). Just as after pulse, cells were washed in 1X TDB and snap frozen in liquid nitrogen and lyophilized in glass vials. After, samples were spiked with an internal standard fatty acid C17:0, Sigma (20 µL of 1 mM) and dried under nitrogen. Acid hydrolysis was conducted using constant boiling HCl 6 M (200 µL) vortexing/sonication followed by incubation for 16 hours at 110 ºC. After cooling, the samples were evaporated to dryness in a speedvac concentrator and dried twice more from 200 µL of methanol:water (1:1). The protonated fatty acids were extracted by partitioning between 500 µL of HCl 20 mM and 500 µL of ether, the aqueous phase was re-extracted with 500 µL of fresh ether and the combined ether phases were dried under nitrogen in a glass vial. These fatty acids were then converted to methyl esters (FAME), by adding diazomethane (3 x 20 µL aliquots) to the dried residue, while on ice. After 30 minutes samples were allowed to warm to room temperature and left to evaporate to dryness in a fume hood. The FAME products were dissolved in 10-20 µL of dichloromethane and 1-2 µL analyzed by GC-MS on a Agilent Technologies (GC-6890N, MS detector-5973) with a ZB-5 column (30 M x 25 mm x 25 mm, Phenomenex), with a temperature program of at 70 ºC for 10 minutes followed by a gradient to 220 ºC at 5 ºC /minute and held at 220ºC for a further 15 minutes. Mass spectra were acquired from 50-550 amu. The FAME and the β-oxidation metabolites were identified based upon retention time and their fragmentation spectra.

Statistical Analysis

Statistical analyses were performed in the free software R: http://www.r-project.org. Statistically significant variation of body weight, food intake between infected and control mice and parasite density in multiple fat depots were determined by using Student's unpaired t test. Statistically significant variation of parasite morphology was determined by Wilcoxon rank sum test. To define the uniformity of the outcome of the two different parasites quantification methods we fitted a Linear Mixed Effects Model

(LME), using the nlme package in R, considering mice as random factors. The bias in parasite density and parasite load for fat relative to non-fat organs was also determined by fitting a linear mixed effect model, using the nlme package in R, considering mice as random factors. For the statistical analysis of RNA-Seq data, see RNA-Sequencing Section above.

Supplemental References

Cox, M. P., Peterson, D. A., and Biggs, P. J. (2010). SolexaQA: At-a-glance quality assessment of Illumina second-generation sequencing data. BMC Bioinformatics *11*, 485.

Engstler, M., and Boshart, M. (2004). Cold shock and regulation of surface protein trafficking convey sensitization to inducers of stage differentiation in *Trypanosoma brucei*. Genes Dev *18*, 2798-2811.

Hardcastle, T. J., and Kelly, K. A. (2010). baySeq: empirical Bayesian methods for identifying differential expression in sequence count data. BMC Bioinformatics *11*, 422.

Johnson, J. G., and Cross, G. A. M. (1979). Selective cleavage of variant surface glycoproteins from *Trypanosoma brucei.* Biochem J *178*, 689-697.

Kim, D., Pertea, G., Trapnell, C., Pimentel, H., Kelley, R., and Salzberg, S. L. (2013). TopHat2: accurate alignment of transcriptomes in the presence of insertions, deletions and gene fusions. Genome Biol *14*, R36.

Robinson, M. D., McCarthy, D. J., and Smyth, G. K. (2010). edgeR: a Bioconductor package for differential expression analysis of digital gene expression data. Bioinformatics *26*, 139-140.

Taylor, A. E., Lanham, S. M., and Williams, J. E. (1974). Influence of methods of preparation on the infectivity, agglutination, activity, and ultrastructure of bloodstream trypanosomes. Exp Parasitol *35*, 196-208.
